# Correction of vitamin D deficiency facilitated suppression of IP-10 and DPP IV levels in patients with chronic hepatitis C: A randomised double-blinded, placebo-control trial

**DOI:** 10.1371/journal.pone.0174608

**Published:** 2017-04-04

**Authors:** Piyawat Komolmit, Kriangsak Charoensuk, Kessarin Thanapirom, Sirinporn Suksawatamnuay, Panarat Thaimai, Chintana Chirathaworn, Yong Poovorawan

**Affiliations:** 1 Division of Gastroenterology and Hepatology, Department of Medicine, Faculty of Medicine, Chulalongkorn University and Center of Excellence in Liver Diseases: King Chulalongkorn Memorial Hospital, Thai Red Cross Society, Bangkok, Thailand; 2 Division of Gastroenterology, Department of Internal medicine, Buddhachinaraj Hospital School of Medicine, Phitsanulok, Thailand; 3 Division of Immunology, Department of Microbiology, Chulalongkorn university, Bangkok, Thailand; 4 Center of Excellence in Clinical Virology, Faculty of Medicine, Chulalongkorn University, Bangkok, Thailand; Kaohsiung Medical University, TAIWAN

## Abstract

Vitamin D deficiency was common among patients with chronic hepatitis C (CHC) and had negative influence on treatment outcome. Correction of vitamin D deficiency improved treatment response. Interferon gamma-induced protein 10 (IP-10) and enzyme dipeptidyl peptidase-4 (DPP IV) involved in inflammatory responses in CHC. Their higher levels at pretreatment of CHC could predict poorer responses. Vitamin D suppressed expression of IP-10 from monocytes *in vitro*. In CHC patients, DPP IV involved in IP-10 regulation. We hypothesized that correction of vitamin D insufficiency or deficiency in CHC patients might restore immune dysregulation through a pathway linked to the TH1/Th2 cytokines, IP-10 or DPP IV. We conducted a double-blind, placebo-controlled trial. 80 CHC patients with vitamin D levels less than 30 ng/mL were assigned to receive vitamin D (40) or placebo (40) supplements for 6 weeks. The levels of 25-hydroxyvitamin D [25(OH)D], Th1/Th2 cytokines, IP-10 and DPP IV were measured at baseline and at the 6th week. At the end of study, the mean 25(OH)D level in vitamin D group was significantly increased and normalised. There were no changes in the level of Th1/Th2 cytokines. Our important finding revealed that upon correction of vitamin D insufficiency or deficiency, the serum IP-10 and DPP IV levels were decreased significantly as compare to the placebo group (delta changes; 83.27 vs -133.80; 95% CI [-326.910, -40.758], p = 0.0125, and 271.04 vs -518.69; 95% CI [-1179,15, -59.781], p = 0.0305, respectively. As previous evidences suggested that each factor individually influenced and predicted outcome of CHC treatment. Our results offer a new insight and help to piece the puzzle of vitamin D deficiency, IP-10 and DPP IV together in CHC.

**Trial registration:** Thai Clinical Trials Registry TCTR20160429001

## Introduction

Over 170 million people worldwide were infected by hepatitis C virus (HCV) [1]. HCV is one of the major causes of chronic hepatitis, cirrhosis and hepatocellular carcinoma [1, 2]. The standard treatment with pegylated interferon (Peg-IFN) and ribavirin (RBV) leaded to sustained virological response (SVR) at least 80% of those with genotype 2 or 3. However, only a half of patients with HCV genotype 1 responded to the treatment [3]. Apart from viral factors, poor treatment outcomes were shown in immunocompromised patients and also in CHC patients with vitamin D deficiency [4, 5].

Arrays of imbalance in immunoregulatory cytokines happened during chronic HCV infection and permitted persistent HCV in host cells. T-helper cells play a crucial role in host responses to the virus. Dysregulation of TH1 and TH2 related cytokines and chemokines were evidenced during chronic HCV infection and theoretically caused viral persistent in host cells [6].

Vitamin D was demonstrated to involve in immune regulations both an innate and adaptive immunities, and also cell differentiation [7]. Vitamin D deficiency is one of the most common nutritional deficiency worldwide [8]. Several factors could lead vitamin D deficiency, such as lack of UV-B exposure in some regions of the world, liver and kidney dysfunctions and also some genetic variations of genes involved in vitamin D metabolic pathway [9]. Vitamin D deficiency was common among patients with chronic liver diseases and cirrhosis. The degree of deficiency is associated with severity of liver diseases [4].

The chemokine CXCL10 (interferon gamma-inducible protein 10, IP-10) was identified as an important serum marker predicting the outcome of treatment for CHC patients. The higher level of the IP-10 associated with lower responses to Peg-IFN/RBV treatment [10]. IP-10 levels were elevated in CHC patients comparing with healthy controls and correlated with higher HCV viral load, ALT elevations, and the extent of hepatic inflammation [11]. Genetic variances of *CXCL10* gene were demonstrated to have influence on the treatment outcome in CHC patients with unfavorable *IL-28B* genotypes [12]. IP-10 is a chemotactic factor produced by several tissues, including hepatocytes, involved in attracting T-lymphocytes, natural killer cells and monocytes to the sites of infection.

The link between vitamin D and IP-10 was previously demonstrated in an *in vitro* study. Treatment of human primary monocytes with vitamin D [1, 25(OH)_2_D] suppressed production of multiple inflammatory factors, including TNF alpha and IP-10 [13]. Recent study demonstrated a link between IP-10 and the enzyme dipeptidyl peptidase IV or CD26 in CHC patients. Cleaved by the DPP-IV, IP-10 is truncated into the antagonized form which was postulated as a mechanism associated with de-functionality of inflammatory responses in CHC [14].

We postulated that CHC patients who have vitamin D deficiency would be associated with lost in balances of adaptive immune responses to counteract with HCV infections. In addition, there might be a link between vitamin D deficiency and the changes in IP-10 and DPP IV cascade. To prove this concept, we conducted a randomised control-trial to assess the changes in serum levels T-helper cells associated cytokines, IP-10 and DPP-IV, without influences driven by interferon treatment, after a short-term period for correction of vitamin D deficiency in CHC patients.

## Materials and methods

### Patients and study design

#### Ethics statement

This study was reviewed and approved by the Ethics Committee, **Institutional Review Board** at the King Chulalongkorn Memorial hospital, Chulalongkorn university, Bangkok Thailand in accordance with the Declaration of Helsinki (1989) of the World Medical Association (IRB No. 523/54). The clinical trial registered number of the Thai Clinical Trials Registry (TCTR) which based on World Health Organisation criteria is register on 10 October 2016 under registration number: TCTR20160429001. We would like to apologize that we sincerely did not know regarding the trial register in advance before starting the study. We confirm that all ongoing and related trials for this intervention starting in January 2016 are registered. The trial was conducted between April 2012 and April 2013. After more information on DPP-IV and chronic hepatitis C emerging, addition analysis on DDP-IV was performed and finalized in June 2014. All participants provide their written informed consent, approved by the IRB, to participate in this study.

#### Study design and population

We conducted a randomized, double-blind, placebo-controlled, interventional study. A total of 93 CHC patients at King Chulalongkorn Memorial Hospital aged between 18 and 70 years old who agreed to participate were screened and included if they had vitamin D deficiency as defined by serum 25 OH vitamin D level below 30 ng/ml. The word “vitamin D deficiency” used in this study based on the term frequently used in the Endocrine Society Practice Guideline which, in more detail, represents the patients who had vitamin D insufficiency (vitamin D levels of 21–29 ng/mL) and deficiency (vitamin D levels below 20 ng/mL) [15]. Of the patients screened, 11 patients had no vitamin D deficiency and 2 patients with vitamin D deficiency decided not to take part in the clinical trial. The rest of the patients had no evidences of decompensated liver cirrhosis, human immunodeficiency viral infection, any type of autoimmune diseases, active viral or bacterial infections, history of steroid or immunosuppressive therapy, or history of interferon treatment within 12 months.

A total of 80 CHC patients were double blinded and randomized to receive either vitamin D or placebo supplement for 6 weeks. After the random codes were reviewed at the end of the 6th week, 40 patients had been in the vitamin D group and 40 patients had been in the placebo group (Fig 1). The strategic allocation of this study is demonstrated in Fig 1 as indicated by CONSORT 2010 [16]. The CONSORT 2010 checklist of information is available as supporting information (S1 File). Based on an Endocrine Society Clinical Practice Guideline, it requires at least 6–8 weeks to restore vitamin D levels by adequate oral vitamin D supplement [15]. We had a preliminary study on our designed vitamin D replacement protocol (see supplement) which give a successful result in all 10 CHC patients. In addition, we hypothesized that upon correction of the vitamin D deficiency, the immunological changes could begin within a short period, as our body immune responses are dynamic processes to fight against several thousand million of the virus generated per day. Therefore, we decided that 6-week assessment is an optimal time in our study.

**Fig 1:**
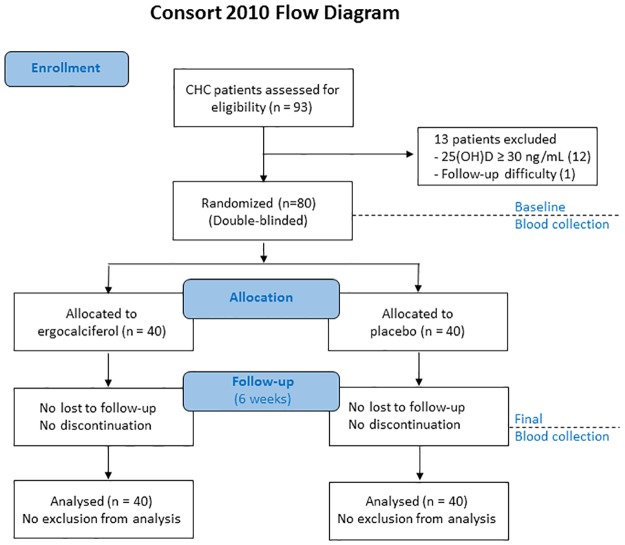
CONSORT 2010 flow diagram. CHC patients who were enrolled and followed up. 93 patients were screened for participation in the study. 13 patients were excluded. 80 patients were randomly assigned to receive a placebo or vitamin D supplements and followed up after six weeks of supplements. BMJ 2010;340:c869. doi:10.1136/bmj.c869.

#### Sample size calculation

To the best of our knowledge, there is no RCT based on VD supplement in CHC patients, especially focusing on serum IP-10. As most RCT of VD supplements require number of subjects less than 80 cases to show the changes in serum inflammatory markers’ levels [17]. We lack any of the important inputs required to formally power the study. Instead we thought that 80 subjects (the same as the number of wells for ELISA plate) was likely to yield as sufficient sample size to generate a clinically important effect as statistically significant. To ensure trivial effects are not identified as statistically significant, we added Cohen’s d to report effect size.

#### Randomisation

The randomization sequence was stratified with a 1:1 allocation using random block sizes of 4 based on computer generated method based (www.randomisation.com) and was performed by a research assistant without involvement in clinical trial. Details of the allocated group were given in sequentially numbered, opaque, sealed envelopes. After patient enrollment, the research assistant will open the envelope and inform stratified groups (A or B) to the investigators.

### Intervention

The vitamin D2 (Ergocalciferol) and placebo were prepared by a pharmacist in a capsule form and identical in appearance. They were prepacked in a bottle for 6-week supplement and consecutively numbered for each CHC patients according to the randomised results. All investigators and participants were blinded to type of medications and outcome measurements during the study period.

The dosages of ergocalciferol or vitamin D2 supplement were given according to our protocol based on the ranges of vitamin D deficiency as following: for mild deficiency (20 to less than 30 ng/mL) 60,000 unit/week, for moderate deficiency (10 to less than 20 ng/mL) 80,000 unit/week, and for severe deficiency (less than 10 ng/mL) 10,000 unit/week. Each vitamin D2 capsule contained 20,000 units. The total dosage was divided into 2 separate doses giving on Monday and Friday. The placebo and vitamin D2 capsules were made resemble and have the same weight. The detail protocols are deposited in supplements (S2 and S3 Files). No adverse events related to vitamin D or placebo supplement were reported in all patients during study period.

Clinical and demographic data of the patients were recorded including HCV genotypes and HCV viral load. Laboratory tests were collected at baseline and at the end of 6 weeks. These included liver function tests, and serum levels of 25-hydroxyvitamin D (25(OH)D), Th1/Th2 cytokines, IP-10 and DPP IV. Whole blood from each patient was collected, processed for serum, and stored at -80°C at each visit until used for analysis. The primary investigators, all study personnel, and all participants were blinded to the intervention. At the end of 6 weeks, any patients who had vitamin D deficiency were subjected for further vitamin D replacement as a standard medical care.

### Vitamin D assays

Vitamin D level was determined in serum samples with the Liaison 25 OH vitamin D total assay (DiaSorin, Saluggia, Italy) and was performed on the LIAISON^®^ chemiluminescent analyzer by using a method manufacturer introduced. The final concentration was indicated by ng/ml.

### Th-1/2 Cytokines quantitative analysis

Cytokine profiling was done by Bio-Plex Th-1/2 cytokine assay on the Bio-Plex suspension array system (Bio-Rad, Hercules, CA, USA) according to the manufacturer’s instructions. Cytokine quantitation was performed on bead-based ELISA using the Bio- Plex system (Bio-Rad). In brief, cell culture supernatant was mixed with beads having unique fluorescent intensity and coated with the antibody to various cytokines. Subsequently, the mixture was incubated with biotinylated anti-cytokine antibody. Finally, PE-conjugated streptavidin was added, and the fluorescent signal was detected using a Bio-Plex system (Bio-Rad). Raw data were initially measured as the relative fluorescence intensity and then converted to cytokine concentration based on a standard curve generated from the reference concentrations supplied in the kit. After result analysis, Cytokine levels were calculated using standard curve generated with known concentrations of cytokines. The data were analyzed using Bio-Plex Manager^™^ software with 5PL curve fitting. Cytokine levels were expressed in picogram per milliliter (pg/mL).

### Analysis of IP-10 quantification

The level of serum IP-10 was measured by using a Human IP-10 ELISA set kit (BD, Bioscience, San Diego, CA, USA) according to the manufacturer’s protocol. Serum IP-10 levels were measured using sandwich enzyme-linked immunosorbent duo kits according to the manufacturer’s instructions and expressed in pg/mL.

### Analysis of DPP IV concentration

The level of serum human DPP IV was quantified by using the quantitative sandwich enzyme immunoassay technique and used Human DPP IV/CD26 Immunoassay, Quantikine (R&D Systems, Minneapolis, MN, USA) with the plasma sample diluted in 1:5 in sample diluent according to the manufacturer’s instruction and expressed in ng/mL.

### Study end points

The primary end-point was to identify effects of vitamin D supplement on T-helper1/2 cytokines, IP-10 and DPP IV levels as compared to placebo in CHC patients.

### Statistical analysis

As there is no previous study on serum IP-10/DPP-4 levels in this regard, the sample size was an estimation for general study to assess immunological parameters. Baseline characteristics were compared using Pearson’s chi-square or Fisher’s exact tests and Wilcoxon’s rank sum tests, as appropriate. Dependent t-test or Wilcoxon Signed Rank test was employed for comparison between pre and post supplements, as appropriate. Comparison between two groups was performed through ANCOVA. In ANCOVA, the dependent variable is the post-test measure, and the pre-test measure was a covariate and controlled for. Pearson’s correlation coeffcient was used to describe the correlation between two continuous, normally distributed variables. P<0.05 was considered statistically significant. All statistical analyses were performed using SPSS version 16. In addition, Cohen’s d effect size power analysis was used for quantitative measurement of the magnitude of VD supplement on serum markers in between group. The values of 0.2, 0.5 and 0.8 represent the strength of small, medium and large effects [18]. The method of calculation is deposited in supplement (S4 File). All data set of this study is deposited in supplement (S5 File).

## Results

### Study patients at baseline

From January to December 2014, 80 patients were included into this study and randomly assigned to receive a placebo (n = 40) vitamin D supplement (n = 40). 42 patients were naïve cases without previous CHC treatment and 38 patients were previously failed therapy either relapsers or non-responders. Demographic and clinical characteristics were shown in Table 1. The mean age was 52.39 years (range; 30–70).

**Table 1:** Baseline characteristics of CHC patients in the placebo and vitamin D groups.

Variables	Placebo group (n = 40)	Vitamin D group (n = 40)	p values
Mean age (year)	52.2 ± 11.1	52.6 ± 8.5	0.830
Male gender (%)	20 (50)	23 (57.5)	0.654
HCV treatment status			0.403
• Naïve (%)	21 (52.5)	21 (52.5)	
• Previously failed (%)	19 (47.5)	19 (47.5)	
HCV genotypes			0.647
• Genotype 1, n (%)	18 (45.0)	19 (47.5)	
• Genotype 3, n (%)	16 (40.0)	13 (32.5)	
• Others (%), n (%)	6 (15.0)	8 (20.0)	
HCV viral load, log C/mL	5.72 ± 0.88	5.76 ± 0.84	0.822
FIB4 score, n (%)			0.559
• < 1.45	9 (22.5)	6 (15.0)	
• 1.45–3.25	19 (47.5)	23 (57.5)	
• > 3.25	12 (30.0)	11 (27.5)	
Median AST (IU/L), (range)	76.3 (16–323)	65.1 (21–323)	0.421
Median ALT (IU/L), (range)	78.5 (15–217)	72.1 (11–183)	0.560
Mean BMI	24.56 ± 3.98	24.48 ± 3.37	0.520
Mean serum levels* of			
• 25(OH)D**	20.27 ± 4.83	20.88 ± 5.40	0.596
• IP-10	665.61 ± 665.61	770.27 ± 642.02	0.180
• DPP IV**	6137.29 ± 1584.32	6551.06 ± 1757.14	0.387
• IL-2	13.07 ± 16.50	8.20 ± 24.30	0.245
• IL-4	3.93 ± 4.99	5.39 ± 5.39	0.246
• IL-5	4.69 ± 4.69	7.60 ± 11.95	0.157
• IL-10	7.56 ± 18.96	17.09 ± 44.51	0.216
• IL-12	29.25 ± 98.04	40.61 ± 111.33	0.629
• IL-13	4.68 ± 8.01	11.24 ±22.57	0.087
• IFN-γ	251.64 ± 334.47	328.31 ± 383.54	0.344
• TNF-α	602.45 ± 1374.21	651.52 ± 1267.72	0.869
• GM-CSF	21.96 ± 57.01	27.72 ± 43.74	0.614

* unit in pg/mL,

** unit in ng/mL

ALT, alanine aminotransferase; AST, aspartate aminotransferase; BMI, body mass index; IP-10, inducible protein-10; IL, interleukin; IFN-γ, interferon gamma; TNF-α, tumor necrosis factor alpha; GM-CSF, granulocyte macrophage colony-stimulating factor. Data are expressed as mean ± SD

In each group, 52.5% and 47.5% of patients were naïve HCV status and previously failed therapy respectively. The mean 25(OH)D level was 20.27 ± 4.83 ng/mL and 20.88 ± 5.40 ng/mL in placebo and vitamin D group respectively. Other parameters including HCV genotypes, HCV viral loads, transaminase levels and liver fibrosis scores were not significant between two groups (Table 1).

The mean IP-10 level in placebo group was slightly lower than the levels in vitamin D group (665.61 pg/mL vs 770.27 pg/mL) without statistical differences. While the mean DPP IV levels in placebo was slightly higher than the level in vitamin D group (6551.1 ng/mL vs 6137.7 npg/mL) without statistical differences. There were no significant differences in the baseline serum Th1/2 cytokines and DPP IV between two groups (Table 1).

### Changes of parameters at 6-week supplements

At the end of 6-week supplements, the median and range of serum 25(OH)D levels in the placebo and vitamin D groups were 21.22 (10.4–30.2) ng/mL, 45.93 (14.5–81.1) ng/mL, respectively. Vitamin D deficiency were corrected in all 40 patients who received vitamin D supplement. While vitamin D levels remained low and were not changed from the baseline levels in the placebo group (Table 2 and Fig 2).

**Table 2:** Comparing mean serum levels of each parameter between pre-and post-supplements in placebo and vitamin D groups.

Variables (Unit in pg/mL)	Placebo (40)			Vitamin D (40)		
	Pre: Mean (SE)	Post: Mean (SE)	P-value	Pre: Mean (SE)	Post: Mean (SE)	P-value
25(OH)D*	20.59 (0.76)	21.87 (0.84)	0.229	20.88 (0.85)	45.93 (2.43)	<0.001^A^
IP-10	665.61 (77.05)	748.88 (87.12)	0.130	770.27 (101.51)	636.47 (70.91)	0.036^B^
DPP IV*	6137.29 (250.50)	6408.33 (288.11)	0.386	6,551.06 (277.83)	6,032.37 (214.90)	0.028^C^
IL-2	23.07 (12.09)	28.69 (16.29)	0.318	8.20 (3.85)	4.49 (2.11)	0.120
IL-4	3.99 (0.79)	3.99 (0.96)	1.000	5.39 (0.98)	5.57 (1.12)	0.750
IL-5	4.69 (0.76)	4.47 (0.73)	0.717	7.60 (1.89)	8.06 (2.50)	0.623
IL-10	7.56 (3.00)	7.05 (2.66)	0.635	17.09 (7.04)	20.01 (8.82)	0.342
IL-12	29.25 (15.50)	26.80 (14.56)	0.670	40.61 (17.60)	50.11 (21.98)	0.292
IL-13	4.68 (1.27)	5.48 (1.52)	0.575	11.24 (3.57)	11.76 (4.95)	0.841
IFN-γ	251.64 (52.88)	237.28 (48.87)	0.594	328.31 (60.64)	304.90 (60.82)	0.429
TNF-α	602.45 (217.28)	548.33 (191.46)	0.884	651.52 (200.44)	752.94 (298.86)	0.487
GM-CSF	21.96 (9.01)	23.18 (7.63)	0.787	27.77 (6.91)	29.33 (9.92)	0.781

* unit in ng/mL;

^*A*^ 95% CI [20.65, 29.45];

^*B*^ 95% CI [-9.32, -258.28];

^*C*^ 95% CI [-60.42, -983.15]

**Fig 2:**
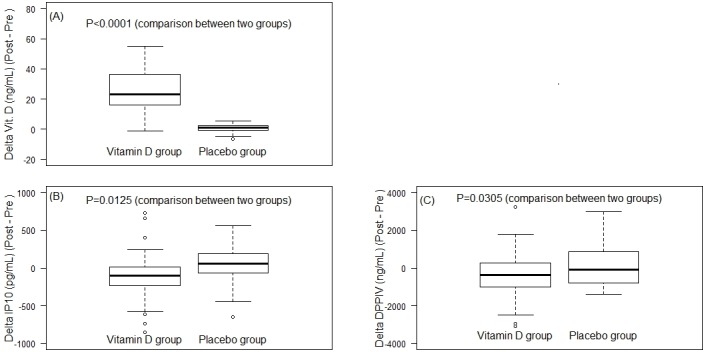
Mean changes of vitamin D levels. (A), IP-10 (B) and DPP IV (C) levels after 6-week supplements in placebo and vitamin D groups were demonstrated. The x-axis of each graph represents mean changes of the levels (delta). The blue and orange boxes represent placebo group and vitamin D group, respectively.

There were no differences of the transaminase levels at baseline and no changes after 6 weeks of supplements with placebo or vitamin D (data not shown). There were no significant differences of Th1 and Th2 cytokines levels in between group and no changes were observed during 6-week period of the study (Table 3).

**Table 3:** Comparing changes in serum levels (or delta) of each parameter in placebo and vitamin D groups during 6-week period of supplement.

Changes of serum or delta (Δ) levels (unit in pg/mL)	Placebo group	Vitamin D group	Estimate (SE)^B^	*P* values ^A^	Effect size ^C^
25(OH)D*	0.560	25.050	24.399 (2.233) ^D^	<.0001	2.47
IP-10	83.270	-133.800	-183.834 (71.852) ^E^	0.0125	0.59
DPP IV*	271.04	-518.69	-619.464 (281.070) ^F^	0.0305	0.55
IL-2	5.630	-3.710	-5.973 (5.399)	0.2720	0.35
IL-4	0.001	0.179	0.200 (0.780)	0.7983	0.08
IL-5	-0.211	0.467	0.171 (1.065)	0.8728	0.18
IL-10	-0.506	2.922	2.129 (3.092)	0.4931	0.22
IL-12	-2.451	9.495	11.598 (10.624)	0.2784	0.24
IL-13	0.797	0.520	-1.158 (2.967)	0.6974	0.00
IFN-γ	-16.119	1.014	115.927 (182.167)	0.5264	0.05
TNF-α	-14.351	-23.407	2.515 (38.146)	0.9476	0.08
GM-CSF	1.223	1.611	0.966 (7.282)	0.8949	0.02

* Unit in ng/mL;

^*A*^ Comparison between two groups was performed through ANCOVA. In ANCOVA, the dependent variable is the post-test measure, and the pre-test measure was a covariate and controlled for.

^*B*^ Estimate is the adjusted difference between treatment and placebo group (pre-test measure was adjusted for).

^*C*^ Cohen’s d effect size: 0.2 small, 0.5 medium, 0.8 large magnitude of effects

95% CI of estimate

^*D*^ [19.953, 28.844];

^*E*^ [-326.910, -40.758];

^*F*^ [-1179.15, -59.781]

After 6 weeks, the delta value of IP-10 level was decreased significantly in vitamin D group, as compared to the increased level in placebo group, -133.8 vs 83.27 pg/mL; 95% CI [326.910, -40.758], p = 0.0125 (Table 3 and Fig 2).

The mean serum DPP IV levels were not significant difference between pre-and post-supplements in both groups. During 6-week period, the mean DPP IV level in placebo group had trend to increase, and on the opposite direction, the mean level was decreased in vitamin D group. Comparing the mean changes (or delta changes) between the two groups showed significant differences, placebo group 255.46 ± 216.35 vs vitamin D group -521.79 ± 228.09; 95% CI [-1179.15, -59.781], p = 0.03 (Table 3 and Fig 2).

An effect size analysis (Cohen’s d) was used to a quantitative measure of the strength of the outcome. The result showed strong magnitude of vitamin d changes (2.47), and moderate effect of VD on the changes of IP-10 (0.59) and DPP IV (0.55) levels (Table 3). The results also suggested that this RCT was performed with reasonable number of cases.

We try to assess whether in this total population (80 CHC cases), serum levels of VD would affect the IP-10 levels in general. As shown in the scatter plot (Fig 3), even the dots are quite scattered, the best fit line could be plotted and the regression equation of [IP-10 = 841.34–4.45 x VD] was demonstrated. In other words, we could postulate that serum IP-10 decreases with increase in serum VD levels, and each unit of VD increase will lead to 4.5 unit decrease in serum IP-10.

**Fig 3:**
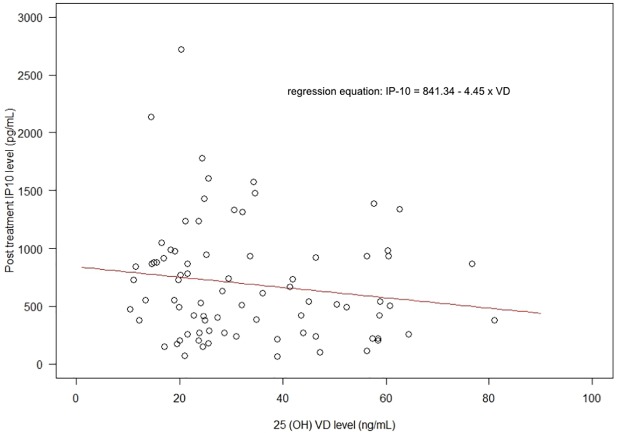
Scatter plot of post treatment VD levels (x-axis) and post treatment IP-10 level (y-axis) including fit line and regression equation were demonstrated.

## Discussion

HCV is one of the most common causes of chronic liver diseases, endstage liver disease and hepatocellular carcinoma worldwide [1, 2]. While vitamin D deficiency is also the most common nutritional deficiency in general population and in patients with chronic liver diseases [8]. When the two conditions come together, the patients experienced more severity of liver diseases [19, 20] and responded less to the treatment as compared to the one without vitamin D deficiency [21–24]. Upon correction of the deficiency, the treatment outcomes were improving [25]. Two serum markers, chemokine IP-10 and enzyme DPP IV (CD26), were identified as important markers predicting the outcome of CHC treatment [10, 14, 26]. The higher the level of the IP10 and the activity of the enzyme DPP IV predicted the poorer outcomes.

Our results suggested that vitamin D deficiency is a common factor connecting the dots of immunological derangements known to be the factors affecting the patients’ outcomes. Our finding demonstrated that upon correction of vitamin D deficiency in CHC patients within 6 weeks, both IP-10 and DPP IV levels were significantly reduced as compared to the placebo group. While, the serum levels of TH1 and TH2 related cytokines were remaining unchanged. In addition, the vitamin D replacement regimen used in this study for CHC patients were effective and could reversed the deficiency within a period of 6 weeks.

*In vitro* study demonstrated that Vitamin D acted directly on human hepatocytes as an innate immune response through interferon signaling pathway [27]. In CHC patients, there were increased in chemotactic cytokine expression including IP-10 on hepatocytes, especially around lobular area and periportal interface area [11]. This phenomenon resulted in recruitment of CXCR3-expressing T cells into the sites of infection [28]. The link between vitamin D and IP-10 was demonstrated, *in vitro*, in LPS-stimulated primary human monocytes, which the expression of IP-10 was suppressed by vitamin D [13]. In CHC patients treated with vitamin D/pegylated interferon/ribavirin showed significant reduction of serum IP-10 within 4 weeks of treatment and lower interferon stimulating gene (ISG) mRNA expression in hepatocytes as compare to the control arm without vitamin D [29]. The reduction of inflammatory responses, however, seemed paradox to explain the benefit on viral eradication. This phenomenon could also be seen in CHC liver pathology. The pathological finding of CHC patients who had better chance of responses to the treatment usually had lesser extent of lobular and interface hepatitis [11]. In addition, increase in ISG expression in CHC patients predicted poorer responses to the treatment [30, 31]. The finding of reduction of ISG expressions in the liver and serum IP-10 levels, however, were criticized as an adaptive immune stabilizing effect of vitamin D on the out of control immune responses in CHC patients [29]. Our data showed the same evidence of IP-10 reduction within 6 weeks, however, without influences of interferon. Whether, benefit of vitamin D on the treatment responses were added on by the effect of vitamin D alone, or as a synergistic effect of vitamin D on interferon are remaining unanswered.

High IP-10 levels were described in CHC patients, difficult to treated patients and at the end of therapy in non-responders [10, 32]. These evidences seemed to be paradox to explain the idea of chemotactic cytokines orchestrating antiviral responses. Previously, the soluble enzymes DPP IV (CD26) levels were found to be significant lower in CHC patients who had sustained virological responses (SVR) as compared to those without SVR [33, 34]. Recently, the explanation came into light as the antagonistic form of IP-10 was demonstrated in a high proportion of the total IP-10 levels in CHC patients [14]. The enzyme DPP IV cleaved two amino acids at the N-terminal creating the antagonistic form, resulting in a competitive blockade of the CXCR3 receptors and, consequently, preventing T-cells recruitment [35]. DPP IV enzymatic activity and levels were increased according to the levels of IP-10 in CHC patients and significantly reduced in the patients who responded to the treatment [14, 33].

Our result showed significant reduction of DPP IV enzyme activity by the effect of vitamin D alone without an impact of interferon. The effect of vitamin D on reduction of IP-10 in CHC patients might be the direct effect on the cells as described above [13]. Our second theory is the effect through IP-10/DPP IV cascade. Data suggested that vitamin D had an effect on compartmentalization of adaptive immune responses in regulation of a T cell lineage, TH17. Vitamin D suppressed IL-17 expression in mouse model of colitis and multiple sclerosis [36, 37]. Lack of vitamin D resulted in IL-17 elevation [38]. *In vitro* data suggested that HCV protein promoted dendritic cell differentiation into TH17 cells [39]. TH17 cells were shown to be a primary source and had high expression of DPP IV enzymes [40]. For these reasons, vitamin D deficiency in CHC patients might leads to TH17 upregulation, DDPIV enzyme overactivity and ultimately increase IP-10 antagonists. However, our further assessment on the serum levels of IL-17 in this clinical trial was not sensitive enough to show the changes (Data not shown). Proof of this Vitamin D/TH17/DPP IV/IP-10 antagonist cascade concept requires further investigation.

Previous data suggested that T cell responses in the CHC patients showed predominated TH1 lineage in liver tissues and TH-2 profiles in peripheral blood cells [41, 42]. Besides, vitamin D kept immunological balances by promoting cells differentiation into TH-2 cells [7]. Three-month supplement of vitamin D in active adults with or without vitamin D deficiency in a placebo-control trial showed no change in the levels of TH1 and TH2 cytokines [43]. Our study was unable to demonstrate differences of the TH1/TH2 cytokine profiles in CHC patients with vitamin D deficiency during pre and post vitamin D replacement. This may be a limitation of our study, as the changes were measured on serum cytokine profiles, not in the liver tissues. These results were the combinations of overall body immune responses and may not sensitive enough to represent changes in the liver tissues.

Currently, many countries around the world use DAAs as a new standard treatment for CHC which yield over ninety percent cure rate. However, in some countries, pegylated interferon plus ribavirin regimens as an old standard treatment, remain the only drugs available for CHC due to the high cost and availability of the DAAs. So far, there is no study addressing the important of vitamin D deficiency on the outcome of treatment by using the new pure oral DAAs or triple combination of PegIFN/RBV/DAA regimens. It might be the fact that the SVR rate of the latter regimens is closed to 100%. To improve the outcome of treatment by increasing body immune responses with vitamin D supplement may not be required.

For the new era of DAA regimens for CHC, the role of IP-10 and DDP-4 levels for the treatment response prediction may not be any more clinical usefulness. Nonetheless, this scientific finding might add on the understanding of immuno-pathophysiologic background of the CHC with vitamin D deficiency. In addition, minor proportion of CHC cases who resist to the DAA regimens, immunomodulator based therapy based on interferon or restore immune dysfunction in the patients with vitamin D deficiency might have a role for consideration.

In summary, our finding suggested that correction of vitamin D deficiency in CHC patients resulted in reduction of IP-10 levels and DPPP-IV activity. These reductions gave an additional data that might link or explain the benefit of vitamin D replacement in the treatment of CHC patients. However, the immunologic events happened in these complicated scenario of vitamin D deficiency and CHC infection required proper scientific investigations to explain our initial results on vitamin D deficiency/IP-10/DPP IV axis in CHC patients.

## Supporting information

S1 File CONSORT 2010 checklist. (PDF)

S2 File Protocol in Thai. (PDF)

S3 File Protocol in English. (PDF)

S4 File Supplement statistics. (PDF)

S5 File Data set. (PDF)

## Abbreviations

1, 25(OH)_2_D: 1–25 dihydroxyvitamin D
25(OH)D: 25-hydroxyvitamin D
ALT: alanine aminotransferase
AST: aspartate aminotransferase
BMI: body mass index
CD26: cluster of differentiation 26
CHC: chronic hepatitis C
CXCL-10: C-X-C chemokine 10
DPP IV: dipeptidyl peptidase-4
GM-CSF: granulocyte macrophage colony-stimulating factor
IFN-γ: interferon gamma
IL: interleukin
IP-10: interferon gamma-induced protein 10
PegIFN: pegylated interferon
SVR: sustained virological response
Th1: T helper cell type 1
Th2: T helper cell type 2
TNF-α: tumor necrosis factor alpha
