# Supplementary material for: Correction of vitamin D deficiency facilitated suppression of IP-10 and DPP IV levels in patients with chronic hepatitis C: A randomised double-blinded, placebo-control trial
Source: PLoS One. 2017 Apr 4;12(4):e0174608. doi: 10.1371/journal.pone.0174608 (PMC5380326; doi:10.1371/journal.pone.0174608)
Supplement: S2 File — (PDF) [file pone.0174608.s002.pdf]

Research proposal for IRB submission

Faculty of Medicine, Chulalongkorn University

Bangkok, Thailand

สารบัญ หน้า

แผ่นหน้า 02

1. Background 03

2. Hypothesis and research question 06

3. Research methodology 07

4. References 11

5. appendix 13

 5.1 Vitamin D dosage 13

 5.2 Case record form 14

 5.3 Patient information 18

 5.4 Informed consent sheet 25

Research proposal for IRB submission

Faculty of Medicine, Chulalongkorn University

Bangkok, Thailand

ชื่อเรื่อง (ภาษาไทย) การศึกษาผลของการให้วิตามินดี ที่มีผลต่อการตอบสนองด้านภูมิคุ้มกัน ในผู้ป่วยไวรัสตับอักเสบซีที่มีภาวะการขาดวิตามินดี

(ภาษาอังกฤษ) Effect of vitamin D replacement associated with adaptive immune response in chronic hepatitis C patients with vitamin D deficiency

ผู้วิจัยหลัก 1. นายแพทย์ เกรียงศักดิ์ เจริญสุข

2. นายแพทย์ ดร. ปิยะวัฒน์ โกมลมิศร์

หน่วยงาน สาขาวิชาโรคทางเดินอาหาร ภาควิชาอายุรศาสตร์

คณะแพทยศาสตร์ จุฬาลงกรณ์มหาวิทยาลัย

คำสำคัญ (key word) Vitamin D deficiency, Vitamin D replacement, Chronic hepatitis C, Immune response, Inducible protein-10 (IP-10)

1. **ความสำคัญและที่มาของปัญหาการวิจัย**

วิตามินดี มีความสำคัญต่อระบบอวัยวะต่างๆภายในร่างกาย เป็นวิตามินที่ละลายได้ในไขมันโดยทั่วไปคนปกติจะได้รับวิตามินดีเข้าสู่ร่างกาย 2 ทางด้วยกันคือ ได้รับจากอาหาร อาหารเสริม อาหารที่มีวิตามินดีมากได้แก่ น้ำมันตับปลา ไขมัน นม เนย ตับสัตว์ ปลาทู ไข่แดง และแสงแดดอุลตร้าไวโอเล็ต (คลื่นความถี่ 290-315 นาโนเมตร) ซึ่งจะ ผ่านผิวหนังที่ และเปลี่ยนไปเป็น active form ในเวลาต่อมา กระบวนการเมตาบอลิซึมที่สำคัญของวิตามินดี เกิดขึ้นที่ ตับและไตได้เป็น 25-hydroxy vitamin D และ 1,25 dihydroxy vitamin D ตามลำดับ โดยผ่านการควบคุมจากพาราไทรอยด์ฮอร์โมน ระดับของแคลเซียมและฟอสฟอรัส [1, 2]

มีการศึกษาจำนวนมากที่แสดงให้เห็นถึงฤทธิ์และประสิทธิภาพของวิตามินดีในการป้องกันและรักษาโรคต่าง ๆ นอกจากโรคกระดูกและข้อ ได้แก่ โรคหัวใจ multiple sclerosis, โรคเบาหวานชนิดที่ 1, โรคระบบภูมิคุ้มกันและโรคมะเร็งเป็นต้น **[3-5]** ตลอดจนการเปลี่ยนแปลงของภูมิคุ้มกัน ทั้งชนิด Innate และ Adaptive immune response **[6, 7]** กล่าวคือ วิตามินดีจะลดการทำงานของ T helper-1 cells โดยทำให้ปริมาณ IL-2, IL-12 **และ** IFNγ1 ลดลง และเพิ่มการทำงานของ T helper-2 cells โดยทำให้ปริมาณ IL-4, TGF βเพิ่มขึ้น **[7-12]**

โดยทั่วไป ระดับภาวะการขาดวิตามินดี ยังไม่ได้มีข้อสรุปที่แน่ชัด โดยความเห็นส่วนใหญ่ยอมรับที่ระดับของ 25-hydroxy vitamin D ที่ต่ำกว่า 30 นาโนกรัม/เดซิลิตร ขณะที่ภาวะวิตามินดีเป็นพิษจะอยู่ที่ระดับที่สูงมากกว่า 150 นาโนกรัม/เดซิลิตร จากนิยามดังกล่าวจะมีประชากรบางส่วนที่มี ภาวะการขาดวิตามินดี โดยที่ไม่มีอาการ และอาจสูงถึง 40-100 เปอร์เซ็นต์ ของประชากรชาย และหญิงกลุ่มสูงอายุ ในบางรายงาน**[5]** โดยประชากรกลุ่มดังกล่าว ตรวจพบภาวการณ์ขาดวิตามินดีได้ โดยไม่มีอาการผิดปกติต่อร่างกาย โดยทั่วไปแบ่งระดับการขาดวิตามินดีไว้ดังนี้

<u>25-hydroxy vitamin D</u>		
	> 30 ng/mls.	อยู่ในระดับปกติ
	20-30 ng/ml.	ภาวะการขาดวิตามินดีระดับเล็กน้อย
	10-20 ng/ml.	ภาวะการขาดวิตามินดีระดับปานกลาง
	< 10 ng/ml.	ภาวการณ์ขาดวิตามินดีระดับรุนแรง

ตับเป็นอวัยวะที่มีบทบาทสำคัญเมตาบอลิซึมของวิตามินดี วิตามินดีจากผิวหนังและอาหารจะถูก hydroxylate ที่ตับได้เป็น 25-hydroxy vitamin D (25(OH)D) ก่อนที่จะส่งไปที่ไตเพื่อเปลี่ยนเป็น active form ในช่วงระยะเวลา 3-5 ปีที่ผ่านมามีรายงาน ภาวะการขาดวิตามินดีในผู้ป่วยโรคตับเรื้อรังต่างๆมากขึ้น ทั้งในกลุ่ม Cholestatis และ Noncholestatis เช่น ผู้ป่วยโรคตับแข็ง ผู้ป่วย Primary biliary cirrhosis เป็นต้น ทำให้เพิ่มความเสี่ยงต่อการเกิดภาวะกระดูกพรุนและกระดูกหักเพิ่มขึ้น Fisher และคณะได้รายงานผู้ป่ยจำนวน 100 คนในกลุ่มผู้ป่วยโรคตับเรื้อรังทั้งในกลุ่ม Noncholestatis พบว่ามีระดับ 25(OH)D ที่ต่ำกว่า

20 นาโนกรัม/มิลลิลิตร 86**%** ในกลุ่มที่มีตับแข็ง เทียบกับกลุ่มที่ไม่มีตับแข็ง 49% (P < 0.001) โดยระดับดังกล่าวสัมพันธ์กับระดับค่าการแข็งตัวของเลือด และระดับความรุนแรงของโรคตับของผู้ป่วย **[12-14]**

ไวรัสตับอักเสบซี เป็นสาเหตุหนึ่งที่สำคัญต่อการเกิดไวรัสตับอักเสบเรื้อรัง อันนำไปสู่การเกิดถาวะตับแข็ง และมะเร็งตับตามมา ตัวไวรัสเป็น RNA virus ในกลุ่ม Flaviviridae คาดประมาณว่ามีประชากาาร 2.7 ล้านคนที่มีการติดไวรัสตับอักเสบซี หลังการติดเชื้อ ร่างกายจะมีการตอบสนองต่อไวรัสทั้งแบบจำเพาะและแบบไม่จำเพาะเจาะจง การตอบสนองแบบไม่จำเพาะเจาะจง เกิดขึ้นผ่าน Interferon α/β ที่สร้างจากเซลล์ตับที่ติดเชื้อและ Plasmacytoid dendritic cells (DC) ซึ่งมีบทบาทในการยับยั้งการเพิ่มจำนวนของไวรัสตับอักเสบซี**,** กระตุ้น expression ของ HLA class I บน Antigen Presenting Cell (APC) ตลอดจนกระตุ้น NK cells และ Cytotoxic T cell **[7, 8, 11]**

ไวรัสจะกระตุ้น Innate และ Adaptive Immune response ของ host โดยมี Macrophage**,** Natural killer cell และ Neutrophil ในการกระตุ้น innate immune response ในการสร้าง Inflammatory cytokines และมี Activated Dendritic cells ในการกระตุ้น adaptive immune response ให้มี CD4+ CD8+ T-cells ในการกำจัดไวรัสออกจากร่างกาย กล่าวโดยสรุปคือ ภาวะภูมิคุ้มกันของร่างกายมีผลต่อการตอบสนองไวรัสตับอักเสบ **[9, 15]**

การศึกษาในเวลาต่อมาพบว่าระดับของวิตามินดีก่อนการรักษา มีความสัมพันธ์กับการตอบสนองต่อการรักษา **(**Sustained virological response; SVR**)** ในผู้ป่วยไวรัสตับอักเสบซี ซึ่งเป็นผลมาจากการผลของวิตามินดีที่มีผลต่อการเปลี่ยนแปลงการตอบสนองด้านภูมิต้านทาน กล่าวคือกลุ่มที่มีระดับวิตามินดีปกติ มีระดับการสนองต่อการรักษา ที่สูงกว่ากลุ่มที่ขาดวิตามินดี รายงานล่าสุดพบว่าการให้วิตามินดีเสริมในระหว่างการรักษาไวรัสตับอักเสบซี สามารถเพิ่มการสนองต่อการรักษาได้ **[16, 17]**

Inducible protein-10 (IP-10) หรือ CXCL10 เป็นสารตัวหนึ่งในกลุ่ม CXC chemokine ซึ่งมีหน้าที่กระตุ้น chemotactic function ในเซลล์ชนิดต่างๆในระบบภูมิคุ้มกันผ่านทาง CXCR3 receptor **[18, 19]** มีการศึกษาพบว่ามีการ express CXCR3ในกลุ่มผู้ป่วยไวรัสตับอักเสบซีเรื้อรัง และตรวจพบ IP-10 mRNAและโปรตีนในตับของผู้ป่วยปริมาณมากซึ่งสัมพั นธ์ต่อต่อระดับซีรั่ม IP-10 จึงเชื่อว่าระดับการเปลี่ยนแปลงของ IP-10 มีบทบาทที่สำคัญต่อการติดเชื้อไวรัสตับอักเสบซี มีหลายการศึกษาที่พบว่าระดับของซีรั่ม IP-10 เป็น predictor ที่มีผลต่อการตอบสนองการรักษาในผู้ป่วยไวรัสตับอักเสบซีเรื้อรัง กล่าวคือระดับของซีรั่ม IP-10 ที่สูงมีผลต่อการตอบสนองที่ไม่ดีในผู้ป่วยที่ได้รับการรักษาด้วย Peginterferon-Ribavirin **[20-22]**

การศึกษาของ Kuo,Y.T. และคณะ พบว่าวิตามินดีมีผลต่อการเปลี่ยนแปลง tumor necrosis factor-alpha และ chemokines ต่างๆรวมถึงระดับ IP-10 โดยพบว่าการให้วิตามินดีมีผลทำให้ระดับของ IP-10 ลดลง **[23]**

จาก back ground ที่กล่าวข้างต้น จะเห็นได้ว่าวิตามินดีมีบทบาทที่สำคัญมากมายในด้านต่างๆ การ การศึกษาวิจัยชิ้นนี้ จึงเกิดขึ้นเพื่อทดสอบว่าวิตามินดีจะสัมพันธ์ต่อการเปลี่ยนแปลงระดับ cytokine และ IP-10 ในระบบภูมิคุ้มกัน ในผู้ป่วยไวรัสตับอักเสบซีหรือไม่ อันจะก่อให้เกิดองค์ความรู้ใหม่ๆ ซึ่งจะมีผลต่อการรักษาผู้ป่วยไวรัสตับอักเสบซีต่อไปในอนาคต

2. **คำถามงานวิจัย และสมมุติฐาน**

2.1 คำถามการวิจัย

2.1.1คำถามหลัก **(Primary research question):** การให้ยาวิตามินดีในผู้ป่วยไวรัสตับอักเสบซี ที่มีภาวะการขาดวิตามินดี สามารถเพิ่มการตอบสนองทางด้านภูมิคุ้มกัน (Adaptive Immune response) ได้หรือไม่

2.1.2 คำถามรอง **(Secondary research question):** การให้ยาวิตามินดีในผู้ป่วยไวรัสตับอักเสบซี ที่มีภาวะการขาดวิตามินดี มีความสัมพันธ์กับการเปลี่ยนแปลงของสาร Inducible protein-10 (IP-10) และ cytokine หรือ เอนไซม์อื่น ๆ ได้หรือไม่

2.2 วัตถุประสงค์การวิจัย **(Objective)**

1. เพื่อศึกษาผลของการให้ยาวิตามินดี ที่มีผลต่อการตอบสนองด้านภูมิคุ้มกัน ในผู้ป่วยไวรัสตับอักเสบซีที่มีภาวะการขาดวิตามินดี

2. เพื่อศึกษาความสัมพันธ์ของการให้วิตามินดี ที่มีผลต่อการเปลี่ยนแปลงของ สาร Inducible protein-10 (IP-10) และ cytokine หรือ เอนไซม์อื่น ๆ

2.3 สมมุติฐาน **(Hypothesis)**

การให้ยาวิตามินดี ในผู้ป่วยไวรัสตับอักเสบซี ที่มีภาวะการขาดวิตามินดี ทำให้การตอบสนองด้านภูมิคุ้มกัน **(Adaptive immune response)** ของผู้ป่วยดีขึ้น

2.4 คำสำคัญ

Vitamin D deficiency, Vitamin D replacement, Chronic hepatitis C, Immune response, Inducible protein-10 (IP-10)

2.5 ผลประโยชน์ที่คาดว่าจะได้รับจากการวิจัย (Expected Benefits and Application)

ทำให้ทราบถึงผลของการให้วิตามินดี ที่อาจมีผลต่อการตอบสนองทางด้านการรักษา ผู้ป่วยที่เป็นโรคตับอักเสบซีเรื้อรัง ทำให้แพทย์ผู้ที่ดูแลและให้การรักษาผู้ป่วยโรคนี่ สามารถนำความรู้ที่ได้จากการวิจัยชิ้นนี้ มาประยุกต์ใช้ในการรักษาและแนะนำผู้ป่วยได้อย่างถูกต้องและเหมาะสมต่อไป

ทำให้ทราบและเข้าใจ ความสัมพันธ์ระหว่างวิตามินดี**,** สาร Inducible protein-10 (IP-10) และการเปลี่ยนแปลงระดับภูมิคุ้มกัน ในระดับ cytokine ในผู้ป่วยที่เป็นโรคตับอักเสบซีเรื้อรังอันจะนำไปประยุกต์ใช้ในทางคลีนิกในด้านการรักษาผู้ป่วยไวรัสตับอักเสบต่อไปในอนาคต

3 **รูปแบบการวิจัย**

3.1 รูปแบบการวิจัย

เป็นการวิจัยเชิงทดลองทางคลีนิค Randomized, double – blind, placebo – controlled trial

3.2 population

3.2.1 ประชากรเป้าหมาย: ผู้ที่เป็นโรคไวรัสตับอักเสบซีเรื้อรัง ที่ติดตามการรักษา ในโรงพยาบาลจุฬาลงกรณ์ ระหว่างเดือนมกราคม 2555 ถึง ธันวาคม 2555 และยินดีเข้าร่วมโครงการศึกษาวิจัยเพื่อเจาะเลือดและรับการรักษา หลังจากได้อธิบายรายละเอียดของโครงการ โดยจะเริ่มเก็บข้อมูลหลังจากได้รับการรับรองจากคณะกรรมการจริยธรรมการวิจัย

3.2.2 เกณฑ์ในการคัดเลือกผู้เข้าทำการศึกษา

1. คนไทยมีอายุระหว่าง 18-65 ปีทั้งหญิงและชาย
2. ผู้ป่วย Compensated Liver Cirrhosis

   Albumin > 3.4 mg/dl Total bilirubin <1.5 mg/dl

   INR < 1.2 Platelet > 100,000
3. ไม่มีประวัติโรคไต ค่าการทำงานของไตอยู่ในเกณฑ์ปกติ Creatinine < 1.5 mg/dl.
4. ยินดีเข้าร่วมโครงการศึกษาวิจัยเพื่อเจาะเลือดและรับการรักษา

3.2.3 เกณฑ์ในการคัดออก

1. ผู้ป่วย Decompensated Liver Cirrhosis
2. ผู้ป่วยที่มีโรคเอดส์ ตับอักเสบมีเรื้อรัง ร่วมด้วย
3. หญิงตั้งครรภ์และให้นมบุตร
4. ผู้ป่วยที่เป็นโรค Autoimmune disease
5. ผู้ป่วยที่กำลังมีภาวะการติดเชื้อไวรัสหรือแบคทีเรีย (Active viral and bacterial infection)
6. ผู้ป่วยที่มีประวัติใช้ยา Steroid หรือ Immunosuppression
7. ผู้ป่วยที่ไม่สามารถเซ็นใบยินยอมเข้าร่วมการศึกษาวิจัย

3.3 การคำนวณขนาดตัวอย่าง

เนื่องจากเป็นการทดลองที่ยังไม่เคยมีมาก่อน ดังนั้นการคำนวณประชากรที่ใช้ในการศึกษานี้ จึงไม่สามารถทำได้ ทางผู้วิจัยจึงวางแผนแนวทางการแก้ไข โดยให้การศึกษานี้เป็นการศึกษานำร่อง หลังจากนั้นจะนำค่าที่ได้เพื่อมาคำนวณตามสูตร เพื่อหาขนาดตัวอย่างที่แท้จริงอีกครั้ง โดยกำหนดจำนวนตัวอย่างผู้ป่วยใน

การศึกษาวิจัยครั้งนี้ประมาณ 80 คน เนื่องจากชุดการตรวจ cytokine สามารถตรวจได้ 80 ชุด/set อีกทั้งเป็นค่าที่ได้ มาจากการประมาณของผู้เชี่ยวชาญ

3.4 การวิเคราะห์ข้อมูลและสถิติ

- ข้อมูลพื้นฐาน แสดงค่า mean±SD สำหรับข้อมูลเชิงปริมาณ และ percent สำหรับข้อมูลเชิงคุณภาพ
- การเปรียบเทียบข้อมูลทั้งข้อมูลพื้นฐาน และความแตกต่างของระดับ immune function ของ T helper -1 และ 2, IP-10 ที่ก่อนการรักษา ให้วิตามินดี และสิ้นสุดการรักษาที่สัปดาห์สุดท้าย ใช้สถิติ Paired T-test
- การเปรียบเทียบข้อมูลทั้งข้อมูลพื้นฐาน และความแตกต่างของระดับ immune function ของ T helper -1 และ 2, IP-10 ระหว่าง placebo และ treatment group ใช้สถิติ Analysis of Co-variance (ANCOVA model)

3.5 การสังเกต และการวัด

1) วัดระดับวิตามินดี โดยการตรวจวัดระดับวิตามินดี 25 Hydroxyvitamin D (25 (OH)Vitamin D) ในเลือดของผู้ป่วยไวรัสตับอักเสบซีทุกคนก่อนการรักษา และหลังให้การรักษาด้วยวิตามินดีที่สัปดาห์ที่ 4 และ 6
2) การวัดการตอบสนองทางภูมิคุ้มกัน (Immune Response) ทำโดยใช้วิธีการวัดระดับของ Cytokine ที่เกี่ยวข้องกับการทำงานของ Type 1และ2 T helper cells (Th1 และ Th2 ตามลำดับ) โดยใช้ชุดการตรวจน้ำยา. Bio-Plex Th1/Th2 Panel. ของ BioRad ประกอบด้วย IL-2, 4, 5,10,12,13, GM-CSF, TNF alpha, IFN gamma วัดระดับดังกล่าวก่อนและหลังการรักษา
3) การวัดระดับสาร Inducible Protein-10 ทำโดยการวัด baseline IP-10 ก่อนการรักษา ใช้commercial set : Quantikine human CXCL10/IP-10 immunoassay(R&D Systems) ใช้ซีรั่มเจือจาง 1:2 ในการตรวจ โดยค่าการวัดของ IP- 10 ที่ได้จะอยู่ระหว่าง 8-500 pg/mL ในกรณีที่ค่าสูงกว่า1,000 pg/mL จะใช้ซีรั่มเจือจางที่ 1:5 วัดระดับดังกล่าวก่อนและหลังการรักษา โดยการตรวจ cytokine และ IP-10 จะทำการตรวจที่ห้องปฏิบัติการทางภูมิคุ้มกันวิทยา ตึก อปร ชั้น 18

3.6 การดำเนินการวิจัย

1. ตรวจคัดกรองผู้ป่วยที่ได้รับการวินิจฉัยว่าเป็นโรคไวรัสตับอักเสบซีจากการตรวจเลือด โดยการซักประวัติตรวจร่างกายทั่วไป ซักประวัติเกี่ยวกับปัจจัยเสี่ยง ร่วมกับขออนุญาตดูประวัติการรักษาในเรื่องของการวินิจฉัย และยาที่ได้รับประจำ

2. ผู้ที่ผ่านการตรวจคัดกรองเบื้องต้น จะได้รับการซักประวัติและเก็บข้อมูลตามแบบการเก็บข้อมูลผู้ป่วยที่เข้าร่วมโครงการ (Case record form) ทั้งผู้ป่วยไวรัสตับอักเสบซีที่เคยและไม่เคยรับการรักษาด้วยยาต้านไวรัสสูตรมาตรฐาน (Naïve & previously treatment cases)

3. ผู้ป่วยจะได้รับการเจาะเลือดเพื่อตรวจระดับวิตามินดี (25 OH Vitamin D) ในเลือด ในกรณีที่ระดับต่ำกว่า 30 ng / ml ผู้ป่วยจะได้รับการแนะนำ inform consent เพื่อเข้าโครงการวิจัย เจาะเลือดปริมาณ 10 มิลลิลิตร ใส่ในหลอด Clotted blood และ EDTA อย่างละ 5 มิลลิลิตร เก็บแช่เย็น เพื่อรอส่งตรวจวัดระดับ cytokine ของ T-helper-1 และ 2, ระดับ Inducible-10 (IP-10) ก่อนเริ่มการให้การรักษา

4. ทำการสุ่มตัวอย่างผู้ป่วยแบบแบ่งชั้น (Stratified random sampling) แบบ 1:1 โดยแบ่งกลุ่มผู้ป่วยไวรัสตับอักเสบซีที่เคยและไม่เคยรับการรักษาด้วยยาต้านไวรัสสูตรมาตรฐาน (Naïve & previously treatment cases) เข้าในแต่ละ group โดยให้มีสัดส่วนเท่า ๆ กัน กลุ่มหนึ่งได้รับยาหลอก (Placebo group) และอีกกลุ่มได้รับยาวิตามินดี (Vitamin D replacement group) โดยให้จำนวน และขนาดตาม protocol ที่แนบไว้ที่ภาคผนวก โดยผู้ทำการวิจัยไม่ทราบว่าเป็นยาจริงหรือยาหลอก

5. หลังให้การรักษานาน 6 สัปดาห์ผู้ป่วยจะได้รับการตรวจ เจาะเลือด เพื่อตรวจระดับวิตามินดี (25 OH Vitamin D) ในเลือด และเจาะเลือดปริมาณ 10 มิลลิลิตร ใส่ในหลอด Clotted blood และ EDTA อย่างละ 5 มิลลิลิตร เก็บแช่เย็น เพื่อรอส่งตรวจวัดระดับ cytokine ของ T-helper-1 และ 2, ระดับ Inducible-10 (IP-10) อีกครั้ง หลังสิ้นสุดการรักษา

การตรวจวัดระดับของ cytokine ของ T-helper-1 และ 2, ระดับ Inducible-10 (IP-10) ทำการตรวจจท ห้องปฏิบัติการทางภูมิคุ้มกันวิทยา ตึก อปร ชั้น 18

***** ผู้ป่วยทุกรายที่ศึกษาวิจัย หลังสิ้นสุดการศึกษา หากยังตรวจพบว่ามีภาวะวิตามินในเลือดต่ำ จะได้รับการรักษา โดยการให้วิตามินดีฟรี ต่อไปจนกว่า ระดับผลเลือดจะอยู่ในเกณฑ์ปกติ *****

6. นำผลที่ได้ไปวัดค่าทางสถิติต่อไป

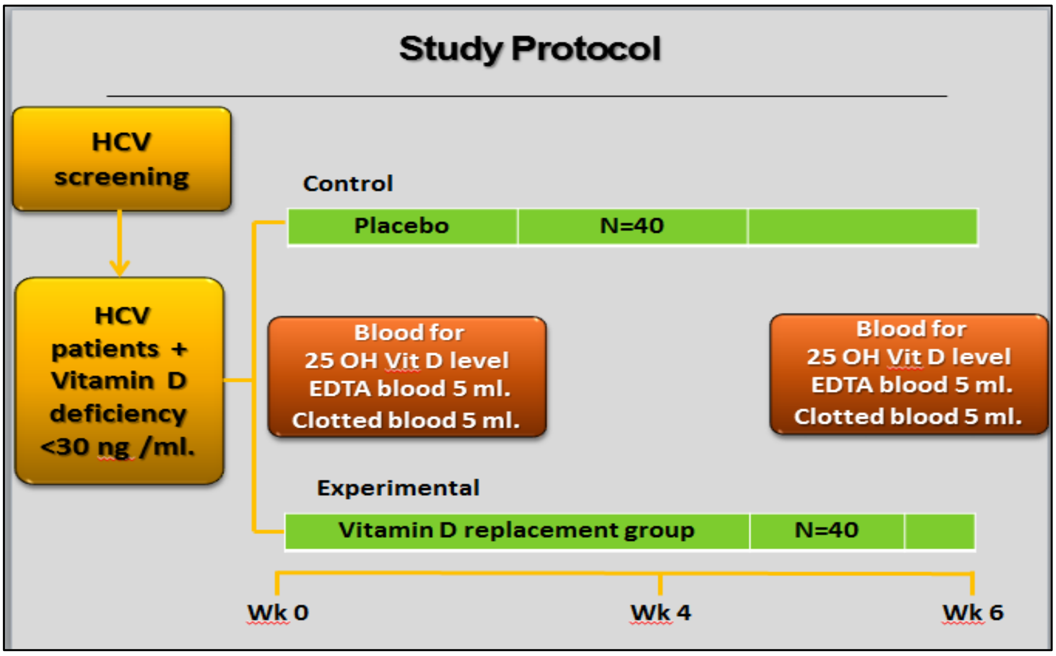

13) การบริหารการวิจัยและตารางการปฏิบัติงาน (Administration and time schedule)

Administration	**2554**		**2555**					**2556**					**2557**				
	**8**	**12**	**2**	**4**	**6**	**8**	**12**	**2**	**4**	**6**	**8**	**12**	**2**	**4**	**6**	**8**	**12**
1.Preparation	←	—	→														
2. Perform researches				←	—	—	—	—	→								
3. Initial analysis										←	—	→					
4. Additional parameters Measurement (DPP-4, Th-17 and others)													←	—	→		
5. Final analysis																←	→

3.7 งบประมาณ (Budget) สำหรับผู้ป่วยวิจัยประมาณ 80 คน

**รายการ**	**ค่าใช้จ่าย**
ค่าตรวจระดับวิตามินดี 25 OH vitamin D level<br><br>1,200 บาท * 3 * 80 คน	288,000 บาท
ค่าชุดตรวจระดับ cytokines (Th1/Th2) 80 kits/set<br><br>75,000 บาท/set * 2 ชุด	150,000 บาท
ค่าชุดตรวจระดับ Inducible protein – 10 (IP-10) 80 kits/set<br><br>21,000 บาท/set * 2 ชุด	42,000 บาท
ค่ายาวิตามินดี (Vitamin D2)	3,000 บาท
ค่าเครื่องมือ อุปกรณ์ tube ใส่เลือด	5,000 บาท
ค่าจัดทำยาหลอก (Placebo)	5,000 บาท
**รวมจำนวนเงินทั้งสิ้น**	500,000 บาท

4. เอกสารอ้างอิง

1. Holick CN, Stanford JL, Kwon EM, Ostrander EA, Nejentsev S, Peters U. Comprehensive association analysis of the vitamin D pathway genes, VDR, CYP27B1, and CYP24A1, in prostate cancer. Cancer epidemiology, biomarkers & prevention : a publication of the American Association for Cancer Research, cosponsored by the American Society of Preventive Oncology. 2007;16(10):1990-9. Epub 2007/10/13. doi: 10.1158/1055-9965.epi-07-0487. PubMed PMID: 17932346.

2. Holick MF. Vitamin D deficiency. The New England journal of medicine. 2007;357(3):266-81. Epub 2007/07/20. doi: 10.1056/NEJMra070553. PubMed PMID: 17634462.

3. Ardizzone S, Cassinotti A, Bevilacqua M, Clerici M, Porro GB. Vitamin D and inflammatory bowel disease. Vitamins and hormones. 2011;86:367-77. Epub 2011/03/23. doi: 10.1016/b978-0-12-386960-9.00016-2. PubMed PMID: 21419280.

4. Deeb KK, Trump DL, Johnson CS. Vitamin D signalling pathways in cancer: potential for anticancer therapeutics. Nature reviews Cancer. 2007;7(9):684-700. Epub 2007/08/28. doi: 10.1038/nrc2196. PubMed PMID: 17721433.

5. Lange CM, Bojunga J, Ramos-Lopez E, von Wagner M, Hassler A, Vermehren J, et al. Vitamin D deficiency and a CYP27B1-1260 promoter polymorphism are associated with chronic hepatitis C and poor response to interferon-alfa based therapy. Journal of hepatology. 2011;54(5):887-93. Epub 2010/12/15. doi: 10.1016/j.jhep.2010.08.036. PubMed PMID: 21145801.

6. Baeke F, Takiishi T, Korf H, Gysemans C, Mathieu C. Vitamin D: modulator of the immune system. Current opinion in pharmacology. 2010;10(4):482-96. Epub 2010/04/30. doi: 10.1016/j.coph.2010.04.001. PubMed PMID: 20427238.

7. Bikle DD. Vitamin D and the immune system: role in protection against bacterial infection. Current opinion in nephrology and hypertension. 2008;17(4):348-52. Epub 2008/07/29. doi: 10.1097/MNH.0b013e3282ff64a3. PubMed PMID: 18660668.

8. Bikle DD. Vitamin D regulation of immune function. Vitamins and hormones. 2011;86:1-21. Epub 2011/03/23. doi: 10.1016/b978-0-12-386960-9.00001-0. PubMed PMID: 21419265.

9. Chambers ES, Hawrylowicz CM. The impact of vitamin D on regulatory T cells. Current allergy and asthma reports. 2011;11(1):29-36. Epub 2010/11/26. doi: 10.1007/s11882-010-0161-8. PubMed PMID: 21104171.

10. Hewison M. Vitamin D and the immune system: new perspectives on an old theme. Endocrinology and metabolism clinics of North America. 2010;39(2):365-79, table of contents. Epub 2010/06/01. doi: 10.1016/j.ecl.2010.02.010. PubMed PMID: 20511058; PubMed Central PMCID: PMCPmc2879394.

11. Lange NE, Litonjua A, Hawrylowicz CM, Weiss S. Vitamin D, the immune system and asthma. Expert review of clinical immunology. 2009;5(6):693-702. Epub 2010/02/18. doi: 10.1586/eci.09.53. PubMed PMID: 20161622; PubMed Central PMCID: PMCPMC2812815.

12. Maruotti N, Cantatore FP. Vitamin D and the immune system. The Journal of rheumatology. 2010;37(3):491-5. Epub 2010/01/19. doi: 10.3899/jrheum.090797. PubMed PMID: 20080911.

13. Chailurkit LO, Kruavit A, Rajatanavin R. Vitamin D status and bone health in healthy Thai elderly women. Nutrition (Burbank, Los Angeles County, Calif). 2011;27(2):160-4. Epub 2010/04/16. doi: 10.1016/j.nut.2009.12.001. PubMed PMID: 20392596.

14. Fisher L, Fisher A. Vitamin D and parathyroid hormone in outpatients with noncholestatic chronic liver disease. Clinical gastroenterology and hepatology : the official clinical practice journal of the American Gastroenterological Association. 2007;5(4):513-20. Epub 2007/01/16. doi: 10.1016/j.cgh.2006.10.015. PubMed PMID: 17222588.

15. Bitetto D, Fattovich G, Fabris C, Ceriani E, Falleti E, Fornasiere E, et al. Complementary role of vitamin D deficiency and the interleukin-28B rs12979860 C/T polymorphism in predicting antiviral response in chronic hepatitis C. Hepatology (Baltimore, Md). 2011;53(4):1118-26. Epub 2011/04/12. doi: 10.1002/hep.24201. PubMed PMID: 21480318.

16. Kim TY. Role of vitamin D in chronic hepatitis C.

17. Lagging M, Askarieh G, Negro F, Bibert S, Soderholm J, Westin J, et al. Response prediction in chronic hepatitis C by assessment of IP-10 and IL28B-related single nucleotide polymorphisms. PloS one. 2011;6(2):e17232. Epub 2011/03/11. doi: 10.1371/journal.pone.0017232. PubMed PMID: 21390311; PubMed Central PMCID: PMCPMC3044738.
18. Luster AD. Chemokines--chemotactic cytokines that mediate inflammation. The New England journal of medicine. 1998;338(7):436-45. Epub 1998/02/12. doi: 10.1056/nejm199802123380706. PubMed PMID: 9459648.
19. Murdoch C, Finn A. Chemokine receptors and their role in inflammation and infectious diseases. Blood. 2000;95(10):3032-43. Epub 2000/05/16. PubMed PMID: 10807766.
20. Diago M, Castellano G, Garcia-Samaniego J, Perez C, Fernandez I, Romero M, et al. Association of pretreatment serum interferon gamma inducible protein 10 levels with sustained virological response to peginterferon plus ribavirin therapy in genotype 1 infected patients with chronic hepatitis C. Gut. 2006;55(3):374-9. Epub 2005/09/10. doi: 10.1136/gut.2005.074062. PubMed PMID: 16150856; PubMed Central PMCID: PMCPMC1856069.
21. Reiberger T, Aberle JH, Kundi M, Kohrgruber N, Rieger A, Gangl A, et al. IP-10 correlates with hepatitis C viral load, hepatic inflammation and fibrosis and predicts hepatitis C virus relapse or non-response in HIV-HCV coinfection. Antiviral therapy. 2008;13(8):969-76. Epub 2009/02/07. PubMed PMID: 19195322.
22. Zeremski M, Markatou M, Brown QB, Dorante G, Cunningham-Rundles S, Talal AH. Interferon gamma-inducible protein 10: a predictive marker of successful treatment response in hepatitis C virus/HIV-coinfected patients. Journal of acquired immune deficiency syndromes (1999). 2007;45(3):262-8. Epub 2007/04/07. doi: 10.1097/QAI.0b013e3180559219. PubMed PMID: 17414926.
23. Kuo YT, Kuo CH, Lam KP, Chu YT, Wang WL, Huang CH, et al. Effects of vitamin D3 on expression of tumor necrosis factor-alpha and chemokines by monocytes. Journal of food science. 2010;75(6):H200-4. Epub 2010/08/21. doi: 10.1111/j.1750-3841.2010.01704.x. PubMed PMID: 20722932.

5. Appendix

5.1 Protocol สำหรับการให้วิตามินดีในระดับค่าวิตามินที่แตกต่างกัน

Diagnosis	Vitamin D level (ng /mL)	Replacement Total dose (IU) / week	Ergocalciferol (D2) 20,000 IU/ tab (Take with meal)	Treatment duration
Optimal	> 30	-	-	-
Mild deficiency (insufficiency)	20 – 30	60,000	2 tab Monday and 1 tab Friday	6 weeks
Moderate deficiency	10 – 20	80,000	2 tab Monday and 2 tab Friday	6 weeks
Severe deficiency	< 10	100,000	3 tab Monday and 2 tab Friday	6 weeks

5.2 Case Record Form

<table>
<tr><td colspan="3">Effect of vitamin D replacement on adaptive immune response in chronic hepatitis C patients with vitamin D deficiency

Investigators : Kriangsak Charoensuk , MD. , Piyawat Komolmit, MD,PhD.</td><td colspan="2">Case number</td></tr>
<tr><td colspan="3"></td><td>Year</td><td></td></tr>
<tr><td></td><td colspan="2">Age (years)</td><td colspan="2">Sex .... Male
.... Female</td></tr>
<tr><td rowspan="3"><u>Baseline Characteristics</u>

Weight .... kgs.

Height .... cms.

BMI .... kgs./m^2

Date..

<u>Liver Biopsy</u>

Metavir score ....

Ishak Score..</td><td colspan="2">Date of start treatment (DD/MM/YYYY)</td><td colspan="2"></td></tr>
<tr><td colspan="4">Baseline 25(OH)Vitamin D level (ng/dl.)</td></tr>
<tr><td colspan="4">☐ < 10 ng/dl.
☐ 10 - 20 ng/dl.
☐ 20 – 30 ng/dl.
☐ > 30 ng/dl.

After treatment Vitamin D level ....ng/dl.</td></tr>
<tr><td rowspan="4">Underlying disease
☐ No ☐ Yes specify
____
____
____
Current medications
☐ No ☐ Yes specify
____
____
____
HCV diagnosis:
Duration of HCV diagnosis_____________months
HCV risk factor(s)
• Heterosexual
• Homosexual
• IVDU
• Blood transfusion
• Tattoo
• Unknown
• Other please define________________</td><td colspan="4">Baseline HCV genotype and VL prior treatment</td></tr>
<tr><td>Date of exam

(DD/MM/YYYY)</td><td>HCV genotype</td><td colspan="2">VL (copies/ml)</td></tr>
<tr><td></td><td></td><td colspan="2"></td></tr>
<tr><td></td><td></td><td colspan="2"></td></tr>
<tr><td colspan="5">History of previous treatment</td></tr>
<tr><td colspan="5">Previous HCV treatment ☐ Yes ☐ No</td></tr>
</table>

Regimen	Start date	Stop date	VL	Note

Treatment and Follow up Period

Regimens	Date of Follow up	HCV VL	Note	Important Lab

Laboratory Finding

Date ________________________

Complete Blood Count

Hct________Hb________ MCV ____________

Wbc __________________

%PMN _____________ ANC ______________

%Lymph ______________

%Mono_________ % Eosinophil _____________ Platelet Count __________

PT _______________

PTT _______________

INR _______________

VitaminD 25(OH)D

Baseline ______________________ ng/dl.

After treatment _______________ ng/dl.

Inducible protein-10 (IP-10) level __________________________________

Blood Chemistry

BUN_______Cr_______

Electrolyte

Na ____________ K ____________

Cl _____________ CO2 __________

Ca ____________ Phosphate ___________

Liver Function Test

TB ____________ DB ____________

Albumin _______ Globulin _______

SGOT __________ SGPT __________

ALP ____________

Immune function (Cytokine)

T Helper-1 ______________________________

T Helper-2 ______________________________

____

Serology profile

Anti HCV ______________ HBsAg ____________ Anti- HBsAg ____________

Anti HIV ______________

Lab Follow up

Date __________________

Hct ______Hb_____ MCV _____ Wbc ________%PMN _______%Lymph _______ ANC ________

Platelet Count _____________PT ______PTT _______INR __________

BUN_______Cr_______ TB_____ DB _____Albumin _______ Globulin _______

SGOT __________ SGPT __________ALP ___________

HCV Viral loads ___________________

Date __________________

Hct ______Hb_____ MCV _____ Wbc ________%PMN _______%Lymph _______ ANC ________

Platelet Count _____________PT ______PTT _______INR __________

BUN_______Cr_______ TB_____ DB _____Albumin _______ Globulin _______

SGOT __________ SGPT __________ALP ___________

HCV Viral loads __________________

Treatment

___OPD ___IPD ward___________

O Vitamin D2 dose _______________ IU/wk Duration_________weeks

O Vitamin D3 dose _______________ Duration_________weeks

O Pegylated interferon 2a dose________µg/kg/day Duration_________weeks

O Pegylated interferon 2b dose________µg/kg/day Duration_________weeks

O Ribavirin dose _________________ mgs. /day Duration_________weeks

O Adjusted dose drugs O No O Yes specify __________________________________

Date of dose adjustment ____________________ Reason _________________________

____

Record by _______________________

Date ____________________________

Lab Summary Record Form

Lab Summary Record form

Effect of vitamin D replacement on adaptive immune response in chronic hepatitis C patients with vitamin D deficiency

Investigators : Kriangsak Charoensuk , MD. , Piyawat Komolmit, MD, PhD.

Week 1: O EDTA blood ≥5 mls.
O Clotted bloods ≥ 5 mls.
O 25 (OH) vitamin D level

Week 4 O 25 (OH) vitaminD

Week 6 O EDTA blood ≥5 mls.
O Clotted bloods ≥ 5 mls.
O 25 (OH) vitamin D level

HN	Name	Vitamin D level Wks 1	Vitamin D Replacement Regimen	Vitamin D level Wks 4	Vitamin D level Wks 6	IP-10
		Date		Date	Date	
		Date		Date	Date	
		Date		Date	Date	
		Date		Date	Date	

5.3 ข้อมูลสำหรับผู้เข้าร่วมโครงการวิจัย

ชื่อโครงการวิจัย การศึกษาผลของการให้วิตามินดี ที่มีผลต่อการตอบสนองด้านภูมิคุ้มกันในผู้ป่วยไวรัสตับอักเสบซีที่มีภาวะการขาดวิตามินดี

<u>แพทย์ผู้ทำวิจัย</u>

ชื่อ .....นพ.เกรียงศักดิ์ เจริญสุข...

ที่อยู่ .....หน่วยทางเดินอาหาร ตึกพร้อมพันธ์ชั้น 1 รพ.จุฬาลงกรณ์ ถ.พระราม4 แขวง คลองเตย เขต ปทุมวัน กรุงเทพ 10330 ....

เบอร์โทรศัพท์ ......02-2564000..ต่อ 4356 กด 2, 081-8697003... (ที่ทำงานและมือถือ)................

ชื่อ ....ผศ.นพ.ดร.ปิยะวัฒน์ โกมลมิศร์...

ที่อยู่ ....หน่วยทางเดินอาหาร ตึกพร้อมพันธ์ชั้น 1 รพ.จุฬาลงกรณ์ ถ.พระราม4 แขวง คลองเตย เขต ปทุมวัน กรุงเทพ 10330..

เบอร์โทรศัพท์ .... 02-2564000..ต่อ 4356 กด 19...(ที่ทำงานและมือถือ).....................................

เรียน ผู้เข้าร่วมโครงการวิจัยทุกท่าน

ท่านได้รับเชิญให้เข้าร่วมในโครงการวิจัยนี้เนื่องจากท่านเป็นโรคไวรัสตับอักเสบซีเรื้อรัง ที่ตรวจพบว่ามีภาวการณ์ขาดวิตามินดี ก่อนที่ท่านจะตัดสินใจเข้าร่วมในการศึกษาวิจัยดังกล่าว ขอให้ท่านอ่านเอกสารฉบับนี้อย่างถี่ถ้วน เพื่อให้ท่านได้ทราบถึงเหตุผลและรายละเอียดของการศึกษาวิจัยในครั้งนี้ หากท่านมีข้อสงสัยใดๆ เพิ่มเติม กรุณาซักถามจากทีมงานของแพทย์ผู้ทำวิจัย หรือแพทย์ผู้ร่วมทำวิจัยซึ่งจะเป็นผู้สามารถตอบคำถามและให้ความกระจ่างแก่ท่านได้

ท่านสามารถขอคำแนะนำในการเข้าร่วมโครงการวิจัยนี้จากครอบครัว เพื่อน หรือแพทย์ประจำตัวของท่านได้ ท่านมีเวลาอย่างเพียงพอในการตัดสินใจโดยอิสระ ถ้าท่านตัดสินใจแล้วว่าจะเข้าร่วมในโครงการวิจัยนี้ ขอให้ท่านลงนามในเอกสารแสดงความยินยอมของโครงการวิจัยนี้

<u>เหตุผลความเป็นมา</u>

วิตามินดี มีความสำคัญต่อระบบอวัยวะต่างๆภายในร่างกาย กระบวนการเมตาบอลิซึมที่สาคัญของวิตามินดี เกิดขึ้นที่ ตับและไต วิตามินดีจากผิวหนังและอาหารจะถูกเปลี่ยนที่ตับ ก่อนที่จะส่งไปที่ไตเพื่อเปลี่ยนเป็นสารที่ออกฤทธิ์ ควบคุมระดับของแคลเซียมและฟอสฟอรัส โดยผ่านการควบคุมจากพาราไทรอยด์ฮอร์โมน

มีการศึกษาจานวนมากที่แสดงให้เห็นถึงฤทธิ์และประสิทธิภาพของวิตามินดีในการป้องกันและรักษาโรคต่าง ๆ นอกจากโรคกระดูกและข้อ ได้แก่ โรคหัวใจ โรคเบาหวานชนิดที่ 1 โรคระบบภูมิคุ้มกัน และโรคมะเร็ง เป็นต้น ตลอดจนการเปลี่ยนแปลงของภูมิคุ้มกัน กล่าวคือ วิตามินดีจะลดการทางานของ T helper-1 cells โดยทำให้ปริมาณ IL-2, IL-12 และ IFN alpha ลดลงและเพิ่มการทางานของ T helper-2 cells โดยทำให้ปริมาณ IL-4, TGF beta เพิ่มขึ้น

ในช่วงระยะเวลา 3-5 ปีที่ผ่านมามีรายงาน ภาวะการขาดวิตามินดีในผู้ป่วยโรคตับเรื้อรังกลุ่มต่างๆ มากขึ้น ทำให้เพิ่มความเสี่ยงต่อการเกิดภาวะกระดูกพรุนและกระดูกหักเพิ่มขึ้น อีกทั้งการศึกษาในเวลาต่อมาพบว่าระดับของวิตามินดีก่อนการรักษา มีความสัมพันธ์กับการตอบสนองต่อการรักษา ในผู้ป่วยไวรัสตับอักเสบซี ซึ่งเป็นผลมาจากการผลของวิตามินดีที่มีผลต่อการเปลี่ยนแปลงการตอบสนองด้านภูมิต้านทาน กล่าวคือกลุ่มที่มีระดับวิตามินดีปกติ จะมี การตอบสนองต่อการรักษา ที่สูงกว่ากลุ่มที่ขาดวิตามินดี รายงานล่าสุดพบว่าการให้วิตามินดีเสริมในระหว่างการรักษาไวรัสตับอักเสบซี สามารถเพิ่ม การตอบสนองต่อการรักษาได้มากขึ้น

Inducible protein-10 (IP-10) เป็นสารตัวหนึ่งในร่างกาย ซึ่งมีหน้าที่กระตุ้น ในเซลล์ชนิดต่างๆในระบบภูมิคุ้มกันผ่านทางตัวรับ มีการศึกษาพบว่ามีการเพิ่มขึ้นของตัวรับดังกล่าวในกลุ่มผู้ป่วยไวรัสตับอักเสบซีเรื้อรัง และตรวจพบ IP-10 mRNAและโปรตีนในตับของผู้ป่วยปริมาณมาก ซึ่งสัมพันธ์ต่อต่อระดับซีรั่ม IP-10 จึงเชื่อว่าระดับการเปลี่ยนแปลงของ IP-10 มีบทบาทที่สาคัญต่อการติดเชื้อไวรัสตับอักเสบซี มีหลายการศึกษาที่พบว่าระดับของซีรั่ม IP-10 เป็นสารที่มีผลต่อการตอบสนองการรักษาในผู้ป่วยไวรัสตับอักเสบซีเรื้อรัง กล่าวคือระดับของซีรั่ม IP-10 ที่สูงมีผลต่อการตอบสนองที่ไม่ดีในผู้ป่วยที่ได้รับการรักษาด้วย ยาสูตรมาตรฐาน

มีการศึกษาพบว่าวิตามินดีมีผลต่อการเปลี่ยนแปลงสารต่างๆในระบบภูมิคุ้มกัน รวมถึงระดับ IP-10 โดยพบว่าการให้วิตามินดี มีผลทำให้ระดับของ IP-10ลดลง

จากที่กล่าวข้างต้นจะเห็นได้ว่าวิตามินดีมีบทบาทที่สำคัญมากมายในด้านต่าง ๆ การศึกษาวิจัยชิ้นนี้ จึงเกิดขึ้นเพื่อทดสอบว่าวิตามินดีจะสัมพันธ์ต่อการเปลี่ยนแปลงระดับไซโตไคน์ cytokineและ IP-10 ในระบบภูมิคุ้ม กัน ในผู้ป่วยไวรัสตับอักเสบซีหรือไม่ อันจะก่อให้เกิดองค์ความรู้ใหม่ๆ ซึ่งจะมีผลต่อแนวทาการรักษาผู้ป่วยไวรัสตับอักเสบซีต่อไปในอนาคต

<u>วัตถุประสงค์ของการศึกษา</u>

วัตถุประสงค์หลักจากการศึกษาในครั้งนี้คือ

1. เพื่อศึกษาผลของการให้ยาวิตามินดี ที่มีผลต่อการตอบสนองด้านภูมิคุ้มกัน ในผู้ป่วยไวรัสตับอักเสบซีที่มีภาวะการขาดวิตามินดี

2. เพื่อศึกษาความสัมพันธ์ของการให้วิตามินดี ที่มีผลต่อการเปลี่ยนแปลงของ สาร Inducible protein-10 (IP-10) และไซโตไคน์ หรือ เอนไซม์เฉพาะอื่น ๆ

จำนวนผู้เข้าร่วมในโครงการวิจัย คือ 80 คน

วิธีการที่เกี่ยวข้องกับการวิจัย

หลังจากท่านให้ความยินยอมที่จะเข้าร่วมในโครงการวิจัยนี้ ผู้วิจัยจะขอตรวจเลือด โดยการเจาะเลือด จำนวน 10 ซีซี (ช้อนชา) เพื่อตรวจระดับวิตามินดีในเลือด และเก็บเลือดเพื่อวัดระดับสารไซโตไคน์ (Cytokines) เพื่อคัดกรองว่าท่านมีคุณสมบัติที่เหมาะสมที่จะเข้าร่วมในการวิจัย

หากท่านมีคุณสมบัติตามเกณฑ์คัดเข้า ท่านจะได้รับเชิญให้มาพบแพทย์ตามวันเวลาที่ผู้ทำวิจัยนัดหมาย เพื่อตรวจร่างกายอย่างละเอียดและ ประเมินผลทางห้องปฏิบัติการต่าง ๆและรับยา โดยตลอดระยะเวลาที่ท่านอยู่ในโครงการวิจัย คือ 6 สัปดาห์ และมาพบผู้วิจัยหรือผู้ร่วมทำวิจัยทั้งสิ้น 2 ครั้ง

ความรับผิดชอบของอาสาสมัครผู้เข้าร่วมในโครงการวิจัย

เพื่อให้งานวิจัยนี้ประสบความสำเร็จ ผู้ทำวิจัยใคร่ขอความความร่วมมือจากท่าน โดยจะขอให้ท่านปฏิบัติตามคำแนะนำของผู้ทำวิจัยอย่างเคร่งครัด รวมทั้งแจ้งอาการผิดปกติต่าง ๆ ที่เกิดขึ้นกับท่านระหว่างที่ท่านเข้าร่วมในโครงการวิจัยให้ผู้ทำวิจัยได้รับทราบ

เพื่อความปลอดภัย ท่านไม่ควรใช้วัคซีน หรือรับประทานยาอื่น จากการจ่ายยาโดยแพทย์อื่นหรือซื้อยาจากร้านขายยา ขอให้ท่านปรึกษาผู้ทำวิจัย ทั้งนี้เนื่องจากวัคซีน หรือยาดังกล่าวอาจมีผลต่อยา วิตามินดี ที่ท่านได้รับจากผู้ทำวิจัย ดังนั้นขอให้ท่านแจ้งผู้ทำวิจัยเกี่ยวกับยาที่ท่านได้รับในระหว่างที่ท่านอยู่ในโครงการวิจัย

ความเสี่ยงที่อาจได้รับ

ความเสี่ยงจากการรับประทานยาทุกชนิดอาจทำให้เกิดอาการไม่พึงประสงค์ได้ทั้งสิ้นไม่มากก็น้อย แพทย์ผู้ทำการวิจัยขอชี้แจงถึงความเสี่ยงและความไม่สบายที่อาจสัมพันธ์กับยาที่ศึกษาทั้งหมดดังนี้

มีข้อมูลที่แสดงว่ายาวิตามินดี อาจมีผลข้างเคียงในกรณีที่ได้ขนาดที่สูงมากกว่าปกติมาก ได้แก่ อาการอ่อนเพลีย ปวดศีรษะ เบื่ออาหาร ปากแห้ง คลื่นไส้อาเจียน รวมถึงอาการข้างเคียงและความไม่สบายที่ยังไม่มีการรายงานด้วย ขนาดยาที่ใช้ในการรักษาในการศึกษานี้เป็นขนาดมาตรฐานที่ใช้กันโดยทั่วไป โอกาสการเกิดผลข้างเคียงดังกล่าวจึงเกิดได้น้อย อย่างไรก็ตามระหว่างที่ท่านอยู่ในโครงการวิจัยจะมีการติดตามดูแลสุขภาพของท่านอย่างใกล้ชิด

กรุณาแจ้งผู้ทำวิจัยในกรณีที่พบอาการดังกล่าวข้างต้น หรืออาการอื่น ๆ ที่พบร่วมด้วย ระหว่างที่อยู่ในโครงการวิจัย ถ้ามีการเปลี่ยนแปลงเกี่ยวกับสุขภาพของท่าน ขอให้ท่านรายงานให้ผู้ทำวิจัยทราบโดยเร็ว

ความเสี่ยงที่ได้รับจากการเจาะเลือด

ท่านมีโอกาสที่จะเกิดอาการเจ็บ เลือดออก ช้ำจากการเจาะเลือด อาการบวมบริเวณที่เจาะเลือด หรือหน้ามืด และโอกาสที่จะเกิดการติดเชื้อบริเวณที่เจาะเลือดพบได้น้อยมาก

ความเสี่ยงที่ไม่ทราบแน่นอน

ท่านอาจเกิดอาการข้างเคียง หรือความไม่สบาย นอกเหนือจากที่ได้แสดงในเอกสารฉบับนี้ ซึ่งอาการข้างเคียงเหล่านี้เป็นอาการที่ไม่เคยพบมาก่อน เพื่อความปลอดภัยของท่าน ควรแจ้งผู้ทำวิจัยให้ทราบทันทีเมื่อเกิดความผิดปกติใดๆ เกิดขึ้น

หากท่านมีข้อสงสัยใดๆ เกี่ยวกับความเสี่ยงที่อาจได้รับจากการเข้าร่วมในโครงการวิจัย ท่านสามารถสอบถามจากผู้ทำวิจัยได้ตลอดเวลา

หากมีการค้นพบข้อมูลใหม่ ๆ ที่อาจมีผลต่อความปลอดภัยของท่านในระหว่างที่ท่านเข้าร่วมในโครงการวิจัย ผู้ทำวิจัยจะแจ้งให้ท่านทราบทันที เพื่อให้ท่านตัดสินใจว่าจะอยู่ในโครงการวิจัยต่อไปหรือจะขอถอนตัวออกจากการวิจัย

การพบแพทย์นอกตารางนัดหมายในกรณีที่เกิดอาการข้างเคียง

หากมีอาการข้างเคียงใด ๆ เกิดขึ้นกับท่าน ขอให้ท่านรีบมาพบแพทย์ที่สถานพยาบาลทันที ถึงแม้ว่าจะอยู่นอกตารางการนัดหมาย เพื่อแพทย์จะได้ประเมินอาการข้างเคียงของท่าน และให้การรักษาที่เหมาะสมทันที หากอาการดังกล่าวเป็นผลจากการเข้าร่วมในโครงการวิจัย ท่านจะไม่เสียค่าใช้จ่าย

ประโยชน์ที่อาจได้รับ

การเข้าร่วมในโครงการวิจัยนี้อาจจะทำให้ท่านมีสุขภาพที่ดีขึ้น หรืออาจจะลดความรุนแรงของโรคได้ แต่ไม่ได้รับรองว่าสุขภาพของท่านจะต้องดีขึ้นหรือความรุนแรงของโรคจะลดลงอย่างแน่นอน

วิธีการและรูปแบบการรักษาอื่น ๆ ซึ่งมีอยู่สำหรับอาสาสมัคร

ท่านไม่จำเป็นต้องเข้าร่วมโครงการวิจัยนี้เพื่อประโยชน์ในการรักษาโรคที่ท่านเป็นอยู่ เนื่องจากมีแนวทางการรักษาอื่น ๆ หลายแบบสำหรับรักษาโรคของท่านได้ ดังนั้นจึงควรปรึกษาแนวทางการรักษาวิธีอื่นๆ กับแพทย์ผู้ให้การรักษาท่านก่อนตัดสินใจเข้าร่วมในการวิจัย

ข้อปฏิบัติของท่านขณะที่ร่วมในโครงการวิจัย

ขอให้ท่านปฏิบัติดังนี้

- ขอให้ท่านให้ข้อมูลทางการแพทย์ของท่านทั้งในอดีต และปัจจุบัน แก่ผู้ทำวิจัยด้วยความสัตย์จริง

- ขอให้ท่านแจ้งให้ผู้ทำวิจัยทราบความผิดปกติที่เกิดขึ้นระหว่างที่ท่านร่วมในโครงการวิจัย
- ขอให้ท่านงดการใช้ยาอื่นนอกเหนือจากที่ผู้ทำวิจัยได้จัดให้ รวมถึงการรักษาอื่น ๆ เช่น การรักษาด้วยสมุนไพร การซื้อยาจากร้านขายยา
- ขอให้ท่านแจ้งให้ผู้ทำวิจัยทราบทันที หากท่านได้รับยาอื่นนอกเหนือจากยาที่ใช้ในการศึกษาตลอดระยะเวลาที่ท่านอยู่ในโครงการวิจัย
- ขอให้ท่านนำยาที่ใช้ในการศึกษาของท่านทั้งหมดที่เหลือจากการรับประทานมาให้ผู้ทำวิจัยทุกครั้งที่นัดหมายให้มาพบ

<u>อันตรายที่อาจเกิดขึ้นจากการเข้าร่วมในโครงการวิจัยและความรับผิดชอบของผู้ทำวิจัย/ผู้สนับสนุนการวิจัย</u>

หากพบอันตรายที่เกิดขึ้นจากการวิจัย ท่านจะได้รับการรักษาอย่างเหมาะสมทันที และท่านปฏิบัติตามคำแนะนำของทีมผู้ทำวิจัยแล้ว ผู้ทำวิจัย/ผู้สนับสนุนการวิจัยยินดีจะรับผิดชอบค่าใช้จ่ายในการรักษาพยาบาลของท่าน และการลงนามในเอกสารให้ความยินยอม ไม่ได้หมายความว่าท่านได้สละสิทธิ์ทางกฎหมายตามปกติที่ท่านพึงมี

ในกรณีที่ท่านได้รับอันตรายใด ๆ หรือต้องการข้อมูลเพิ่มเติมที่เกี่ยวข้องกับโครงการวิจัย ท่านสามารถ

ติดต่อกับผู้ทำวิจัยคือ นพ.เกรียงศักดิ์ เจริญสุข เบอร์โทรศัพท์มือถือ 081-8697003 ได้ตลอด 24 ชั่วโมง

<u>ค่าใช้จ่ายของท่านในการเข้าร่วมการวิจัย</u>

ท่านจะได้รับยาวิตามินดี ในโครงการวิจัยจากผู้สนับสนุนการวิจัยโดยไม่ต้องเสียค่าใช้จ่าย

ค่าใช้จ่ายอื่นที่เกี่ยวข้องกับโครงการวิจัย เช่น ค่าธรรมเนียมทางการแพทย์ และ ค่าวิเคราะห์ทางห้องปฏิบัติการ ผู้สนับสนุนการวิจัยจะเป็นผู้รับผิดชอบทั้งหมด รวมทั้งค่าเดินทางตามที่ท่านได้มาพบแพทย์ตามนัดทุกครั้ง ครั้งละ 250 บาท รวมทั้งหมด 2 ครั้ง

<u>การเข้าร่วมและการสิ้นสุดการเข้าร่วมโครงการวิจัย</u>

การเข้าร่วมในโครงการวิจัยครั้งนี้เป็นไปโดยความสมัครใจ หากท่านไม่สมัครใจจะเข้าร่วมการศึกษาแล้ว ท่านสามารถถอนตัวได้ตลอดเวลา การขอถอนตัวออกจากโครงการวิจัยจะไม่มีผลต่อการดูแลรักษาโรคของท่านแต่อย่างใด

ผู้ทำวิจัยอาจถอนท่านออกจากการเข้าร่วมการวิจัย เพื่อเหตุผลด้านความปลอดภัยของท่าน หรือเมื่อผู้สนับสนุนการวิจัยยุติการดำเนินงานวิจัย หรือ ในกรณีดังต่อไปนี้

- ท่านไม่สามารถปฏิบัติตามคำแนะนำของผู้ทำวิจัย
- ท่านรับประทานยาที่ไม่อนุญาตให้ใช้ในการศึกษา
- ท่านตั้งครรภ์ระหว่างที่เข้าร่วมโครงการวิจัย

- ท่านเกิดอาการข้างเคียง หรือความผิดปกติของผลทางห้องปฏิบัติการจากการได้รับยาที่ใช้ในการศึกษา
- ท่านแพ้ยาที่ใช้ในการศึกษา
- ท่านต้องการปรับเปลี่ยนการรักษาด้วยยาตัวที่ไม่ได้รับอนุญาตจากการวิจัยครั้งนี้

การปกป้องรักษาข้อมูลความลับของอาสาสมัคร

ข้อมูลที่อาจนำไปสู่การเปิดเผยตัวท่าน จะได้รับการปกปิดและจะไม่เปิดเผยแก่สาธารณชน ในกรณีที่ผลการวิจัยได้รับการตีพิมพ์ ชื่อและที่อยู่ของท่านจะต้องได้รับการปกปิดอยู่เสมอ โดยจะใช้เฉพาะรหัสประจำโครงการวิจัยของท่าน

จากการลงนามยินยอมของท่านผู้ทำวิจัย และผู้สนับสนุนการวิจัยสามารถเข้าไปตรวจสอบบันทึกข้อมูลทางการแพทย์ของท่านได้แม้จะสิ้นสุดโครงการวิจัยแล้วก็ตาม หากท่านต้องการยกเลิกการให้สิทธิ์ดังกล่าว ท่านสามารถแจ้ง หรือเขียนบันทึกขอยกเลิกการให้คำยินยอม โดยส่งไปที่ นพ.เกรียงศักดิ์ เจริญสุข หน่วยทางเดินอาหาร ตึกพร้อมพันธ์ชั้น 1 รพ.จุฬาลงกรณ์ ถ.พระราม4 แขวง คลองเตย เขต ปทุมวัน กรุงเทพ 10330

หากท่านขอยกเลิกการให้คำยินยอมหลังจากที่ท่านได้เข้าร่วมโครงการวิจัยแล้ว ข้อมูลส่วนตัวของท่านจะไม่ถูกบันทึกเพิ่มเติม อย่างไรก็ตามข้อมูลอื่น ๆ ของท่านอาจถูกนำมาใช้เพื่อประเมินผลการวิจัย และท่านจะไม่สามารถกลับมาเข้าร่วมในโครงการนี้ได้อีก ทั้งนี้เนื่องจากข้อมูลของท่านที่จำเป็นสำหรับใช้เพื่อการวิจัยไม่ได้ถูกบันทึก

จากการลงนามยินยอมของท่านแพทย์ผู้ทำวิจัยสามารถบอกรายละเอียดของท่านที่เกี่ยวกับการเข้าร่วมโครงการวิจัยนี้ให้แก่แพทย์ผู้รักษาท่านได้

สิทธิ์ของผู้เข้าร่วมในโครงการวิจัย

ในฐานะที่ท่านเป็นผู้เข้าร่วมในโครงการวิจัย ท่านจะมีสิทธิ์ดังต่อไปนี้

1. ท่านจะได้รับทราบถึงลักษณะและวัตถุประสงค์ของการวิจัยในครั้งนี้
2. ท่านจะได้รับการอธิบายเกี่ยวกับระเบียบวิธีการของการวิจัยทางการแพทย์ รวมทั้งยาและอุปกรณ์ที่ใช้ในการวิจัยครั้งนี้
3. ท่านจะได้รับการอธิบายถึงความเสี่ยงและความไม่สบายที่จะได้รับจากการวิจัย
4. ท่านจะได้รับการอธิบายถึงประโยชน์ที่ท่านอาจจะได้รับจากการวิจัย
5. ท่านจะได้รับการเปิดเผยถึงทางเลือกในการรักษาด้วยวิธีอื่น ยา หรืออุปกรณ์ซึ่งมีผลดีต่อท่านรวมทั้งประโยชน์และความเสี่ยงที่ท่านอาจได้รับ
6. ท่านจะได้รับทราบแนวทางในการรักษา ในกรณีที่พบโรคแทรกซ้อนภายหลังการเข้าร่วมในโครงการวิจัย
7. ท่านจะมีโอกาสได้ซักถามเกี่ยวกับงานวิจัยหรือขั้นตอนที่เกี่ยวข้องกับงานวิจัย

8. ท่านจะได้รับทราบว่าการยินยอมเข้าร่วมในโครงการวิจัยนี้ ท่านสามารถขอถอนตัวจากโครงการเมื่อไรก็ได้ โดยผู้เข้าร่วมในโครงการวิจัยสามารถขอถอนตัวจากโครงการโดยไม่ได้รับผลกระทบใด ๆ ทั้งสิ้น
9. ท่านจะได้รับสำเนาเอกสารใบยินยอมที่มีทั้งลายเซ็นและวันที่
10. ท่านมีสิทธิ์ในการตัดสินใจว่าจะเข้าร่วมในโครงการวิจัยหรือไม่ก็ได้ โดยปราศจากการใช้อิทธิพลบังคับข่มขู่ หรือการหลอกลวง

หากท่านไม่ได้รับการชดเชยอันควรต่อการบาดเจ็บหรือเจ็บป่วยที่เกิดขึ้นโดยตรงจากการวิจัย หรือท่านไม่ได้รับการปฏิบัติตามที่ปรากฏในเอกสารข้อมูลคำอธิบายสำหรับผู้เข้าร่วมในการวิจัย ท่านสามารถร้องเรียนได้ที่ คณะกรรมการจริยธรรมการวิจัย คณะแพทยศาสตร์ จุฬาลงกรณ์มหาวิทยาลัย ตึกอานันทมหิดลชั้น 3 โรงพยาบาลจุฬาลงกรณ์ ถนนพระราม 4 ปทุมวัน กรุงเทพฯ 10330 โทร 0-2256-4455 ต่อ 14, 15 ในเวลาราชการ

ขอขอบคุณในการร่วมมือของท่านมา ณ ที่นี้

5.4 ใบยินยอมรับการรักษาและเข้าร่วมโครงการวิจัย

ชื่อโครงการ การศึกษาเปรียบเทียบผลของการขาดวิตามินดี ที่มีผลต่อการตอบสนองด้านภูมิคุ้มกันและการรักษาในผู้ป่วยไวรัสตับอักเสบซี

วันให้คำยินยอม วันที่....................เดือน...พ.ศ. ....

ข้าพเจ้านาย /นาง/นางสาว...อายุ ....ปี

ที่อยู่ปัจจุบัน

....

....เบอร์โทรศัพท์...

ได้อ่านรายละเอียดจากเอกสารข้อมูลสำหรับผู้เข้าร่วมโครงการวิจัยที่แนบมาและข้าพเจ้ายินยอมเข้าร่วมโครงการวิจัยโดยสมัครใจ

ข้าพเจ้าได้รับสำเนาเอกสารแสดงความยินยอมเข้าร่วมในโครงการวิจัยที่ข้าพเจ้าได้ลงนามและวันที่ พร้อมด้วยเอกสารข้อมูลสำหรับผู้เข้าร่วมโครงการวิจัย ทั้งนี้ก่อนที่จะลงนามในใบยินยอมให้ทำการวิจัยนี้ ข้าพเจ้าได้รับการอธิบายจากผู้วิจัยถึงวัตถุประสงค์ของการวิจัย ระยะเวลาของการทำวิจัย วิธีการวิจัย อันตรายหรืออาการที่อาจเกิดขึ้นจากการวิจัย รวมทั้งประโยชน์ที่จะเกิดจากการวิจัย และแนวทางการรักษาโดยวิธีอื่นอย่างละเอียด ข้าพเจ้ามีเวลาและโอกาสเพียงพอในการซักถามข้อสงสัยจนมีความเข้าใจเป็นอย่างดีแล้ว โดยผู้วิจัยได้ตอบคำถามต่างๆด้วยความเต็มใจไม่ปิดบังซ่อนเร้นจนข้าพเจ้าพอใจ

ข้าพเจ้ามีสิทธิที่จะบอกเลิกเข้าร่วมโครงการวิจัยเมื่อใดก็ได้ โดยไม่จำเป็นต้องแจ้งเหตุผล และการบอกเลิกการเข้าร่วมการวิจัยนี้จะไม่มีผลต่อการรักษาโรคหรือสิทธิอื่นๆ ที่ข้าพเจ้าจะพึงได้รับต่อไป

ผู้วิจัยรับรองว่าจะเก็บข้อมูลส่วนตัวของข้าพเจ้าเป็นความลับ และจะเปิดเผยได้เฉพาะเมื่อได้รับการยินยอมจากข้าพเจ้าเท่านั้น บุคคลอื่นในนามของบริษัทผู้สนับสนุนการวิจัย คณะกรรมการพิจารณาจริยธรรมการวิจัยหรือผู้ได้รับอำนาจมอบหมายให้เจ้ามาตรวจและประมวลข้อมูลของผู้เข้าร่วมการวิจัย ทั้งนี้จะต้องกระทำไปเพื่อวัตถุประสงค์เพื่อตรวจสอบความถูกต้องของข้อมูลเท่านั้น โดยการตกลงที่จะเข้าร่วมการศึกษานี้ข้าพเจ้าได้ให้คำยินยอมที่จะให้มีการตรวจสอบข้อมูลประวัติทางการแพทย์ของผู้เข้าร่วมวิจัยได้

ผู้วิจัยรับรองว่าจะไม่มีการเก็บข้อมูลใดๆ ของผู้เข้าร่วมวิจัยเพิ่มเติม หลังจากที่ข้าพเจ้าขอยกเลิกการเข้าร่วมโครงการวิจัยและต้องการให้ทำลายเอกสารและ/หรือตัวอย่างที่ใช้ตรวจสอบทั้งหมดที่สามารถสืบค้นถึงตัวข้าพเจ้าได้ ข้าพเจ้าเข้าใจว่า ข้าพเจ้ามีสิทธิที่จะตรวจสอบหรือแก้ไขข้อมูลส่วนตัวของข้าพเจ้าและสามารถเลิกการให้สิทธิในการใช้ข้อมูลส่วนตัวของข้าพเจ้าได้ โดยต้องแจ้งให้ผู้วิจัยทราบ

ข้าพเจ้าได้ตระหนักว่าข้อมูลในการวิจัยรวมถึงข้อมูลทางการแพทย์ที่ไม่มีการเปิดเผยชื่อ จะผ่านกระบวนการต่าง ๆ เช่นการเก็บข้อมูล การบันทึกข้อมูลในคอมพิวเตอร์ การตรวจสอบ การวิเคราะห์ และการรายงานเพื่อวัตถุประสงค์ทางวิทยาศาสตร์รวมทั้งการใช้ข้อมูลทางการแพทย์ในอนาคตหรือการวิจัยทางด้านเภสัชภัณฑ์เท่านั้น

ข้าพเจ้ายินดีลงนามในเอกสารยินยอมนี้เพื่อร่วมการวิจัยด้วยความเต็มใจ

....ลงนามผู้ยินยอม

(...) ชื่อผู้ยินยอม ตัวบรรจง

วันที่................เดือน...พ.ศ....................

ข้าพเจ้าได้อธิบายถึงวัตถุประสงค์การวิจัย วิธีวิจัย อันตราย หรืออาการที่อาจเกิดจากการวิจัย รวมทั้งประโยชน์ที่จะเกิดขึ้นจากการวิจัยอย่างละเอียด ให้ผู้เข้าร่วมโครงการวิจัยตามนามข้างต้นได้ทราบและมีความเข้าใจดีแล้ว พร้อมลงนามในเอกสารแสดงความยินยอมด้วยความเต็มใจ

....ลงนามผู้ทำวิจัย

(..) ชื่อผู้วิจัย ตัวบรรจง

วันที่.................เดือน...พ.ศ....................

.... ลงนามพยาน

(..) ชื่อพยาน ตัวบรรจง

วันที่................เดือน..พ.ศ....................
