# Supplementary material for: Correction of vitamin D deficiency facilitated suppression of IP-10 and DPP IV levels in patients with chronic hepatitis C: A randomised double-blinded, placebo-control trial
Source: PLoS One. 2017 Apr 4;12(4):e0174608. doi: 10.1371/journal.pone.0174608 (PMC5380326; doi:10.1371/journal.pone.0174608)
Supplement: S3 File — (PDF) [file pone.0174608.s003.pdf]

Submission proposal for

INSTITUTIONAL REVIEW BOARD

FACULTY OF MEDICINE, CHULALONGKORN UNIVERSITY

BANGKOK, THAILAND

Table of contents Page

Front page	02
1. Background	03
2. Hypothesis and research questions	05
3. Research design and methodology	06
4. References	10
5. Appendix	12
5.1 Vitamin D dosage	12
5.2 Case record form	13
5.3 Patient Information sheet	17
5.3 Informed consent sheet	23

Research proposal submitted to

INSTITUTIONAL REVIEW BOARD

FACULTY OF MEDICINE, CHULALONGKORN UNIVERSITY

BANGKOK, THAILAND

Title: Effect of vitamin D replacement associated with adaptive immune responses in chronic hepatitis C patients with vitamin D deficiency

Principle investigators:

1. Dr. Kriangsak Charoensuk
2. Dr. Piyawat Komolmit

Affiliation: Division of Gastroenterology and Hepatology
Department of Medicine, Faculty of Medicine
Chulalongkorn University

Key word: vitamin D deficiency, vitamin D replacement, chronic hepatitis C, immune response, inducible protein-10 (IP-10)

1. Background

Vitamin D is important for several organ systems in the body. It is a fat soluble vitamin which usually acquired from foods and supplements for example fish oil, fatty foods, milk, liver, Tuna fish, egg York and mostly generating from UV-B (290-315 nm) from sunlight. Vitamin D is changed to active form, 25-hydroxy vitamin D and 1, 25-hydroxy vitamin D in liver and kidney, respectively. Parathyroid hormone also involves in vitamin D, calcium and phosphorus metabolism [1, 2].

Several studies suggested association of vitamin D with prevention and treatment of diseases related to bone and joint, heart disease, multiple sclerosis, diabetes type 1, immune related diseases and malignancies [3-5]. Vitamin D also involves in keeping balance of innate and adaptive immune responses [6, 7]. Vitamin D suppresses T helper 1 and activates T helper 2 cells activities demonstrated by reduction of IL-2, IL-12 and IFN gamma and increasing production of IL-4 and TGF beta [7-12].

Definition of vitamin D deficiency is controversial, however, mostly accepted the level of 30 ng/mL as a cut point. The level above 150 ng/mL can cause toxicity [2]. Vitamin D deficiency has been reported in high prevalence from several regions from 40 – 100% [5].

Definition of vitamin D deficiency:

25-hydroxy vitamin D levels	> 30	ng/mL	normal
	20-30	ng/mL	mild deficiency
	10-20	ng/mL	moderate deficiency
	< 10	ng/mL	severe deficiency

Liver is an import organ for vitamin D metabolism. Pre-vitamin D and vitamin D derived from food and skin is transported to liver and hydroxylated to 25-hydroxy vitamin D which subsequently transformed to active metabolite, 1, 25-hydroxy vitamin D in kidney (ref 2). Several studies reported vitamin D deficiency in chronic liver diseases both cholestatic and non-cholestatic liver diseases, eg. primary biliary cirrhosis and cirrhosis from other causes, which increased risks of osteoporosis/malacia and fractures [12-14].

Viral hepatitis C (HCV), an RNA virus in Flaviviridae family, is a common cause of chronic hepatitis, cirrhosis and hepatocellular carcinoma. Approximately 2.7 million people worldwide are infected by HCV. Both innate and adaptive immune responses involve in body defences to HCV infection. The innate immune responses actions via interferon alpha and beta from infected hepatocytes and plasmacytoid dendritic cells, which result in reduction of HCV viral load, increase expression of HLA class I on antigen presenting cells, activated NK cells and cytotoxic T cells [7, 8, 11].

HCV activates innate immune responses through activation of macrophage, natural killer cells and neutrophils, which secrete inflammatory cytokines. Dendritic cells subsequently activated adaptive immune responses from CD4+ and CD8+ T cells which help body to get rid of the virus [9, 15].

Recent studies suggested vitamin D deficiency associated with reduction of sustained virological responses (SVR) in HCV treatment with pegylated interferon/ribavirin regimen. In addition, supplement vitamin D to chronic hepatitis C patients help to improve SVR. The explanation of these results might be that vitamin D corrects or improves the imbalance of the immune responses in HCV infection [16, 17].

Inducible protein-10 (IP-10) or CXCL-10 is a CXC chemokine which involves in chemotaxis of several immune cells and it acts through the CXCR3 receptor on the cell surface [18, 19]. Increased expression of CXCR3 and IP-10 mRNA were demonstrated in CHC patients. IP-10 levels is a predictor for HCV treatment responses. High IP-10 levels associated with lower response to Pegylated interferon/ribavirin treatment in CHC patiens [20-22].

One important link between vitamin and IP-10 was demonstrated in invitro model of monocytes activated by vitamin D. The TNF alpha and IP-10 levels were increased and decreased, respectively upon activation by vitamin D [23].

All in all, vitamin D involves in various body functions and immune responses. We postulated that vitamin D supplement might associated with changing of adaptive immune response cytokines (TH1 or TH2 related cytokines) and IP-10 levels in CHC patients in the way that help to improve treatment responses.

2. Research questions and hypothesis

2.1 Research questions

2.1.1 Primary research question: Vitamin D supplements result in changes of serum immune cytokines' levels in CHC patients, both in TH1 and TH2 cytokines.

2.1.2 Secondary research question: Vitamin D supplements in CHC patients results in changes of IP-10 levels and other specific cytokines/enzymes involved in chronic HCV infection.

2.2 Objectives

2.2.1 To study the results of vitamin D supplement on immune responses in CHC patients with vitamin D deficiency

2.2.2 To study the association of vitamin D supplement on changes of serum IP-10 levels and other specific cytokines/enzymes related to CHC patients with vitamin D deficiency

2.3 Hypothesis

Vitamin D supplement in CHC patients with vitamin D deficiency changes the adaptive immune responses in favour of immune controlling of the HCV.

2.4 Key words

Vitamin D deficiency, Vitamin D replacement, Chronic hepatitis C, Immune response, Inducible protein-10 (IP-10)

2.5 Expected benefits and application

If the results suggested the benefits of vitamin D supplement in CHC patients with vitamin D deficiency, CHC patients should be screen for vitamin D deficiency. Further studies on vitamin D and specific treatment of HCV (PegIFN/RBV or other regimens) should be considered. In addition, the finding will connect the dots of scientific knowledge of the cytokines changes in CHC (for example IP1-0 and DPP-4 levels) and how vitamin D link to the improve treatment responses with vitamin D supplements.

3. Research design and methodology

3.1 study design:

Randomized double-blind placebo-controlled trial

3.2 Population

3.2.1 **Target population**: Patients with chronic hepatitis C who follow up and prepare for (naïve) or previous relapsers/non-responders to pegylated interferon/ribavirin treatment (at least 12 months) in the liver clinic, King Chulalongkorn Memorial Hospital, between January and December 2012.

3.2.2 **Inclusion criteria**

a) Thai patients age between 18 – 65 years old

b) Compensated liver diseases (albumin > 3.4, Total bilirubin < 1.5 mg/dL,

albumin > 3.4 mg/dL, INR < 1.2 and Platelet > 100,000)

3) No history of chronic renal diseases (creatinine < 1.5 mg/dL)

4) Agree with the protocol of the research and accepted for blood tests

3.2.3 Exclusion criteria: patients with

a) Decompensated cirrhosis

b) Co-infection with other viral hepatitis

c) Immune deficiency, AIDs

d) Autoimmune diseases

e) Active infections

f) Immunosuppressive drugs

g) Not agree for the research protocol

3.3 Sample size calculation

As there is no previous study on serum TH-1/TH-2 cytokines or IP-10 levels in this regard, the sample size was an estimation for general study to assess immunological parameters.

3.4 statistical analysis

Basic data were calculated and presented in mean±SD for quantitative and in percent for qualitative values. The number of cases in supplement and control groups were set at 40 cases per group.

The comparisons of TH-1 and TH-2 and IP-10 and other specific cytokines/enzymes between cases and controls at baseline and at the end of 6 weeks were calculated using paired-T-test.

The comparisons of TH-1 and TH-2 and IP-10 and other specific cytokines/enzymes between cases and controls at baseline and at the end of 6 weeks in between groups were calculated using analysis of co-variance (ANCOVA model).

3.5 Measurements

a) Vitamin D levels (25 Hydroxy vitamin D) were measured at baseline and at 6 weeks.

b) TH-1/TH-2, IP-10 and other specific cytokines/enzymes were measured using ELISA kits of BioRad (Bioplex Th1/Th2 panel)

c) IP-10 levels were measured using commercial set: Quantikine human CXCL10/IP10 immunoassay (R&D Systems) with 1:2 dilution for IP-10 levels between 8 – 500 pg/mL and 1:5 dilution for IP-10 levels above 1000 pg/Ml. (All experiments were performed in the immuno lab in Or-Por-Ror building, level 18.)

3.6 Intervention

a) Known CHC patients were interviewed for history and performed physical examination.

b) CHC patients' history and data were recorded in CRF (appendix)

c) All patients were screened for vitamin D deficiency. If a patient has vitamin D deficiency the detail protocol will be informed and requested for consent according to patient preference.

10 mL of blood were collected in clotted blood (5 mL) and EDTA tube (5 mL) and kept in -70C storage at baseline and at 6 weeks. All samples were evaluated simultaneously at the end of study.

d) Patients are stratified using block of four randomizations method (computer generated sequences). Each group contains 40 cases, vitamin D supplement cases and placebo controls. The patients in each group were prescribed vitamin D or placebo at dosage according protocol (appendix).

e) At 6 weeks, patients' blood will be collected for vitamin D and cytokines' levels measurements.

All patients who remain having vitamin D deficiency at the end of study, vitamin D tablet will be prescribed for supplement as replacement protocol (appendix).

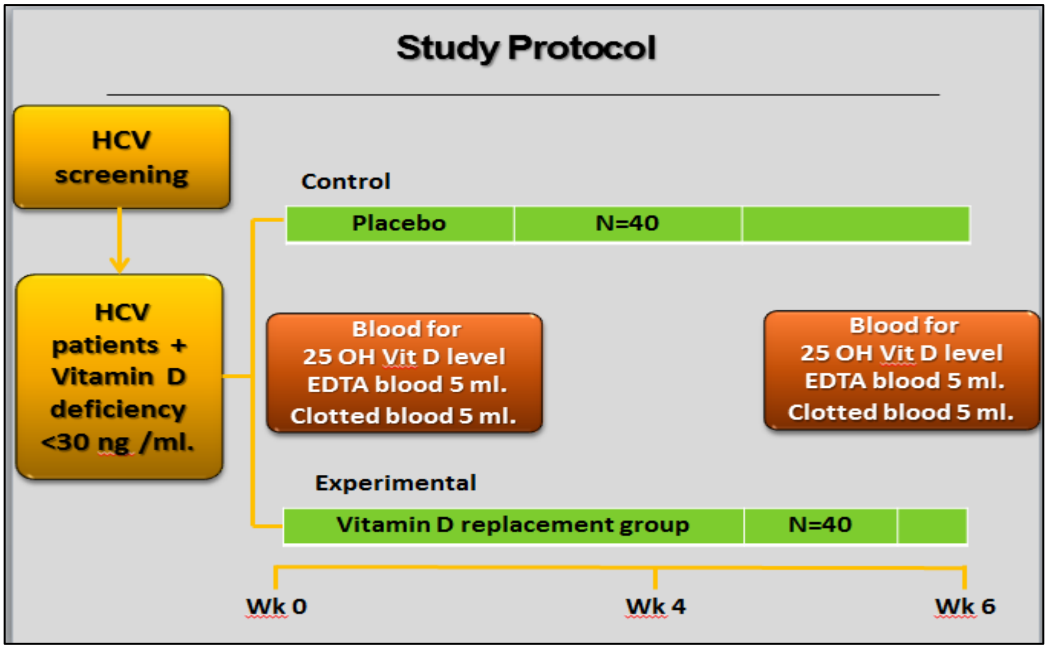

3.7 Administration and time schedule

Administration	2011		2012					2013					2014				
	8	12	2	4	6	8	12	2	4	6	8	12	2	4	6	8	12
1.Preparation	←	→															
2. Perform researches				←	↔	↔	↔	↔	→								
3. Initial analysis										←	↔	→					
4. Additional parameters Measurement (DPP-4, Th-17 and others)													←	↔	→		
5. Final analysis																←	→

3.8 Initial budgets

Items	Cost (Baht)
Measurement of vitamin D levels	288,000
Measurement of cytokines (Th1/Th2) 80 kits/set	150,000
Measurement of Inducible protein – 10 (IP-10) 80 kits/set	42,000
Cost of vitamin D tablets	3,000
Other laboratory consumes	5,000
Cost of placebo tablets	5,000
**รวมจำนวนเงินทั้งสิ้น**	500,000

4. References

1. Holick CN, Stanford JL, Kwon EM, Ostrander EA, Nejentsev S, Peters U. Comprehensive association analysis of the vitamin D pathway genes, VDR, CYP27B1, and CYP24A1, in prostate cancer. Cancer epidemiology, biomarkers & prevention : a publication of the American Association for Cancer Research, cosponsored by the American Society of Preventive Oncology. 2007;16(10):1990-9. Epub 2007/10/13. doi: 10.1158/1055-9965.epi-07-0487. PubMed PMID: 17932346.
2. Holick MF. Vitamin D deficiency. The New England journal of medicine. 2007;357(3):266-81. Epub 2007/07/20. doi: 10.1056/NEJMra070553. PubMed PMID: 17634462.
3. Ardizzone S, Cassinotti A, Bevilacqua M, Clerici M, Porro GB. Vitamin D and inflammatory bowel disease. Vitamins and hormones. 2011;86:367-77. Epub 2011/03/23. doi: 10.1016/b978-0-12-386960-9.00016-2. PubMed PMID: 21419280.
4. Deeb KK, Trump DL, Johnson CS. Vitamin D signalling pathways in cancer: potential for anticancer therapeutics. Nature reviews Cancer. 2007;7(9):684-700. Epub 2007/08/28. doi: 10.1038/nrc2196. PubMed PMID: 17721433.
5. Lange CM, Bojunga J, Ramos-Lopez E, von Wagner M, Hassler A, Vermehren J, et al. Vitamin D deficiency and a CYP27B1-1260 promoter polymorphism are associated with chronic hepatitis C and poor response to interferon-alfa based therapy. Journal of hepatology. 2011;54(5):887-93. Epub 2010/12/15. doi: 10.1016/j.jhep.2010.08.036. PubMed PMID: 21145801.
6. Baeke F, Takiishi T, Korf H, Gysemans C, Mathieu C. Vitamin D: modulator of the immune system. Current opinion in pharmacology. 2010;10(4):482-96. Epub 2010/04/30. doi: 10.1016/j.coph.2010.04.001. PubMed PMID: 20427238.
7. Bikle DD. Vitamin D and the immune system: role in protection against bacterial infection. Current opinion in nephrology and hypertension. 2008;17(4):348-52. Epub 2008/07/29. doi: 10.1097/MNH.0b013e3282ff64a3. PubMed PMID: 18660668.
8. Bikle DD. Vitamin D regulation of immune function. Vitamins and hormones. 2011;86:1-21. Epub 2011/03/23. doi: 10.1016/b978-0-12-386960-9.00001-0. PubMed PMID: 21419265.
9. Chambers ES, Hawrylowicz CM. The impact of vitamin D on regulatory T cells. Current allergy and asthma reports. 2011;11(1):29-36. Epub 2010/11/26. doi: 10.1007/s11882-010-0161-8. PubMed PMID: 21104171.
10. Hewison M. Vitamin D and the immune system: new perspectives on an old theme. Endocrinology and metabolism clinics of North America. 2010;39(2):365-79, table of contents. Epub 2010/06/01. doi: 10.1016/j.ecl.2010.02.010. PubMed PMID: 20511058; PubMed Central PMCID: PMCPmc2879394.
11. Lange NE, Litonjua A, Hawrylowicz CM, Weiss S. Vitamin D, the immune system and asthma. Expert review of clinical immunology. 2009;5(6):693-702. Epub 2010/02/18. doi: 10.1586/eci.09.53. PubMed PMID: 20161622; PubMed Central PMCID: PMCPMC2812815.
12. Maruotti N, Cantatore FP. Vitamin D and the immune system. The Journal of rheumatology. 2010;37(3):491-5. Epub 2010/01/19. doi: 10.3899/jrheum.090797. PubMed PMID: 20080911.
13. Chailurkit LO, Kruavit A, Rajatanavin R. Vitamin D status and bone health in healthy Thai elderly women. Nutrition (Burbank, Los Angeles County, Calif). 2011;27(2):160-4. Epub 2010/04/16. doi: 10.1016/j.nut.2009.12.001. PubMed PMID: 20392596.
14. Fisher L, Fisher A. Vitamin D and parathyroid hormone in outpatients with noncholestatic chronic liver disease. Clinical gastroenterology and hepatology : the official clinical practice journal of

the American Gastroenterological Association. 2007;5(4):513-20. Epub 2007/01/16. doi: 10.1016/j.cgh.2006.10.015. PubMed PMID: 17222588.
15. Bitetto D, Fattovich G, Fabris C, Ceriani E, Falleti E, Fornasiere E, et al. Complementary role of vitamin D deficiency and the interleukin-28B rs12979860 C/T polymorphism in predicting antiviral response in chronic hepatitis C. Hepatology (Baltimore, Md). 2011;53(4):1118-26. Epub 2011/04/12. doi: 10.1002/hep.24201. PubMed PMID: 21480318.
16. Kim TY. Role of vitamin D in chronic hepatitis C.
17. Lagging M, Askarieh G, Negro F, Bibert S, Soderholm J, Westin J, et al. Response prediction in chronic hepatitis C by assessment of IP-10 and IL28B-related single nucleotide polymorphisms. PloS one. 2011;6(2):e17232. Epub 2011/03/11. doi: 10.1371/journal.pone.0017232. PubMed PMID: 21390311; PubMed Central PMCID: PMCPMC3044738.
18. Luster AD. Chemokines--chemotactic cytokines that mediate inflammation. The New England journal of medicine. 1998;338(7):436-45. Epub 1998/02/12. doi: 10.1056/nejm199802123380706. PubMed PMID: 9459648.
19. Murdoch C, Finn A. Chemokine receptors and their role in inflammation and infectious diseases. Blood. 2000;95(10):3032-43. Epub 2000/05/16. PubMed PMID: 10807766.
20. Diago M, Castellano G, Garcia-Samaniego J, Perez C, Fernandez I, Romero M, et al. Association of pretreatment serum interferon gamma inducible protein 10 levels with sustained virological response to peginterferon plus ribavirin therapy in genotype 1 infected patients with chronic hepatitis C. Gut. 2006;55(3):374-9. Epub 2005/09/10. doi: 10.1136/gut.2005.074062. PubMed PMID: 16150856; PubMed Central PMCID: PMCPMC1856069.
21. Reiberger T, Aberle JH, Kundi M, Kohrgruber N, Rieger A, Gangl A, et al. IP-10 correlates with hepatitis C viral load, hepatic inflammation and fibrosis and predicts hepatitis C virus relapse or non-response in HIV-HCV coinfection. Antiviral therapy. 2008;13(8):969-76. Epub 2009/02/07. PubMed PMID: 19195322.
22. Zeremski M, Markatou M, Brown QB, Dorante G, Cunningham-Rundles S, Talal AH. Interferon gamma-inducible protein 10: a predictive marker of successful treatment response in hepatitis C virus/HIV-coinfected patients. Journal of acquired immune deficiency syndromes (1999). 2007;45(3):262-8. Epub 2007/04/07. doi: 10.1097/QAI.0b013e3180559219. PubMed PMID: 17414926.
23. Kuo YT, Kuo CH, Lam KP, Chu YT, Wang WL, Huang CH, et al. Effects of vitamin D3 on expression of tumor necrosis factor-alpha and chemokines by monocytes. Journal of food science. 2010;75(6):H200-4. Epub 2010/08/21. doi: 10.1111/j.1750-3841.2010.01704.x. PubMed PMID: 20722932.

5. Appendix

5.1 Vitamin dosage and replacement protocol

Diagnosis	Vitamin D level (ng /mL)	Replacement Total dose (IU) / week	Ergocalciferol (D2) 20,000 IU/ tab (Take with meal)	Treatment duration
Optimal	> 30	-	-	-
Mild deficiency (insufficiency)	20 – 30	60,000	2 tab Monday and 1 tab Friday	6 weeks
Moderate deficiency	10 – 20	80,000	2 tab Monday and 2 tab Friday	6 weeks
Severe deficiency	< 10	100,000	3 tab Monday and 2 tab Friday	6 weeks

5.2 Case Record Form

<table>
<tr><td colspan="3">Effect of vitamin D replacement on adaptive immune response in chronic hepatitis C patients with vitamin D deficiency

Investigators : Kriangsak Charoensuk , MD. , Piyawat Komolmit, MD,PhD.</td><td colspan="2">Case number

<table><tr><td>Year</td><td></td></tr></table></td></tr>
<tr><td colspan="2"></td><td>Age (years)</td><td colspan="2">Sex .... Male
.... Female</td></tr>
<tr><td rowspan="2">Baseline Characteristics

Weight .... kgs.

Height .... cms.

BMI .... kgs./m^2

Date..

Liver Biopsy

Metavir score ....

Ishak Score..</td><td colspan="2">Date of start treatment (DD/MM/YYYY)</td><td colspan="2"></td></tr>
<tr><td colspan="4">Baseline 25(OH)Vitamin D level (ng/dl.)

☐ < 10 ng/dl.
☐ 10 - 20 ng/dl.
☐ 20 – 30 ng/dl.
☐ > 30 ng/dl.

After treatment Vitamin D level ....ng/dl.</td></tr>
<tr><td rowspan="4">Underlying disease
☐ No ☐ Yes specify
____
____
____
Current medications
☐ No ☐ Yes specify
____
____
____
HCV diagnosis:
Duration of HCV diagnosis____________months
HCV risk factor(s)
• Heterosexual
• Homosexual
• IVDU
• Blood transfusion
• Tattoo
• Unknown
• Other please define_______________</td><td colspan="4">Baseline HCV genotype and VL prior treatment</td></tr>
<tr><td colspan="2">Date of exam

(DD/MM/YYYY)</td><td>HCV genotype</td><td>VL (copies/ml)</td></tr>
<tr><td colspan="2"></td><td></td><td></td></tr>
<tr><td colspan="2"></td><td></td><td></td></tr>
<tr><td colspan="5"></td></tr>
</table>

History of previous treatment				
Previous HCV treatment ☐ Yes ☐ No				
Regimen	Start date	Stop date	VL	Note

Treatment and Follow up Period

Regimens	Date of Follow up	HCV VL	Note	Important Lab

Laboratory Finding

Date ________________________

Complete Blood Count

Hct________Hb________ MCV ____________

Wbc __________________

%PMN _____________ ANC ______________

%Lymph ______________

%Mono_________ % Eosinophil _____________ Platelet Count ___________

PT _______________

PTT _______________

INR _______________

VitaminD 25(OH)D

Baseline _____________________ ng/dl.

After treatment _______________ ng/dl.

Inducible protein-10 (IP-10) level

____

Blood Chemistry

BUN_______Cr_______

Electrolyte

Na ____________ K ____________

Cl _____________ CO2 __________

Ca ____________ Phosphate ___________

Liver Function Test

TB ____________ DB ____________

Albumin _______ Globulin _______

SGOT _________ SGPT __________

ALP ____________

Immune function (Cytokine)

T Helper-1 ______________________________

T Helper-2 ______________________________

____

Serology profile

Anti HCV ______________ HBsAg ____________ Anti- HBsAg ____________

Anti HIV ______________

Lab Follow up

Date __________________

Hct ______Hb_____ MCV _____ Wbc ________%PMN _______%Lymph _______ ANC ________

Platelet Count _____________PT ______PTT _______INR _________

BUN_______Cr_______ TB_____ DB _____Albumin _______ Globulin _______

SGOT _________ SGPT _________ALP __________

HCV Viral loads ___________________

Date __________________

Hct ______Hb_____ MCV _____ Wbc ________%PMN _______%Lymph _______ ANC ________

Platelet Count _____________PT ______PTT _______INR _________

BUN_______Cr_______ TB_____ DB _____Albumin _______ Globulin _______

SGOT _________ SGPT _________ALP __________

HCV Viral loads __________________

Treatment

___OPD ___IPD ward___________

O Vitamin D2 dose ______________ IU/wk Duration_________weeks

O Vitamin D3 dose ______________ Duration_________weeks

O Pegylated interferon 2a dose________µg/kg/day Duration_________weeks

O Pegylated interferon 2b dose________µg/kg/day Duration_________weeks

O Ribavirin dose ________________ mgs. /day Duration_________weeks

O Adjusted dose drugs O No O Yes specify __________________________________

Date of dose adjustment ____________________ Reason _________________________

____

Record by _______________________

Date ____________________________

Lab Summary Record Form

Lab Summary Record form

Effect of vitamin D replacement on adaptive immune response in chronic hepatitis C patients with vitamin D deficiency

Investigators : Kriangsak Charoensuk , MD. , Piyawat Komolmit, MD, PhD.

Week 1: O EDTA blood ≥5 mls.
O Clotted bloods ≥ 5 mls.
O 25 (OH) vitamin D level

Week 4 O 25 (OH) vitaminD

Week 6 O EDTA blood ≥5 mls.
O Clotted bloods ≥ 5 mls.
O 25 (OH) vitamin D level

HN	Name	Vitamin D level Wks 1	Vitamin D Replacement Regimen	Vitamin D level Wks 4	Vitamin D level Wks 6	IP-10
		Date		Date	Date	
		Date		Date	Date	
		Date		Date	Date	
		Date		Date	Date	

5.3 Information for participant

Title of research: Effect of vitamin D replacement associated with adaptive immune responses in chronic hepatitis C patients with vitamin D deficiency

Principle investigators

Principle investigators:

1. Dr. Kriangsak Charoensuk
2. Dr. Piyawat Komolmit

Affiliation: Division of Gastroenterology and Hepatology

Department of Medicine, Faculty of Medicine

Chulalongkorn University

Tel. 02-2564000 ext. 4356 no. 2 or 081-8697003 (Dr. Kriangsak)

Tel. 02-2564000 ext. 4356 no. 2 (Dr. Piyawat)

To participants

You are invited to participate in this clinical trial as you have chronic hepatitis C and found to have vitamin D deficiency. Before your agreement to involve in this trial, please read this document thoroughly for your understanding of the reason of the research and the detail of the protocol. If you have any questions, please ask our investigator team or principle investigators.

You can ask for advice from your family, friend or your general practitioner. You have time for your decision freely. If you decide to participate in this trial, please sign the agreement at the end of this document.

Background

Vitamin D is important for several organ systems in the body. It is a fat-soluble vitamin which usually acquired from foods and supplements for example fish oil, fatty foods, milk, liver, Tuna fish, egg York and mostly generating from UV-B (290-315 nm) from sunlight. Vitamin D is changed to active form, 25-hydroxy vitamin D and 1, 25-hydroxy vitamin D in liver and kidney, respectively. Parathyroid hormone also involves in vitamin D, calcium and phosphorus metabolism

Several studies suggested association of vitamin D with prevention and treatment of diseases related to bone and joint, heart disease, multiple sclerosis, diabetes type 1, immune related diseases and malignancies. Vitamin D also involves in keeping balance of innate and adaptive immune responses. In view of scientific knowledge, vitamin D suppresses T helper 1 and activates T helper 2 cells activities demonstrated by reduction of IL-2, IL-12 and IFN gamma and increasing production of IL-4 and TGF beta.

For the last 3-5 years, several studies reported vitamin D deficiency in chronic liver diseases and cirrhosis from any causes, which increased risks of osteoporosis/malacia and fractures. Recent studies suggested vitamin D deficiency associated with reduction of sustained virological responses (SVR) in HCV treatment with pegylated interferon/ribavirin regimen. In addition, supplement vitamin D to chronic hepatitis C patients help to improve SVR. The explanation of these results might be that vitamin D corrects or improves the imbalance of the immune responses in HCV infection.

Inducible protein-10 (IP-10) is a substance in our body which involves in stimulation of several cells via a receptor on the cell surface. Increased expression of the receptor and IP-10 mRNA were demonstrated in CHC patients. Evidences suggested that IP-10 links to cellular mechanisms in chronic hepatitis C infection. Measurement of serum IP-10 levels helps to predict the chance of HCV treatment responses. High IP-10 levels associated with lower response to Pegylated interferon/ribavirin treatment in CHC patiens.

One important link between vitamin and IP-10 was demonstrated in invitro model of monocytes activated by vitamin D. The TNF alpha and IP-10 levels were increased and decreased, respectively upon activation by vitamin D.

All in all, vitamin D involves in various body functions and immune responses. We postulated that vitamin D supplement might associated with changing of T cells associated immune response cytokines, IP-10 levels or other specific cytokines/enzymes in CHC patients in the way that help to improve treatment responses.

Objective of the study

1. To study the effect of vitamin D supplement on the immune responses in chronic hepatitis C patients

2. To study the effect of vitamin D supplement on serum levels of IP-10 or other specific cytokines/enzymes.

Number of participants:

80 persons

Methods involve in this trial

After your agreement to participate in this trial, our investor team would like to check your blood (10 mL) for vitamin D and keep for measurement of various substances.

If you have vitamin D deficiency and have no conditions that should not be in the trial, we will arrange appointment to see the doctor for general physical examination and evaluation for the result of blood tests and receive the vitamin D or placebo tablets. The time period of the clinical trial is 6 weeks and you have to see the doctor twice (at baseline and at 6 weeks).

Responsibility of the participants

For success of this study, we would like to ask participants to have discipline to comply with the protocol. If you have any abnormal symptoms during this study, please contact the investigator team.

For your safety, you should not receive any vaccination or other medications by other doctors or from pharmacy. Please consult our investigator team if you need any question regarding other medications as they might have effect on vitamin D during the period of study.

Risk of the study

Any medications or event vitamins could have side effects of any severity. We would like to explain the risk and symptoms that might relate to the drugs involve in this study.

The chance of getting toxicity from vitamin is very low and the symptoms have been reported as tiredness, headache, anorexia, dry mouth, nausea and vomiting. The dosage of vitamin D in this trial is as of recommendation and the risk of adverse symptoms is low. However, if you experience any symptoms, please contact us for advice.

Risk from drawing blood sample

You may experience of pain at the puncture site, minor bleeding, ecchymosis, edema, syncope and local infection, which rarely happen.

Risk from other things

You might experience of some other symptoms not mention in this document as not seen before. For your safety, please report of any symptoms you may concern to the investigator team at any time.

IF there is any new reports of any safety concern regarding the medications used in this trial, we will inform all participants as soon as possible and you may decide to continue or pull out from the study.

How to see the doctor for your concern of any adverse events

You can contact the principle investigators or the team at any time in case you experience of some symptoms or concern of any adverse events. Immediate advice or treatment will be provided.

Benefit from the study

To participate in this trial, your health might be improved and reduced in severity. However, this will not be a guarantee.

Other methods or managements for the participants

You have no need to be in this clinical trial for expecting of the treatment. As there might be other ways of treatment for your disease. You may ask the doctor or your GP before making decision to participate in this clinical trial.

Practical points for participants during the trial

Please read carefully

- Please give your information regarding your health and history of diseases or treatment.
- Please inform our team if you experience any symptoms of concern
- Please abstain from other medications, herbs or un prescribed drugs from pharmacy
- Please inform the investigator team in cases you receive other new medications during the study period
- Please bring and return the tablets that have left after finishing the trial

Adverse events or complications happened during the trial and responsibility

If you have any complications during the trial, you will receive immediate treatment. Our investigator team will responsible for the cost of treatment and you signature at the end of the document does not mean that you disclaim from your regular health scheme.

If you experience any adverse events, you could contact the principle investigators by phone any time.

Your expenses for participation in this trial

You do not have to pay for vitamin D, doctor fee or cost of laboratory tests. The investigator will pay for your travel expense 250 Baht (7 US$) for each visit (2 visits required).

Participation or withdrawn from clinical trial

To participate in this clinical trial is you right to make decision and you could withdraw from the trial at any time. Your decision will not have any risk or consequence to your regular treatment of your diseases.

Our investigator team will withdraw you from the trial for your safety or for other following reasons:

- You could not comply with our protocol.
- You receive other medications preclude in this study.
- You have pregnancy during the trial.
- You experience some adverse events or abnormal laboratory results that may risk for your health.
- You have moderate or severe allergic reaction to the study drugs.
- You receive other medication that preclude for the study protocol.

Measurement for protection of participants' data

Your data and your name will be protected from any publicity. In case of publication, the name or address of the participant will be protected and the participant code number will be used instead.

After your agreement, the investigators will have the right to exam your data even after the trial finished. If you could withdraw that right at any time by contacting and inform in person or in writing and send it to the principle investigator (address shown).

If you withdraw from the trial, your add on personal data will not be done. However, some data will be used for evaluation. You could not return to the study protocol again after withdrawal.

After your agreement, the investigator could inform your GP regarding the agreement for participation in the trial.

Right of the participants

As you decide to be in this trial, you have the right as following

- You will receive information of the trial
- The investigators will inform regarding method of the study, drugs and other tests.
- You will receive information of risk or adverse event from the medications
- You will receive information of the benefit of the trial
- You will receive information regarding other alternative treatment that might benefit to your disease.
- You will receive information of management of adverse events or complications.
- You could ask for more information regarding process of the study.
- You will receive information regarding how and when to withdraw from the study which could be any time.
- You will receive the consent form with the signatures and date

- You have the right to make decision whether or not to participate the trial without any influence or pressure from anyone.

If you do receive any compensation for your adverse events related to trial medication or you do not receive proper management as the explanation in this document, you could contact the principle investigators directly or report to Institutional Review Board, Faculty of Medicine, Chulalongkorn University at the office on the 3rd floor Mahidol Building, King Chulalongkorn Memorial Hospital, Rama 4 Road, Pathumwan, Bangkok 10330, Tel. 02-256-4455, ext. 14 or 15 during office hour.

Thank you for your cooperation

5.4 Consent form for agreement to participate in the trial

Title of research: Effect of vitamin D replacement associated with adaptive immune responses in chronic hepatitis C patients with vitamin D deficiency.

Date of agreement: Date…………. Month………………Year……………..

I, Mr./Mrs./MS………………………………………………………………..……………….. Age…………….years

Current address

…….

…………………………………………………………………………………………Tel. …………………………………….

have read the information for the participant and agree to participate in this clinical trial.

I have received the copy of the consent form for participation in the trial and sign with the name and date include receiving the detail document for participant. I have received explanation regarding objective, period of study, methodology, risks that might happen and benefit of this trial. I have enough time to read and ask for any concern regarding the clinical trial and the investigators give all information without any hidden agenda.

I have the right to withdraw from the clinical trial at any time and without the need to explain the reasons. In addition, the withdrawal will not have any consequence to my disease management or my right to receive proper management.

The investigators confirm to protect the secrecy of my data and will reveal only on my permission.

Any investigation or examination of the data by other party including Institutional Review Board member have the right only to examine for the accuracy of the data. By this agreement, I accept for the examination of my previous health history.

The investigator agree to the participant that, in case of withdrawal, no more additional data will be kept and the data related to the participant will be abolished and could not be traced back to the participant.

I understand that I have a right to exam or correct my personal data and have a right of others to use my personal data by informing the investigators.

I understand that the research data including the health history will not be opened or report by participant name. And the data will be used to process by data correction, computer analysis of the data and then report only for scientific and clinical purpose.

I accept to sign this consent form for participation in the clinical trial with approval.

……………………………………………………………………………. Participant signature

……………………………………………………………………………. Block letters

Date……………..Month…………………year…………………

I have explained the information regarding this clinical trial including objective, methodology, risk or benefit of the trial and the participant as the above name has signed for the agreement to comply with the trial.

……………………………………………………………………………. Investigator signature

……………………………………………………………………………. Block letters

Date……………..Month…………………year…………………

……………………………………………………………………………. Witness signature

……………………………………………………………………………. Block letters

Date……………..Month…………………year…………………
