# Supplementary material for: Correction of vitamin D deficiency facilitated suppression of IP-10 and DPP IV levels in patients with chronic hepatitis C: A randomised double-blinded, placebo-control trial
Source: PLoS One. 2017 Apr 4;12(4):e0174608. doi: 10.1371/journal.pone.0174608 (PMC5380326; doi:10.1371/journal.pone.0174608)
Supplement: S4 File — (PDF) [file pone.0174608.s004.pdf]

Supplement statistics

	page
**1. SPSS log file (Syntax)**	1
**2. Log transformation of IP-10 and DPP-4 of table 2**	4
**3. Wilcoxon signed rank test for table 2**	4
**4. ANCOVA analysis**	5
**5. Cohen's d effect size calculation**	7

SPSS Log File

```
GET
  FILE='/Users/Kriangsak/Documents/Data_vitD.sav'.
DATASET NAME DataSet1 WINDOW=FRONT.
DESCRIPTIVES VARIABLES=randomization age sex weight height vitdpre vitdpost status SGOT SGPT Plt
FIB4 BMI Liverbiopsy FibrosisScore Metavir HAI Fattychange genotype VL VLlog IL2pre IL2post IL4pre
IL4post IL5pre IL5post IL10pre IL10post IL12pre IL12post IL13pre IL13post GMCSFpre GMCSFpost
Interferonpre Interferonpost TNFpre TNFpost IP10pre IP10post IL17PRE IL17POST filter_$ deltavitd
deltaip10 deltaIL2 deltaIL4 deltaIL5 deltaIL10 deltaIL12 deltaIL13 deltaIFN deltaTNF deltaGMCSF
deltaIL17 FIB4CAT LogIP10pre
LogIP10post delta_logIP10 DPP4pre DPP4post deltaDPP4 logDPP4pre logDPP4post deltaLogDpp4
  /STATISTICS=MEAN STDDEV MIN MAX.
```

Descriptives

```
EXAMINE VARIABLES=age sex weight height
  /ID=randomization
  /PLOT BOXPLOT STEMLEAF
  /COMPARE GROUPS
  /STATISTICS DESCRIPTIVES
  /CINTERVAL 95
  /MISSING LISTWISE
  /NOTOTAL.
USE ALL.
COMPUTE filter_$=(randomization = 1).
VARIABLE LABELS filter_$ 'randomization = 1 (FILTER)'.
VALUE LABELS filter_$ 0 'Not Selected' 1 'Selected'.
FORMATS filter_$ (f1.0).
FILTER BY filter_$.
EXECUTE.
```

T-TEST PAIRS=vitdpre IL2pre IL4pre IL5pre IL10pre IL12pre IL13pre GMCSFpre Interferonpre TNFpre IP10pre LogIP10pre DPP4pre logDPP4pre WITH vitdpost IL2post IL4post IL5post IL10post IL12post IL13post GMCSFpost Interferonpost TNFpost IP10post LogIP10post DPP4post logDPP4post (PAIRED)

/CRITERIA=CI(.9500)

/MISSING=ANALYSIS.

/WILCOXON=vitdpre IL2pre IL4pre IL5pre IL10pre IL12pre IL13pre GMCSFpre Interferonpre TNFpre IP10pre LogIP10pre DPP4pre logDPP4pre WITH vitdpost IL2post IL4post IL5post IL10post IL12post IL13post GMCSFpost Interferonpost TNFpost IP10post LogIP10post DPP4post logDPP4post (PAIRED)

/MISSING ANALYSIS.

Wilcoxon Signed Ranks Test

USE ALL.

COMPUTE filter_$=(randomization = 2).

VARIABLE LABELS filter_$ 'randomization = 2 (FILTER)'.

VALUE LABELS filter_$ 0 'Not Selected' 1 'Selected'.

FORMATS filter_$ (f1.0).

FILTER BY filter_$.

EXECUTE.

T-TEST PAIRS=vitdpre IL2pre IL4pre IL5pre IL10pre IL12pre IL13pre GMCSFpre Interferonpre TNFpre IP10pre LogIP10pre DPP4pre logDPP4pre WITH vitdpost IL2post IL4post IL5post IL10post IL12post IL13post GMCSFpost Interferonpost TNFpost IP10post LogIP10post DPP4post logDPP4post (PAIRED)

/CRITERIA=CI(.9500)

/MISSING=ANALYSIS.

NPAR TESTS

/WILCOXON=vitdpre IL2pre IL4pre IL5pre IL10pre IL12pre IL13pre GMCSFpre Interferonpre TNFpre IP10pre LogIP10pre DPP4pre logDPP4pre WITH vitdpost IL2post IL4post IL5post IL10post IL12post IL13post GMCSFpost Interferonpost TNFpost IP10post LogIP10post DPP4post logDPP4post (PAIRED)

/MISSING ANALYSIS.

Wilcoxon Signed Ranks Test

FILTER OFF.

USE ALL.

EXECUTE.

T-TEST GROUPS=randomization(1 2)

/MISSING=ANALYSIS

/VARIABLES=age vitdpre SGOT SGPT Plt FIB4 BMI FibrosisScore VL VLlog

/CRITERIA=CI(.95).

T-Test

[DataSet1] /Users/Kriangsak/Documents/Data_vitD.sav

T-TEST GROUPS=randomization(1 2)

/MISSING=ANALYSIS

/VARIABLES=vitdpre vitdpost IL2pre IL2post IL4pre IL4post IL5pre IL5post IL10pre IL10post IL12pre IL12post IL13pre IL13post GMCSFpre GMCSFpost Interferonpre Interferonpost TNFpre TNFpost IP10pre IP10post LogIP10pre LogIP10post DPP4pre DPP4post logDPP4pre logDPP4post

/CRITERIA=CI(.95).

T-Test

NPAR TESTS

/M-W= vitdpre vitdpost deltavitd deltaip10 deltaIL2 deltaIL4 deltaIL5 deltaIL10 deltaIL12 deltaIL13 deltaIFN deltaTNF deltaGMCSF delta_logIP10 deltaDPP4 deltaLogDpp4 BY randomization(1 2)

/MISSING ANALYSIS.

2. Log transformation of IP-10 and DPP IV in table 2

Table 2. Comparing mean serum levels of each parameter between pre- and post supplements in placebo and vitamin D groups. Data were transformed to log scale. The results did not change much interm of increase in IP-10 and DDP IV levels in placebo group without statistical significances, and decrease in IP-10 (however, become less significance) and DPP IV levels (interestingly, from trending turn to be significant).

Variables	Placebo			Vitamin D		
	Pre (±S.E.)	Post (±S.E.)	P-value	Pre (±S.E.)	Post (±S.E.)	P-value
Log IP-10	2.71±0.05	2.76±0.05	0.113	2.74±0.06	2.68±0.06	0.096
Log DPP IV	3.77±0.02	3.79±0.02	0.287	3.80±0.02	3.77±0.02	0.036

3. Wilcoxon signed rank test for table 2

Table 2. Comparing mean serum levels of each parameter between pre- and post-supplements in placebo and vitamin D groups. Non-parametric, Wilcoxon signed rank test result

**Variables**	**Placebo**	**Vitamin D**
	**P-value**	**P-value**
25(OH)D	0.120	<0.001
IP-10	0.101	0.08
DPP IV	0.767	0.025
IL-2	0.549	0.203
IL-4	0.830	0.533
IL-5	0.925	0.548
IL-10	0.939	0.799
IL-12	0.726	0.430
IL-13	0.638	0.728
IFN-γ	0.379	0.588
TNF-α	0.883	0.935
GM-CSF	0.737	0.673

4 ANCOVA analysis of table 2 (Log File) using SAS

ANCOVA analysis:
Copy from Log-window :

```
2429  %let path=e:\projects\us-science\Komolmit_feb2017;


NOTE: There were 80 observations read from the data set KOM.KOLMDATA1.
NOTE: The data set WORK.D0 has 80 observations and 67 variables.
NOTE: DATA statement used (Total process time):
      real time           0.30 seconds
      cpu time            0.03 seconds

2436  %macro ancv(var,varx,fn);
2437  proc glm data=d0;
2438  class randomization;
2439  model &var= &varx randomization/clm ;
2440  run;
2441  %mend;
2442
2443  %ancv(vitdpost, vitdpre,1);

2444  %ancv(IP10post,IP10pre,2);

NOTE: PROCEDURE GLM used (Total process time):
      real time           0.48 seconds
      cpu time            0.20 seconds

2445  %ancv(DPP4post,DPP4pre,3);

NOTE: PROCEDURE GLM used (Total process time):
      real time           0.40 seconds
      cpu time            0.21 seconds


2446  %ancv(il2post, il2pre,4);

NOTE: PROCEDURE GLM used (Total process time):
      real time           0.38 seconds
      cpu time            0.17 seconds



2447  %ancv(il4post, il4pre,5);

NOTE: PROCEDURE GLM used (Total process time):
      real time           0.38 seconds
      cpu time            0.20 seconds



2448  %ancv(il5post, il5pre,6);

NOTE: PROCEDURE GLM used (Total process time):
      real time           0.35 seconds
      cpu time            0.17 seconds


2449  %ancv(il10post, il10pre,7);

NOTE: PROCEDURE GLM used (Total process time):
      real time           0.35 seconds
      cpu time            0.21 seconds
```

```
2450  %ancv(il12post, il12pre,8);

NOTE: PROCEDURE GLM used (Total process time):
      real time           0.36 seconds
      cpu time            0.15 seconds



2451  %ancv(il13post, il13pre,9);

NOTE: PROCEDURE GLM used (Total process time):
      real time           0.43 seconds
      cpu time            0.12 seconds



2452  %ancv(Interferonpost, Interferonpre,10);

NOTE: PROCEDURE GLM used (Total process time):
      real time           0.42 seconds
      cpu time            0.21 seconds



2453  %ancv(TNFpost, TNFpre,11);

NOTE: PROCEDURE GLM used (Total process time):
      real time           0.35 seconds
      cpu time            0.23 seconds



2454  %ancv(GMCSFpost,GMCSFpre,12);

NOTE: PROCEDURE GLM used (Total process time):
      real time           0.41 seconds
      cpu time            0.20 seconds
```

5. Cohen 's d effect size analysis

Table 3. Comparing changes in serum levels (or delta) of each parameter in placebo and vitamin D groups during 6-week period of supplement.

Note: mean and SD for calculation of Cohen's D effect size

Changes of serum or delta (Δ) levels*	Placebo group Mean ± SD	Vitamin D group Mean ± SD	*P* values	Effect size
25(OH)D**	0.560±2.90	25.050±13.73	< 0.001	2.47
IP-10	83.270±340.76	-133.800±389.22	0.010	0.59
Log IP-10	0.05 log±0.19	-0.06 log±0.23	0.021	0.55
DPP IV**	255.46±1368.46	-521.79±1442.59	0.016	0.35
Log DPP IV**	0.016 log±0.093	-0.033 log±0.095	0.024	0.08
IL-2	5.630±35.21	-3.710±14.70	0.127	0.18
IL-4	-0.44±2.51	-0.20±3.13	0.755	0.22
IL-5	-0.45±3.35	0.45±6.11	0.425	0.24
IL-10	-0.61±6.73	2.69±19.80	0.328	0.00
IL-12	-3.06±36.41	8.67±58.00	0.292	0.05
IL-13	0.85±9.03	0.80±16.68	0.987	0.08
IFN-γ	-14.32±736.22	36.58±1120.85	0.815	0.02
TNF-α	-15.37±170.73	-29.01±189.84	0.742	2.47
GM-CSF	-0.18±27.42	-0.89±34.77	0.819	0.59

Cohen's $d = (M_2 - M_1) / SD_{\text{pooled}}$

where: $SD_{\text{pooled}} = \sqrt{((SD_1^2 + SD_2^2) / 2}$
