# Supplementary material for: Correction of vitamin D deficiency facilitated suppression of IP-10 and DPP IV levels in patients with chronic hepatitis C: A randomised double-blinded, placebo-control trial
Source: PLoS One. 2017 Apr 4;12(4):e0174608. doi: 10.1371/journal.pone.0174608 (PMC5380326; doi:10.1371/journal.pone.0174608)
Supplement: S5 File — (PDF) [file pone.0174608.s005.pdf]

id	randomiza	age	sex	weight	height	vitdpre	vitdpost	status	SGOT
1	1	50	0	78	170	29.30	60.30	0	90
2	2	35	1	65	160	17.40	19.10	0	60
3	1	35	1	56	168	26.60	58.40	0	30
4	1	50	0	55	155	19.10	60.40	0	28
5	1	61	1	50	152	23.40	41.40	1	48
6	2	64	1	70	165	24.20	24.70	1	102
7	1	50	1	49	152	20.50	62.70	0	91
8	2	45	0	63	173	17.90	20.20	0	118
9	2	44	0	61	160	16.60	19.90	0	43
10	2	48	0	65	172	11.50	15.50	1	40
11	1	69	0	73	156	17.90	58.90	1	47
12	2	58	1	85	175	24.90	25.50	0	68
13	1	52	0	65	168	22.90	46.40	1	26
14	2	49	1	57	158	17.50	14.40	1	194
15	1	50	1	56	170	24.60	36.10	1	33
16	1	57	1	88	176	27.50	57.60	0	55
17	1	53	1	55	155	19.60	57.30	1	72
18	2	55	0	68	158	22.50	23.60	1	55
19	2	67	1	65	165	24.10	21.10	1	29
20	1	66	0	62	152	15.30	33.70	1	33
21	2	52	1	64	154	20.10	25.50	0	267
22	2	66	0	60	148	22.40	22.70	1	19
23	1	56	0	75	165	13.40	29.50	1	80
24	1	52	0	66	167	20.30	58.50	1	21
25	1	50	0	68	168	28.20	38.90	1	32
26	2	54	1	50	150	23.80	21.50	1	68
27	1	55	0	92	165	28.10	50.50	1	66
28	1	50	0	64	158	27.20	76.70	0	69
29	2	48	1	56	160	20.50	21.50	0	47
30	2	55	1	82	155	22.50	25.60	1	69
31	1	58	1	65	155	15.60	44.00	1	44
32	1	52	1	68	160	12.10	56.30	0	89
33	2	36	0	60	169	29.70	24.90	0	25
34	2	32	1	72	165	16.00	12.10	1	101
35	2	52	0	71	170	25.00	28.70	0	58
36	2	60	1	58	155	20.90	21.50	0	80
37	1	52	0	65	168	22.90	46.40	0	47
38	2	41	0	71	175	20.20	20.90	0	34
39	2	59	1	85	154	20.30	19.70	0	54
40	1	46	0	55	160	21.40	56.30	0	47
41	1	47	0	71	170	19.30	32.00	1	88
42	1	41	0	61	161	24.70	52.40	0	57
43	1	35	1	48	158	28.90	58.70	0	37
44	2	47	0	70	167	18.60	20.10	0	94
45	2	62	1	65	153	14.70	16.80	0	38
46	1	67	1	58	153	25.60	41.90	0	181

47	2	58	1	43	154	10.40	15.00	1	35
48	1	52	0	74	173	16.90	34.30	0	105
49	2	65	1	55	156	23.40	23.80	0	141
50	2	47	0	69	175	23.00	24.50	0	63
51	2	30	1	52	155	17.50	20.00	0	16
52	1	49	0	61	163	17.70	34.90	0	59
53	2	36	0	65	172	22.80	24.60	1	29
54	1	60	1	61	159	23.50	30.50	1	99
55	2	52	0	74	169	27.00	32.20	0	32
56	1	60	1	76	170	13.10	27.30	1	108
57	2	60	0	66	159	22.30	25.10	0	140
58	2	40	0	75	173	26.20	31.00	0	35
59	1	51	0	71	170	21.80	64.40	0	40
60	1	46	0	51	150	25.90	81.10	1	36
61	1	59	1	52	165	16.90	60.70	1	74
62	1	60	1	77	162	13.70	34.60	1	34
63	2	65	0	102	173	23.00	18.90	1	48
64	2	70	0	84	170	14.40	13.40	1	19
65	1	65	1	56	158	13.00	45.00	1	37
66	2	53	0	51	165	16.80	10.40	1	41
67	1	33	0	67	170	26.20	43.50	0	50
68	2	66	1	55	151	17.40	16.40	1	42
69	2	65	0	64	160	24.20	24.00	1	38
70	1	57	1	63	160	18.20	17.00	0	54
71	2	69	1	52	149	17.00	18.20	1	168
72	1	56	0	62	171	17.80	38.90	0	36
73	2	49	1	46	150	10.00	11.50	0	128
74	1	37	0	66	162	15.10	23.60	0	50
75	2	55	1	44	156	11.70	11.00	1	47
76	1	55	1	92	168	21.10	24.30	0	50
77	2	35	0	66	159	28.00	28.20	0	323
78	1	52	0	80	170	29.30	47.20	1	28
79	1	59	0	60	168	10.50	14.50	0	323
80	2	42	0	56	169	24.30	19.40	1	28

SGPT	Plt	FIB4	BMI	Liverbiopsy	FibrosisScore	Metavir	HAI	Fattychang
102	143	3.21	26.99	0	#NULL!	#NULL!	#NULL!	#NULL!
56	132	2.13	25.39	0	#NULL!	#NULL!	#NULL!	#NULL!
28	162	1.22	19.84	0	#NULL!	#NULL!	#NULL!	#NULL!
11	105	4.02	22.89	0	#NULL!	#NULL!	#NULL!	#NULL!
40	136	2.79	21.64	0	#NULL!	#NULL!	#NULL!	#NULL!
112	142	4.34	25.64	1	2	1	6	35
123	179	2.25	21.21	0	#NULL!	#NULL!	#NULL!	#NULL!
122	226	2.13	21.05	1	2	2	10	5
101	56	3.36	23.83	1	2	1	6	80
33	203	1.65	21.97	0	#NULL!	#NULL!	#NULL!	#NULL!
71	186	2.07	30.00	0	#NULL!	#NULL!	#NULL!	#NULL!
83	56	7.73	27.76	1	4	2	11	5
31	135	1.80	23.03	0	#NULL!	#NULL!	#NULL!	#NULL!
164	166	2.26	22.67	0	#NULL!	#NULL!	#NULL!	#NULL!
42	220	1.16	19.38	0	#NULL!	#NULL!	#NULL!	#NULL!
62	162	4.47	28.41	0	#NULL!	#NULL!	#NULL!	#NULL!
89	256	1.58	22.89	1	3	1	9	5
68	219	1.68	27.24	1	2	2	10	10
28	129	2.85	23.88	1	2	1	9	9999
38	89	3.97	26.84	0	2	1	4	5
217	212	4.45	26.99	0	#NULL!	#NULL!	#NULL!	#NULL!
16	54	5.81	27.39	1	2	1	6	5
103	176	2.51	27.55	1	4	1	10	#NULL!
22	114	2.04	23.67	0	#NULL!	#NULL!	#NULL!	#NULL!
27	36	8.55	24.09	1	3	2	10	1
63	162	2.86	22.22	0	#NULL!	#NULL!	#NULL!	#NULL!
34	66	3.77	33.79	0	#NULL!	#NULL!	#NULL!	#NULL!
121	322	0.97	25.56	0	#NULL!	#NULL!	#NULL!	#NULL!
59	173	1.70	21.88	0	#NULL!	#NULL!	#NULL!	#NULL!
57	205	2.45	34.13	0	#NULL!	#NULL!	#NULL!	#NULL!
45	238	1.60	27.06	0	#NULL!	#NULL!	#NULL!	#NULL!
88	173	5.77	26.56	0	#NULL!	#NULL!	#NULL!	#NULL!
37	199	0.74	21.01	0	#NULL!	#NULL!	#NULL!	#NULL!
131	211	1.34	26.26	0	#NULL!	#NULL!	#NULL!	#NULL!
84	186	1.77	24.57	0	#NULL!	#NULL!	#NULL!	#NULL!
80	93	2.85	24.14	0	#NULL!	#NULL!	#NULL!	#NULL!
71	135	2.15	23.03	0	#NULL!	#NULL!	#NULL!	#NULL!
44	283	0.74	23.18	1	1	1	7	2
68	119	3.25	35.84	0	#NULL!	#NULL!	#NULL!	#NULL!
95	232	0.96	21.56	0	#NULL!	#NULL!	#NULL!	#NULL!
114	56	6.92	24.57	0	#NULL!	#NULL!	#NULL!	#NULL!
133	111	1.83	23.53	0	#NULL!	#NULL!	#NULL!	#NULL!
34	114	3.80	19.23	1	1	2	7	9999
104	144	1.95	25.10	1	3	1	9	30
28	208	2.14	27.77	0	#NULL!	#NULL!	#NULL!	#NULL!
161	308	3.10	24.78	0	#NULL!	#NULL!	#NULL!	#NULL!

28	86	4.46	18.13	0	#NULL!	#NULL!	#NULL!	#NULL!
90	321	1.79	24.73	0	#NULL!	#NULL!	#NULL!	#NULL!
144	102	7.49	22.60	0	#NULL!	#NULL!	#NULL!	#NULL!
180	308	0.72	22.53	0	#NULL!	#NULL!	#NULL!	#NULL!
15	111	1.12	21.64	0	#NULL!	#NULL!	#NULL!	#NULL!
107	139	2.01	22.96	0	#NULL!	#NULL!	#NULL!	#NULL!
39	252	0.66	21.97	1	1	1	3	9999
134	155	3.31	24.13	0	#NULL!	#NULL!	#NULL!	#NULL!
63	286	0.73	25.91	0	#NULL!	#NULL!	#NULL!	#NULL!
77	284	4.89	26.30	0	#NULL!	#NULL!	#NULL!	#NULL!
192	124	2.60	26.11	0	#NULL!	#NULL!	#NULL!	#NULL!
59	163	1.12	25.06	0	#NULL!	#NULL!	#NULL!	#NULL!
35	222	1.55	24.57	0	#NULL!	#NULL!	#NULL!	#NULL!
39	126	2.10	22.44	1	2	1	3	9999
114	154	2.66	19.10	0	#NULL!	#NULL!	#NULL!	#NULL!
61	144	1.81	29.15	0	#NULL!	#NULL!	#NULL!	#NULL!
77	186	1.91	34.08	0	#NULL!	#NULL!	#NULL!	#NULL!
22	119	2.38	29.07	0	#NULL!	#NULL!	#NULL!	#NULL!
28	236	1.93	22.43	1	1	1	6	9999
16	56	9.70	18.73	0	#NULL!	#NULL!	#NULL!	#NULL!
49	174	1.35	23.01	0	#NULL!	#NULL!	#NULL!	#NULL!
43	186	2.27	24.12	0	#NULL!	#NULL!	#NULL!	#NULL!
45	105	3.51	24.80	0	#NULL!	#NULL!	#NULL!	#NULL!
70	206	1.79	24.61	1	1	1	6	9999
94	266	4.49	23.42	0	#NULL!	#NULL!	#NULL!	#NULL!
34	222	1.56	21.20	0	#NULL!	#NULL!	#NULL!	#NULL!
85	155	4.39	20.44	1	3	2	10	9999
65	222	1.03	25.15	0	#NULL!	#NULL!	#NULL!	#NULL!
49	210	1.76	18.08	0	#NULL!	#NULL!	#NULL!	#NULL!
69	174	1.90	32.60	1	3	3	11	9999
183	186	4.49	26.11	0	#NULL!	#NULL!	#NULL!	#NULL!
28	105	2.62	27.68	0	#NULL!	#NULL!	#NULL!	#NULL!
183	206	6.84	21.26	1	2	1	8	9999
28	337	0.66	19.61	0	#NULL!	#NULL!	#NULL!	#NULL!

genotype	VL	VLlog	IL2pre	IL2post	IL4pre	IL4post	IL5pre	IL5post	IL10pre
1	0.00	0.00	0.86	0.86	19.89	28.64	18.42	21.40	19.44
1	0.00	0.00	0.86	0.86	13.35	25.70	12.50	21.82	14.75
3	########	5.47	0.00	0.86	10.03	8.55	9.98	6.00	3.15
3	74650.00	4.87	0.86	0.86	9.05	16.33	9.35	12.50	12.45
1	########	5.81	0.86	0.86	0.83	0.66	2.74	2.74	0.71
3	########	5.84	0.86	0.86	0.66	0.66	2.74	2.74	0.14
1	########	6.23	0.86	0.86	1.15	0.99	2.74	2.74	1.44
0	0.00	0.00	3.86	19.85	15.40	10.02	15.95	11.08	85.58
3	########	6.40	177.57	100.88	0.66	0.48	2.74	2.74	1.44
3	########	5.38	0.86	0.86	0.48	0.83	2.74	2.74	1.44
1	########	6.54	0.86	0.86	1.30	1.45	2.74	2.74	1.44
1	########	5.83	3.01	1.69	1.30	0.99	2.74	2.74	1.44
0	12474.00	4.10	0.86	0.86	0.34	0.34	2.74	2.74	1.44
1	########	5.24	0.86	0.86	2.09	2.02	6.67	4.69	14.87
1	########	5.93	124.92	74.49	0.83	0.29	2.74	2.74	1.44
1	########	6.80	0.86	2.17	1.81	1.30	1.60	1.60	1.85
0	0.00	0.00	30.91	27.62	0.99	0.99	2.74	2.74	1.44
1	########	5.47	0.86	0.86	1.30	1.30	2.74	2.74	0.71
3	########	5.84	0.86	0.86	1.15	1.45	2.74	2.74	1.44
1	########	5.47	0.86	0.86	5.53	5.41	14.81	21.17	128.56
0	3577.00	5.84	358.69	481.17	0.34	0.34	2.74	2.74	1.44
3	########	5.84	0.86	0.86	0.48	0.29	2.74	2.74	1.44
0	########	6.81	0.86	0.86	0.29	0.48	2.74	2.74	1.44
3	########	5.84	0.86	0.86	0.66	0.99	2.74	2.74	1.44
0	########	5.84	0.86	0.86	0.99	0.48	2.74	2.74	1.44
3	########	5.93	4.28	0.86	0.83	0.99	2.74	2.74	1.44
3	########	6.08	0.86	0.86	5.06	0.29	22.94	2.74	27.24
3	########	5.84	78.35	0.86	0.99	0.66	2.74	2.74	1.44
3	3600.00	6.12	0.86	0.77	0.48	3.54	20.33	2.74	1.44
3	########	3.55	0.86	67.29	0.74	0.29	2.74	2.74	1.44
1	21800.00	5.84	54.76	36.71	1.15	0.83	2.74	2.74	1.44
3	########	4.34	0.86	0.86	1.45	0.66	1.82	2.74	1.44
1	6356.00	6.13	5.52	4.70	1.30	1.30	2.74	2.74	3.39
1	########	3.80	0.86	0.86	0.99	0.83	2.74	2.74	1.44
3	########	5.33	0.86	0.86	2.16	1.88	2.74	2.74	2.48
1	########	6.65	0.86	0.86	1.45	0.66	2.74	2.74	1.44
1	########	5.84	0.86	5.73	1.15	0.83	2.74	2.74	1.44
3	########	6.54	0.86	0.86	0.66	0.66	2.74	2.74	1.44
0	0.00	0.00	19.66	0.77	0.07	0.29	2.74	2.74	4.36
1	########	5.93	0.86	0.86	1.30	1.22	2.74	0.89	1.44
1	########	6.20	0.86	0.86	0.48	0.66	2.74	2.74	1.44
3	########	6.94	0.86	0.86	0.48	0.83	2.74	2.74	1.44
3	########	7.04	3.86	6.34	11.57	15.74	72.72	99.19	145.87
1	########	5.84	296.37	447.39	0.91	0.99	2.74	2.74	1.44
0	########	6.09	0.86	0.86	1.07	1.15	2.74	2.74	1.44
0	0.00	0.00	0.86	0.86	4.95	5.03	2.29	6.40	3.57

1	########	5.84	0.86	0.86	3.96	6.68	1.81	1.47	2.18
1	3362.00	3.53	0.86	0.86	3.45	2.75	1.81	1.81	3.45
3	########	6.57	0.86	0.86	6.83	4.62	4.65	2.29	4.60
0	0.00	0.00	0.86	0.86	2.38	2.20	2.29	2.29	1.57
1	########	5.98	0.86	0.86	2.93	1.62	2.29	2.29	1.57
1	26356.00	4.42	0.86	0.86	4.21	0.66	1.81	2.29	0.96
1	########	5.83	0.86	0.86	3.01	1.00	2.29	2.29	1.57
0	########	6.43	0.86	0.86	5.90	3.10	5.56	2.29	2.96
1	########	5.90	0.86	0.86	2.01	2.93	2.29	2.29	5.89
1	########	5.08	0.86	0.86	6.98	3.62	8.78	2.29	220.09
3	600.00	2.78	0.86	0.86	5.11	4.62	2.29	2.29	4.60
1	########	5.90	0.86	0.86	2.93	2.20	2.29	2.29	1.13
0	0.00	0.00	0.86	0.86	3.28	5.90	2.29	4.65	6.10
1	########	5.84	0.86	0.86	7.43	7.51	7.03	9.06	4.15
1	########	6.75	1.13	0.86	8.40	6.52	8.51	5.20	4.15
1	########	5.40	0.86	0.86	4.62	6.14	3.40	5.38	0.55
3	########	5.65	0.86	0.86	2.38	1.00	2.29	2.29	1.57
1	########	6.49	0.86	0.86	4.79	2.75	3.84	2.29	2.45
0	########	5.43	9.06	1.43	4.30	3.96	2.29	1.07	1.57
1	########	5.84	0.86	0.86	0.66	3.45	2.29	2.29	1.57
3	########	5.44	0.86	0.86	5.75	5.03	6.07	2.93	1.30
1	########	6.39	0.86	0.86	5.35	4.79	1.81	2.93	4.82
1	########	5.54	0.86	0.86	4.13	5.27	0.54	4.25	0.55
3	44759.00	4.65	0.86	0.86	4.79	6.21	2.93	5.73	5.47
3	########	5.09	0.86	0.86	4.62	5.59	2.29	3.17	3.21
3	########	6.29	0.86	0.86	4.95	3.96	1.81	2.29	3.21
3	########	6.10	29.66	0.00	10.99	0.00	6.63	13.35	89.31
3	########	5.15	0.00	0.00	11.47	12.41	12.50	12.92	12.45
0	########	5.91	0.00	0.00	20.11	24.86	15.88	18.42	10.75
1	########	6.62	0.00	0.00	18.79	24.00	12.29	16.31	25.43
3	62319.00	4.79	0.00	0.00	9.05	10.51	9.35	9.98	3.15
1	########	6.38	0.00	0.00	29.98	18.34	21.82	18.00	19.44
3	########	6.05	0.00	0.00	9.05	19.01	9.98	17.36	7.96
1	########	7.59	0.00	0.00	17.90	12.88	17.58	12.92	15.33

IL10post	IL12pre	IL12post	IL13pre	IL13post	GMCSFpre	GMCSFpos	Interferon	Interferon	TNFpre
38.05	38.26	91.36	22.15	7.44	60.77	151.81	1307.92	1964.89	130.36
18.26	2.41	23.80	11.50	10.15	89.12	144.90	849.03	1551.00	93.76
3.41	2.10	2.41	6.07	1.88	5.68	4.76	631.96	534.19	41.70
10.75	13.99	19.56	10.15	20.17	35.55	42.81	757.62	923.65	93.76
0.14	3.69	3.69	0.99	2.32	0.70	0.70	4.39	8.12	1.91
1.44	3.69	3.36	0.33	2.32	0.70	0.70	8.12	8.12	12.26
1.44	5.72	7.81	2.32	2.32	0.70	0.70	27.02	100.60	1.91
47.06	423.77	221.33	50.57	33.53	339.33	227.60	7700.82	4415.25	812.54
1.44	7.11	5.72	2.73	2.04	0.70	0.70	8.12	8.12	1.91
1.44	4.36	4.02	2.32	2.32	0.70	0.70	8.12	4.39	0.22
0.14	5.04	5.72	1.53	1.53	0.70	1.11	27.02	48.56	0.22
0.14	5.72	4.36	2.32	2.32	0.70	0.70	37.87	8.12	9.40
1.44	2.58	2.58	2.32	2.32	0.70	0.70	8.12	8.12	6.67
11.32	26.33	19.49	3.81	1.27	0.70	0.70	380.63	269.67	7.23
1.44	4.36	4.36	0.99	0.33	0.70	0.70	15.93	4.39	0.22
0.95	7.81	7.11	5.77	3.39	15.94	2.47	1414.33	751.41	90.14
1.44	5.72	4.36	1.53	0.99	14.77	17.69	15.93	4.39	5.00
1.44	5.04	5.04	0.99	2.32	0.70	0.70	15.93	27.02	0.22
1.44	4.36	5.04	2.32	0.33	0.70	0.70	4.39	15.93	6.67
164.18	359.03	614.91	60.82	106.24	39.65	46.22	4878.68	7745.98	862.93
1.44	2.58	2.58	2.32	2.32	0.70	0.70	2637.39	5233.63	6.67
1.44	3.69	3.69	2.32	2.32	0.70	0.70	10.25	4.39	6.67
1.44	3.03	3.69	2.32	2.32	5.06	2.47	8.12	8.12	6.67
1.44	3.69	4.36	2.32	2.04	0.70	0.38	8.12	8.12	6.67
1.44	6.07	5.72	2.32	0.99	0.70	0.70	59.13	37.87	1.91
1.44	5.72	6.41	3.17	3.81	0.70	0.70	1430.54	1551.00	0.22
1.44	51.28	3.69	68.53	2.32	121.07	0.70	3467.10	517.54	416.68
1.44	6.41	5.04	3.39	0.33	0.70	0.70	997.02	938.42	1.91
10.18	4.36	39.63	2.32	54.05	0.70	83.48	3229.87	3567.73	15.06
1.44	5.72	4.36	2.32	2.32	0.70	0.70	131.10	64.38	9.40
1.44	5.72	4.36	2.32	2.32	0.70	0.70	788.33	500.78	6.67
1.44	7.11	5.72	2.73	2.27	0.70	0.70	181.18	131.10	16.45
5.58	5.72	5.04	3.39	4.61	11.21	14.77	757.62	757.62	3.48
1.44	5.04	4.36	2.32	2.32	0.70	0.70	1097.90	1140.54	5.00
1.44	5.72	7.11	3.39	5.01	0.70	0.70	90.33	80.00	3.48
1.44	7.11	4.36	3.81	2.32	0.70	0.70	27.02	21.51	5.75
1.44	4.36	4.36	2.32	2.32	11.21	11.21	27.02	6.67	6.67
1.44	11.37	9.94	2.04	0.99	0.70	0.70	48.56	27.02	5.00
4.83	3.03	2.38	2.32	2.32	0.70	0.70	8.12	8.12	6.67
1.44	6.41	4.36	2.32	2.32	1.80	0.70	1112.15	1695.62	6.49
1.44	4.36	4.02	2.32	2.32	0.70	5.06	8.12	8.12	6.67
1.44	4.36	4.36	2.32	2.32	0.70	0.70	8.12	8.12	6.67
255.41	297.86	497.36	118.76	173.92	228.59	364.30	5557.18	9326.80	557.95
1.44	3.69	3.69	2.32	2.32	0.70	0.70	8.12	4.39	1.91
1.44	3.69	5.04	2.32	2.32	0.70	7.56	15.93	37.87	6.67
4.60	2.58	3.04	2.36	7.53	14.94	33.86	10.91	21.44	678.59

2.71	2.58	2.58	2.36	2.36	12.15	12.15	17.64	16.89	807.08
3.69	21.16	22.68	1.56	2.36	6.42	3.45	38.45	29.12	582.00
1.30	2.58	2.58	3.78	2.36	35.84	17.70	1551.00	938.42	1084.84
1.57	2.58	2.58	3.78	3.14	4.75	1.40	10.91	10.91	413.65
1.57	2.58	2.58	2.36	2.36	10.73	0.28	13.88	12.39	538.43
0.55	2.58	2.58	2.36	2.36	12.15	1.40	16.13	24.50	705.09
1.57	2.58	2.58	3.78	2.36	10.73	1.40	13.14	7.96	493.27
3.45	2.58	2.58	3.78	2.36	23.16	6.42	29.12	21.44	950.40
3.45	2.58	2.58	1.56	2.36	24.51	9.31	5.07	10.91	344.80
205.49	564.79	457.72	28.62	26.58	21.80	1.91	38.45	21.44	927.19
2.45	2.58	2.58	2.36	2.36	14.94	14.94	16.89	19.16	624.19
1.57	2.58	2.58	2.36	2.36	12.15	7.88	13.88	12.39	538.43
7.12	2.58	6.17	3.78	7.31	53.42	31.21	22.97	33.77	903.73
5.47	1.76	7.44	5.93	10.15	37.82	41.09	36.89	32.22	1062.95
2.71	22.18	7.44	10.89	3.78	28.54	19.08	39.24	26.03	856.00
3.57	2.58	2.58	2.36	2.36	17.70	25.86	19.92	25.27	903.73
1.57	2.58	2.58	2.36	2.36	1.40	1.40	12.39	6.51	183.01
1.57	2.58	2.58	2.36	1.56	32.53	12.15	24.50	17.64	596.21
1.30	2.58	2.58	8.17	7.31	6.42	4.21	24.50	15.38	567.64
1.57	2.58	2.58	2.36	1.56	19.08	6.42	7.96	19.92	477.82
1.57	4.84	2.58	8.17	3.14	24.51	19.08	26.03	25.27	782.13
3.21	2.58	2.58	3.78	2.36	13.55	6.42	22.97	19.92	880.01
2.05	2.58	2.58	2.42	4.37	16.33	24.51	19.92	24.50	880.01
4.82	2.58	2.58	2.36	2.36	35.18	25.86	22.97	25.27	927.19
2.45	6.82	2.58	1.56	2.36	10.73	16.33	19.92	24.50	831.70
1.57	2.58	2.58	2.36	2.36	22.48	10.73	24.50	19.92	624.19
98.13	471.68	551.50	10.83	9.48	17.11	96.15	517.54	432.49	47.06
11.88	30.98	35.34	13.18	11.50	58.99	64.34	908.83	938.42	85.41
15.33	28.09	48.62	4.00	10.15	89.12	120.62	1417.03	1444.03	133.20
24.82	51.61	75.90	10.15	17.52	57.20	90.88	1126.36	1168.78	90.97
7.96	11.26	16.76	8.46	13.52	0.00	7.62	599.74	631.96	52.47
7.41	57.62	13.99	15.52	2.59	122.36	60.77	1669.52	1182.84	161.77
15.91	0.00	45.64	1.52	14.19	17.11	75.00	695.38	1224.83	47.06
13.03	68.24	28.09	15.52	8.12	99.66	80.31	1307.92	997.02	119.03

TNFpost	IP10pre	IP10post	IL17PRE	IL17POST	filter_$	deltavitd	deltaip10	deltaIL2	deltaIL4
299.09	1556.30	981.09	18.061	13.844	1	31.00	-575.22	0.00	#NULL!
119.03	989.39	976.94	0.000	0.000	0	1.70	-12.45	0.00	#NULL!
12.97	205.20	200.68	0.000	0.000	1	31.80	-4.52	0.86	#NULL!
90.97	1123.69	933.08	0.000	0.000	1	41.30	-190.61	0.00	#NULL!
6.67	268.87	670.44	11.316	13.001	1	18.00	401.57	0.00	-0.17
6.67	1343.45	1430.92	11.316	13.844	0	0.50	87.47	0.00	0.00
0.22	2937.71	1336.54	12.159	13.001	1	42.20	-1601.17	0.00	-0.16
500.79	1262.86	2719.62	11.316	12.159	0	2.30	1456.76	15.99	-5.38
6.67	269.82	493.58	12.159	13.844	0	3.30	223.76	-76.69	-0.18
1.91	800.48	878.02	12.159	13.844	0	4.00	77.54	0.00	0.35
5.00	535.24	543.73	12.159	12.159	1	41.00	8.49	0.00	0.15
3.48	317.19	181.41	9.632	12.159	0	0.60	-135.78	-1.32	-0.31
6.67	396.99	236.65	0.000	0.000	1	23.50	-160.34	0.00	0.00
1.91	1780.11	2136.50	12.159	13.001	0	-3.10	356.39	0.00	-0.07
6.67	376.32	613.65	12.159	14.687	1	11.50	237.33	-50.43	-0.54
61.19	2124.29	1385.96	12.159	11.316	1	30.10	-738.33	1.31	-0.51
0.22	202.75	222.13	10.474	13.001	1	37.70	19.38	-3.29	0.00
1.91	1418.80	1236.20	0.000	0.000	0	1.10	-182.60	0.00	0.00
0.22	1887.03	1235.39	11.316	11.316	0	-3.00	-651.64	0.00	0.30
1205.49	1129.35	933.08	13.001	13.844	1	18.40	-196.27	0.00	-0.12
6.67	1579.93	1605.10	0.000	0.000	0	5.40	25.17	122.48	0.00
6.67	494.95	417.76	11.316	12.159	0	0.30	-77.19	0.00	-0.19
1.09	820.32	737.42	10.474	14.687	1	16.10	-82.90	0.00	0.19
0.22	113.25	223.12	12.159	12.159	1	38.20	109.87	0.00	0.33
15.06	132.32	65.34	12.159	18.905	1	10.70	-66.98	0.00	-0.51
1.91	423.22	865.36	12.159	12.159	0	-2.30	442.14	-3.42	0.16
6.67	895.26	515.07	11.316	13.001	1	22.40	-380.19	0.00	-4.77
6.67	1483.20	867.75	12.159	12.159	1	49.50	-615.45	-77.49	-0.33
250.84	1228.20	780.25	11.316	13.844	0	1.00	-447.95	-0.09	3.06
30.02	236.75	289.19	13.001	13.001	0	3.10	52.44	66.43	-0.45
0.22	485.56	271.83	13.001	13.001	1	28.40	-213.73	-18.05	-0.32
7.96	198.97	111.54	13.844	12.159	1	44.20	-87.43	0.00	-0.79
5.00	269.82	380.73	14.687	15.530	0	-4.80	110.91	-0.82	0.00
1.91	330.14	375.22	12.159	12.159	0	-3.90	45.08	0.00	-0.16
1.91	314.09	270.85	12.159	12.159	0	3.70	-43.24	0.00	-0.28
6.67	181.48	259.79	13.001	12.159	0	0.60	78.31	0.00	-0.79
6.67	972.60	924.43	11.316	13.844	1	23.50	-48.17	4.87	-0.32
2.70	141.42	71.54	15.530	14.687	0	0.70	-69.88	0.00	0.00
6.67	1123.69	730.64	12.159	16.374	0	-0.60	-393.05	-18.89	0.22
6.67	202.35	933.08	12.159	13.001	1	34.90	730.73	0.00	-0.08
6.67	649.78	512.22	13.844	16.374	1	12.70	-137.56	0.00	0.18
6.67	714.84	495.10	41.302	4.687	1	27.70	-219.74	0.00	0.35
872.32	550.43	418.79	0.000	2.331	1	29.80	-131.64	2.48	4.17
1.91	364.41	770.19	4.687	3.509	0	1.50	405.78	151.02	0.08
1.91	817.00	917.42	1.153	4.687	0	2.10	100.42	0.00	0.08
903.73	904.19	737.30	7.043	3.509	1	16.30	-166.89	0.00	0.08

756.83	939.02	879.13	1.153	14.117	0	4.60	-59.89	0.00	2.72
538.43	1822.38	1575.48	2.331	2.331	1	17.40	-246.90	0.00	-0.70
756.83	195.30	269.95	1.153	7.043	0	0.40	74.65	0.00	-2.21
308.06	221.67	149.57	7.043	4.687	0	1.50	-72.10	0.00	-0.18
326.66	355.58	205.87	0.000	3.509	0	2.50	-149.71	0.00	-1.31
326.66	424.79	386.39	3.509	2.331	1	17.20	-38.40	0.00	-3.55
183.01	162.00	416.69	1.153	5.865	0	1.80	254.69	0.00	-2.01
508.52	674.88	1330.81	4.687	8.221	1	7.00	655.93	0.00	-2.80
508.52	819.75	1316.92	1.153	3.509	0	5.20	497.17	0.00	0.92
567.64	455.99	400.68	2.331	3.509	1	14.20	-55.31	0.00	-3.36
678.59	1014.62	943.15	2.331	3.509	0	2.80	-71.47	0.00	-0.49
344.80	289.94	239.28	3.509	2.331	0	4.80	-50.66	0.00	-0.73
1018.58	539.65	256.67	4.687	3.509	1	42.60	-282.98	0.00	2.62
1149.38	505.51	381.04	3.509	2.331	1	55.20	-124.47	0.00	0.08
705.09	820.84	505.15	3.509	7.043	1	43.80	-315.69	-0.27	-1.88
856.00	1276.30	1475.88	4.687	3.509	1	20.90	199.58	0.00	1.52
718.18	460.29	552.03	3.509	5.865	0	-4.10	91.74	0.00	-1.38
756.83	499.10	555.45	2.331	3.509	0	-1.00	56.35	0.00	-2.04
610.27	298.05	539.37	4.687	11.758	1	32.00	241.32	-7.63	-0.34
678.59	329.19	472.05	3.509	2.331	0	-6.40	142.86	0.00	2.79
379.90	597.67	422.70	2.331	148.727	1	17.30	-174.97	0.00	-0.72
430.08	488.57	1046.66	4.687	3.509	0	-1.00	558.09	0.00	-0.56
731.16	366.02	526.21	3.509	7.043	0	-0.20	160.19	0.00	1.14
596.21	180.15	150.14	3.509	3.509	1	-1.20	-30.01	0.00	1.42
1062.95	1028.76	989.43	3.509	3.509	0	1.20	-39.33	0.00	0.97
927.19	163.79	215.24	4.687	4.687	1	21.10	51.45	0.00	-0.99
0.00	415.20	841.16	35.383	9.400	0	1.50	425.96	-29.66	-10.99
96.55	175.39	201.09	7.043	3.509	1	8.50	25.70	0.00	0.94
147.45	259.20	726.58	3.509	2.331	0	-0.70	467.38	0.00	4.75
130.36	1817.40	1778.41	1.153	7.043	1	3.20	-38.99	0.00	5.21
41.70	984.60	630.99	7.043	5.865	0	0.20	-353.61	0.00	1.46
113.39	357.19	100.63	9.400	2.331	1	17.90	-256.56	0.00	-11.64
136.05	1721.20	869.24	16.477	5.865	1	4.00	-851.96	0.00	9.96
93.76	221.45	171.35	8.221	8.221	0	-4.90	-50.10	0.00	-5.02

deltaIL5	deltaIL10	deltaIL12	deltaIL13	deltaIFN	deltaTNF	deltaGMCS	deltaIL17	FIB4CAT	LogIP10pre
#NULL!	#NULL!	#NULL!	#NULL!	#NULL!	#NULL!	#NULL!	-4.22	2	3.19
#NULL!	#NULL!	#NULL!	#NULL!	#NULL!	#NULL!	#NULL!	0.00	2	3.00
#NULL!	#NULL!	#NULL!	#NULL!	#NULL!	#NULL!	#NULL!	0.00	1	2.31
#NULL!	#NULL!	#NULL!	#NULL!	#NULL!	#NULL!	#NULL!	0.00	3	3.05
0.00	-0.57	0.00	1.33	3.73	4.76	0.00	1.69	2	2.43
0.00	1.30	-0.33	1.99	0.00	-5.59	0.00	2.53	3	3.13
0.00	0.00	2.09	0.00	73.58	-1.69	0.00	0.84	2	3.47
-4.87	-38.52	-202.44	-17.04	-3285.57	-311.75	-111.73	0.84	2	3.10
0.00	0.00	-1.39	-0.69	0.00	4.76	0.00	1.69	3	2.43
0.00	0.00	-0.34	0.00	-3.73	1.69	0.00	1.69	2	2.90
0.00	-1.30	0.68	0.00	21.54	4.78	0.41	0.00	2	2.73
0.00	-1.30	-1.36	0.00	-29.75	-5.92	0.00	2.53	3	2.50
0.00	0.00	0.00	0.00	0.00	0.00	0.00	0.00	2	2.60
-1.98	-3.55	-6.84	-2.54	-110.96	-5.32	0.00	0.84	2	3.25
0.00	0.00	0.00	-0.66	-11.54	6.45	0.00	2.53	1	2.58
0.00	-0.90	-0.70	-2.38	-662.92	-28.95	-13.47	-0.84	3	3.33
0.00	0.00	-1.36	-0.54	-11.54	-4.78	2.92	2.53	2	2.31
0.00	0.73	0.00	1.33	11.09	1.69	0.00	0.00	2	3.15
0.00	0.00	0.68	-1.99	11.54	-6.45	0.00	0.00	2	3.28
6.36	35.62	255.88	45.42	2867.30	342.56	6.57	0.84	3	3.05
0.00	0.00	0.00	0.00	0.00	0.00	0.00	0.00	3	3.20
0.00	0.00	0.00	0.00	-5.86	0.00	0.00	0.84	3	2.69
0.00	0.00	0.66	0.00	0.00	-5.58	-2.59	4.21	2	2.91
0.00	0.00	0.67	-0.28	0.00	-6.45	-0.32	0.00	2	2.05
0.00	0.00	-0.35	-1.33	-21.26	13.15	0.00	6.75	3	2.12
0.00	0.00	0.69	0.64	-7.81	1.69	0.00	0.00	2	2.63
-20.20	-25.80	-47.59	-66.21	-4677.05	-410.01	-120.37	1.69	3	2.95
0.00	0.00	-1.37	-3.06	3.73	4.76	0.00	0.00	1	3.17
-17.59	8.74	35.27	51.73	3103.76	235.78	82.78	2.53	2	3.09
0.00	0.00	-1.36	0.00	-66.72	20.62	0.00	0.00	2	2.37
0.00	0.00	-1.36	0.00	3.73	-6.45	0.00	0.00	2	2.69
0.92	0.00	-1.39	-0.46	-50.08	-8.49	0.00	-1.69	3	2.30
0.00	2.19	-0.68	1.22	-11.54	1.52	3.56	0.84	1	2.43
0.00	0.00	-0.68	0.00	0.00	-3.09	0.00	0.00	1	2.52
0.00	-1.04	1.39	1.62	-10.33	-1.57	0.00	0.00	2	2.50
0.00	0.00	-2.75	-1.49	-5.51	0.92	0.00	-0.84	2	2.26
0.00	0.00	0.00	0.00	-20.35	0.00	0.00	2.53	2	2.99
0.00	0.00	-1.43	-1.05	-21.54	-2.30	0.00	-0.84	1	2.15
0.00	0.47	-0.65	0.00	0.00	0.00	0.00	4.22	2	3.05
-1.85	0.00	-2.05	0.00	-20.63	0.18	-1.10	0.84	1	2.31
0.00	0.00	-0.34	0.00	0.00	0.00	4.36	2.53	3	2.81
0.00	0.00	0.00	0.00	0.00	0.00	0.00	-36.62	2	2.85
26.47	109.54	199.50	55.16	3769.62	314.37	135.71	2.33	3	2.74
0.00	0.00	0.00	0.00	-3.73	0.00	0.00	-1.18	2	2.56
0.00	0.00	1.35	0.00	21.94	-4.76	6.86	3.53	2	2.91
4.11	1.03	0.46	5.17	10.53	225.14	18.92	-3.53	2	2.96

-0.34	0.53	0.00	0.00	-0.75	-50.25	0.00	12.96	3	2.97
0.00	0.24	1.52	0.80	-9.33	-43.57	-2.97	0.00	2	3.26
-2.36	-3.30	0.00	-1.42	-11.48	-328.01	-18.14	5.89	3	2.29
0.00	0.00	0.00	-0.64	0.00	-105.59	-3.35	-2.36	1	2.35
0.00	0.00	0.00	0.00	-1.49	-211.77	-10.45	3.51	1	2.55
0.48	-0.41	0.00	0.00	8.37	-378.43	-10.75	-1.18	2	2.63
0.00	0.00	0.00	-1.42	-5.18	-310.26	-9.33	4.71	1	2.21
-3.27	0.49	0.00	-1.42	-7.68	-441.88	-16.74	3.53	3	2.83
0.00	-2.44	0.00	0.80	5.84	163.72	-15.20	2.36	1	2.91
-6.49	-14.60	-107.07	-2.04	-17.01	-359.55	-19.89	1.18	3	2.66
0.00	-2.15	0.00	0.00	2.27	54.40	0.00	1.18	2	3.01
0.00	0.44	0.00	0.00	-1.49	-193.63	-4.27	-1.18	1	2.46
2.36	1.02	3.59	3.53	10.80	114.85	-22.21	-1.18	2	2.73
2.03	1.32	5.68	4.22	-4.67	86.43	3.27	-1.18	2	2.70
-3.31	-1.44	-14.74	-7.11	-13.21	-150.91	-9.46	3.53	2	2.91
1.98	3.02	0.00	0.00	5.35	-47.73	8.16	-1.18	2	3.11
0.00	0.00	0.00	0.00	-5.88	535.17	0.00	2.36	2	2.66
-1.55	-0.88	0.00	-0.80	-6.86	160.62	-20.38	1.18	2	2.70
-1.22	-0.27	0.00	-0.86	-9.12	42.63	-2.21	7.07	2	2.47
0.00	0.00	0.00	-0.80	11.96	200.77	-12.66	-1.18	3	2.52
-3.14	0.27	-2.26	-5.03	-0.76	-402.23	-5.43	146.40	1	2.78
1.12	-1.61	0.00	-1.42	-3.05	-449.93	-7.13	-1.18	2	2.69
3.71	1.50	0.00	1.95	4.58	-148.85	8.18	3.53	3	2.56
2.80	-0.65	0.00	0.00	2.30	-330.98	-9.32	0.00	2	2.26
0.88	-0.76	-4.24	0.80	4.58	231.25	5.60	0.00	3	3.01
0.48	-1.64	0.00	0.00	-4.58	303.00	-11.75	0.00	2	2.21
6.72	8.82	79.82	-1.35	-85.05	-47.06	79.04	-25.98	3	2.62
0.42	-0.57	4.36	-1.68	29.59	11.14	5.35	-3.53	1	2.24
2.54	4.58	20.53	6.15	227.00	14.25	31.50	-1.18	2	2.41
4.02	-0.61	24.29	7.37	42.42	39.39	33.68	5.89	2	3.26
0.63	4.81	5.50	5.06	32.22	-10.77	7.62	-1.18	3	2.99
-3.82	-12.03	-43.63	-12.93	-486.68	-48.38	-61.59	-7.07	2	2.55
7.38	7.95	45.64	12.67	529.45	88.99	57.89	-10.61	3	3.24
-4.66	-2.30	-40.15	-7.40	-310.90	-25.27	-19.35	0.00	1	2.35

LogIP10po	delta_logIF	DPP4pre	DPP4post	deltaDPP4	logDPP4pr	logDPP4po	deltaLogDpp4
2.99	-0.20	8708.89	4182.16	-4526.73	3.94	3.62	-0.32
2.99	-0.01	5302.27	10158.31	4856.04	3.72	4.01	0.28
2.30	-0.01	5795.98	5755.16	-40.82	3.76	3.76	0.00
2.97	-0.08	5989.79	6808.95	819.16	3.78	3.83	0.06
2.83	0.40	5644.82	5187.25	-457.57	3.75	3.71	-0.04
3.16	0.03	7770.14	8512.27	742.13	3.89	3.93	0.04
3.13	-0.34	7758.21	5525.62	-2232.59	3.89	3.74	-0.15
3.43	0.33	4505.98	7317.23	2811.25	3.65	3.86	0.21
2.69	0.26	6076.85	5116.19	-960.66	3.78	3.71	-0.07
2.94	0.04	5723.21	7772.88	2049.67	3.76	3.89	0.13
2.74	0.01	7897.34	7729.52	-167.82	3.90	3.89	-0.01
2.26	-0.24	8841.33	8066.10	-775.23	3.95	3.91	-0.04
2.37	-0.22	6621.44	6094.03	-527.41	3.82	3.78	-0.04
3.33	0.08	6559.83	7944.89	1385.06	3.82	3.90	0.08
2.79	0.21	5193.72	4758.64	-435.08	3.72	3.68	-0.04
3.14	-0.19	3948.31	3484.60	-463.71	3.60	3.54	-0.05
2.35	0.04	9542.48	8803.11	-739.37	3.98	3.94	-0.04
3.09	-0.06	3948.31	4930.19	981.88	3.60	3.69	0.10
3.09	-0.18	6264.83	5745.16	-519.67	3.80	3.76	-0.04
2.97	-0.08	6190.24	6455.02	264.78	3.79	3.81	0.02
3.21	0.01	5991.18	6496.15	504.97	3.78	3.81	0.04
2.62	-0.07	5332.02	4246.69	-1085.33	3.73	3.63	-0.10
2.87	-0.05	7798.98	5328.30	-2470.68	3.89	3.73	-0.17
2.35	0.29	6926.32	6415.40	-510.92	3.84	3.81	-0.03
1.82	-0.31	5978.39	5063.05	-915.34	3.78	3.70	-0.07
2.94	0.31	6104.46	4912.01	-1192.45	3.79	3.69	-0.09
2.71	-0.24	8535.65	7463.34	-1072.31	3.93	3.87	-0.06
2.94	-0.23	4916.67	4846.74	-69.93	3.69	3.69	-0.01
2.89	-0.20	5226.67	5227.60	0.93	3.72	3.72	0.00
2.46	0.09	6906.79	6110.99	-795.80	3.84	3.79	-0.05
2.43	-0.25	5716.00	4920.45	-795.55	3.76	3.69	-0.07
2.05	-0.25	4742.69	5226.47	483.78	3.68	3.72	0.04
2.58	0.15	3871.11	4332.91	461.80	3.59	3.64	0.05
2.57	0.06	5755.18	5488.57	-266.61	3.76	3.74	-0.02
2.43	-0.06	4653.26	5630.49	977.23	3.67	3.75	0.08
2.41	0.16	5647.13	5586.11	-61.02	3.75	3.75	0.00
2.97	-0.02	4445.71	4319.97	-125.74	3.65	3.64	-0.01
1.85	-0.30	6266.42	5800.14	-466.28	3.80	3.76	-0.03
2.86	-0.19	6778.28	5385.49	-1392.79	3.83	3.73	-0.10
2.97	0.66	4442.01	4348.17	-93.84	3.65	3.64	-0.01
2.71	-0.10	6915.59	7196.63	281.04	3.84	3.86	0.02
2.69	-0.16	5595.82	5383.64	-212.18	3.75	3.73	-0.02
2.62	-0.12	4442.01	5189.49	747.48	3.65	3.72	0.07
2.89	0.33	4962.05	7163.35	2201.30	3.70	3.86	0.16
2.96	0.05	5501.51	5870.86	369.35	3.74	3.77	0.03
2.87	-0.09	7179.53	7002.48	-177.05	3.86	3.85	-0.01

2.94	-0.03	7637.63	7542.40	-95.23	3.88	3.88	-0.01
3.20	-0.06	7435.70	5579.64	-1856.06	3.87	3.75	-0.12
2.43	0.14	6204.06	9199.15	2995.09	3.79	3.96	0.17
2.17	-0.17	5078.90	4935.39	-143.51	3.71	3.69	-0.01
2.31	-0.24	3000.50	2674.65	-325.85	3.48	3.43	-0.05
2.59	-0.04	6486.13	7115.95	629.82	3.81	3.85	0.04
2.62	0.41	5090.56	7405.22	2314.66	3.71	3.87	0.16
3.12	0.29	6746.04	7848.27	1102.23	3.83	3.89	0.07
3.12	0.21	4938.67	4769.89	-168.78	3.69	3.68	-0.02
2.60	-0.06	5969.64	4854.75	-1114.89	3.78	3.69	-0.09
2.97	-0.03	5762.29	4663.93	-1098.36	3.76	3.67	-0.09
2.38	-0.08	6709.47	6932.16	222.69	3.83	3.84	0.01
2.41	-0.32	3880.97	5650.83	1769.86	3.59	3.75	0.16
2.58	-0.12	8790.98	6309.55	-2481.43	3.94	3.80	-0.14
2.70	-0.21	5034.31	6340.02	1305.71	3.70	3.80	0.10
3.17	0.06	3858.00	3535.91	-322.09	3.59	3.55	-0.04
2.74	0.08	8228.93	7556.66	-672.27	3.92	3.88	-0.04
2.74	0.05	4941.48	4271.98	-669.50	3.69	3.63	-0.06
2.73	0.26	8490.58	5521.43	-2969.15	3.93	3.74	-0.19
2.67	0.16	5266.22	4310.43	-955.79	3.72	3.63	-0.09
2.63	-0.15	5595.82	5311.86	-283.96	3.75	3.73	-0.02
3.02	0.33	3148.65	4124.30	975.65	3.50	3.62	0.12
2.72	0.16	8079.30	8542.66	463.36	3.91	3.93	0.02
2.18	-0.08	9102.82	6618.18	-2484.64	3.96	3.82	-0.14
3.00	-0.02	8453.69	7493.03	-960.66	3.93	3.87	-0.05
2.33	0.12	5695.84	8935.67	3239.83	3.76	3.95	0.20
2.92	0.31	10333.60	11064.53	730.93	4.01	4.04	0.03
2.30	0.06	7174.52	7931.41	756.89	3.86	3.90	0.04
2.86	0.45	7070.10	7096.19	26.09	3.85	3.85	0.00
3.25	-0.01	8228.93	7449.09	-779.84	3.92	3.87	-0.04
2.80	-0.19	8528.82	7140.61	-1388.21	3.93	3.85	-0.08
2.00	-0.55	7779.72	6919.50	-860.22	3.89	3.84	-0.05
2.94	-0.30	10969.68	7884.61	-3085.07	4.04	3.90	-0.14
2.23	-0.11	9652.93	8795.27	-857.66	3.98	3.94	-0.04
